# Establishing long-term nitrogen response of global cereals to assess sustainable fertilizer rates

**DOI:** 10.1038/s43016-021-00447-x

**Published:** 2022-01-31

**Authors:** Hans J. M. van Grinsven, Peter Ebanyat, Margaret Glendining, Baojing Gu, Renske Hijbeek, Shu Kee Lam, Luis Lassaletta, Nathaniel D. Mueller, Felipe S. Pacheco, Miguel Quemada, Tom W. Bruulsema, Brian H. Jacobsen, Hein F. M. ten Berge

**Affiliations:** 1 Department of Water, Agriculture and Food, PBL Netherlands Environmental Assessment Agency, The Hague, Netherlands; 2 Department of Agricultural Production, School of Agricultural Sciences, Makerere University, Kampala, Uganda; 3 Department of Computational and Analytical Sciences (CAS), Rothamsted Research, Harpenden, UK; 4 College of Environmental and Resource Sciences, Zhejiang University, Hangzhou, PR China; 5 Plant Production Systems Group, Wageningen University, Wageningen, Netherlands; 6 School of Agriculture and Food, Faculty of Veterinary and Agricultural Sciences, The University of Melbourne, Melbourne, Victoria Australia; 7 Department Agricultural Production/CEIGRAM, Universidad Politécnica de Madrid, Madrid, Spain; 8 Department of Ecosystem Science and Sustainability, Department of Soil and Crop Sciences, Colorado State University, Fort Collins, CO USA; 9 National Institute for Space Research (INPE), Earth System Science Center, São José dos Campos, Brazil; 10 Plant Nutrition Canada, Guelph, Ontario Canada; 11 University of Copenhagen, Department of Food and Resource Economics (IFRO), Copenhagen, Denmark; 12 Wageningen Plant Research, Wageningen, Netherlands

**Keywords:** Environmental sciences, Environmental social sciences, Agriculture

## Abstract

Insight into the response of cereal yields to nitrogen fertilizer is fundamental to improving nutrient management and policies to sustain economic crop benefits and food sufficiency with minimum nitrogen pollution. Here we propose a new method to assess long-term (LT) regional sustainable nitrogen inputs. The core is a novel scaled response function between normalized yield and total net nitrogen input. The function was derived from 25 LT field trials for wheat, maize and barley in Europe, Asia and North America and is fitted by a second-order polynomial (*R*^2^ = 0.82). Using response functions derived from common short-term field trials, with soil nitrogen not in steady state, gives the risks of soil nitrogen depletion or nitrogen pollution. The scaled LT curve implies that the total nitrogen input required to attain the maximum yield is independent of this maximum yield as postulated by Mitscherlich in 1924. This unique curve was incorporated into a simple economic model with valuation of externalities of nitrogen surplus as a function of regional per-capita gross domestic product. The resulting LT sustainable nitrogen inputs range from 150 to 200 kgN ha^−1^ and this interval narrows with increasing yield potential and decreasing gross domestic product. The adoption of LT response curves and external costs in cereals may have important implications for policies and application ceilings for nitrogen use in regional and global agriculture and ultimately the global distribution of cereal production.

## Abstract (web summary)

Quantifying the long-term (LT) response of crop yields to nitrogen fertilizer is critical to improving nutrient management practices. Based on 25 LT field experiments, this study develops a generic LT nitrogen response function for global cereals to characterize the yield impacts, associated LT economic benefits and external costs of changing nitrogen inputs.

## Main

Finding a balance between the benefits of nitrogen fertilizer use for food production and the impacts of agricultural nitrogen pollution on human health and ecosystems is a challenge from the regional to the global scale^1,2^. Current global anthropogenic addition of new nitrogen from the Haber–Bosch process, cultivation of nitrogen-fixing crops and combustion of fossil fuels more than doubles the natural input of reactive nitrogen^3^, thereby exceeding the assumed planetary boundary of nitrogen^4^ and causing high environmental costs^3,5^. The use of synthetic fertilizers and manures across the nearly 40% of Earth’s ice-free land devoted to agriculture^6^ comprises the largest source of ammonia, nitrate and nitrous oxide pollution globally, with severe impacts on ecosystems, human health and climate change^3,7^.

A pivotal relationship for improving the agronomic and environmental performance of food systems is the response of crop yield to addition of nitrogen fertilizer. This relationship sets the yield increase per unit of fertilizer input (known as the agronomic efficiency (AE)) and is the basis for estimating nitrogen surplus and nitrogen use efficiency (NUE, the ratio of nitrogen removed by crops to nitrogen input^8^). The nitrogen response curve, together with prices for crops and nitrogen fertilizers, informs farmers about how much nitrogen fertilizer they need to apply in a given year to obtain the most profitable crop yield, and informs strategies for developing regions to achieve food security without depleting soil nitrogen^9,10^. In today’s increasingly globalized agricultural markets, profit margins for most crops are narrow and farmers struggle to achieve a consistent return on investment^11,12^. Proper management of nutrient resources is a relevant factor in this quest, but the societal costs of nitrogen pollution are only rarely incorporated into economic decisions for farming. Establishing policies that promote societally optimal nitrogen rates, often substantially lower than optimal rates for private economic returns^13,14^, also relies upon accurate characterization of nitrogen–yield responses.

Long-term field experiments (LTEs) are essential to quantify the nitrogen response for assessment of economic and environmental performance of alternative nutrient management practices^15–17^, because the time to establish steady state between nitrogen input, crop yield, nitrogen losses and the soil nitrogen pool can exceed decades, depending on soil organic matter fraction and quality (for example, C/N ratio) and the history of fertilizer use^15^. Although numerous short-term experiments (STEs; single-year trials) are carried out across the globe to inform extension activities, LTEs represent a substantial investment of research time and resources, and are therefore scarce for areas such as Latin America and Africa^18^. STEs can inform yearly management decisions but they cannot correctly characterize the long-term impacts, and associated costs and benefits, of changing nitrogen management policies. Improved understanding of LT nitrogen response functions across regions is critically needed and here we address this knowledge gap.

Drawing upon the generic principles governing nitrogen transformations and uptake when nitrogen input, crop yield and soil nitrogen pools are in near steady state, in this article we propose a generic LT nitrogen response relationship for cereals that can be used to inform policy decisions. We focus on the three major global staple cereals, wheat (16% of global crop area for 2013–2017^19^), maize (14%), barley (3.4%), and also address lowland rice (12.5%). We collect and analyse a global set of LTEs for Europe, North America and Asia, and use the Broadbalk long-term wheat experiment in the United Kingdom (which began in 1843)^20^ to establish a conceptual model describing the differences between ST and LT nitrogen responses.

## Results

### Effects of duration and rotation on the nitrogen response of wheat in the Broadbalk experiment

**Fig. 1:**
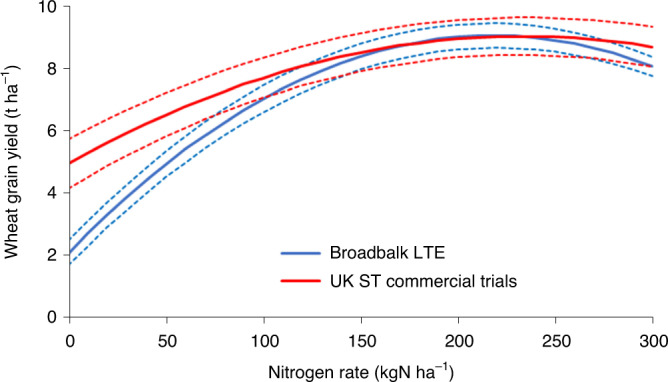
The LT and ST nitrogen response for winter wheat in rotation in the United Kingdom. The LT nitrogen response as observed in the Broadbalk LTE (*N* = 245, *R*^2^ = 0.84) and the common first-year nitrogen response for representative commercial sites in different parts of the United Kingdom (*N* = 105, *R*^2^ = 0.55). Dashed lines, 95% confidence intervals. See also Supplementary Note 3. Source data

Over the past 175 years the combined effects of different amounts of nitrogen–phosphorus–potassium fertilization, use of manures, improved cultivars, pesticides, liming and fallowing has been demonstrated and explained by analysis of crop and soil characteristics in the Broadbalk LTE (Rothamsted, UK)^20^. The LT response of wheat in rotation (Fig. 1) was taken from observations in the Broadbalk experiment from 1985 to 2018, where plots were given the same annual nitrogen fertilizer rate (nitrogen rate) every year^21^. This LT response was compared to the ST (first year after adjustment of nitrogen rate) response at commercial wheat trials in different parts of England between 1994 and 1998 (Supplementary Note 2).

Grain yield at zero nitrogen input (*Y*_0_) for the commercial STEs (5.0 t ha^−1^) is substantially higher than for the Broadbalk LTE (1.7 t ha^−1^) because more nitrogen is available from fertilizer residues and mineralization of the soil organic matter and crop residues originating from previous higher fertilizer inputs. For LTEs such as Broadbalk, the mean net supply of nitrogen from the soil is low and the dominant nitrogen sources for *Y*_0_ are nitrogen deposition (DEP) and natural biological nitrogen fixation (BNF) from free-living bacteria. However, the mean maximum attainable yields (*Y*_max_) for the LTEs and STEs both converge to 9.1 t ha^−1^. The *Y*_max_ for LTEs for continuous wheat in the Broadbalk experiment is lower (Supplementary Note 2).

**Fig. 2:**
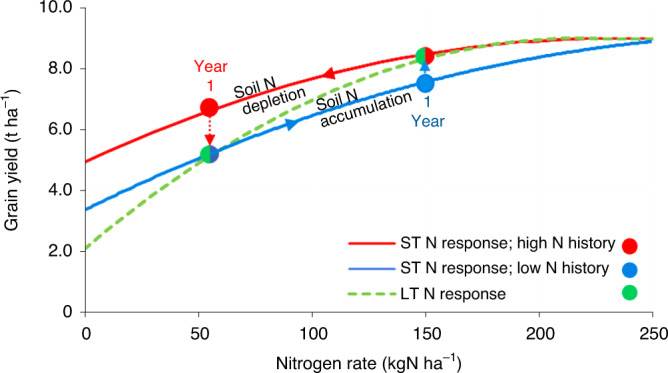
Conceptual illustration of ST and LT nitrogen response curves. ST nitrogen response of wheat grain yields in the United Kingdom in a trial with a history of high nitrogen inputs (150 kgN ha^−1^ (ref. ^25^); red line), and a ST nitrogen response in a trial with a history of low nitrogen inputs (60 kgN ha^−1^; blue line), as compared to the LT generic nitrogen response where soil nitrogen is in steady state for all nitrogen inputs (dashed green line, Broadbalk LTE). As time passes, both ST responses will converge into the single LT response due to changes in soil status as indicated by the arrows. The dots with two colors (either green and blue or green and red) indicate a ST response which overlaps with the LT response when historic and current nitrogen rates are the same.

With continued application of a certain nitrogen rate to soil-cropping systems, ST nitrogen response curves gradually shift to the LT nitrogen response (Fig. 2). When the LT nitrogen rate is higher than the historic rate, grain yields will gradually increase due to soil nitrogen accumulation, causing increased soil nitrogen delivery through greater returns of mineralized nitrogen from nitrogen in roots and crop residues to the soil^21,22^, and an overall improved soil fertility and quality. When the LT nitrogen rate is lower than the historic rate, grain yields will gradually decrease due to soil nitrogen depletion causing decreased soil nitrogen delivery from mineralization. The first case typically applies to developing regions in Africa and south Asia where nitrogen rates are increased to meet the market demand or reduce regional food and feed insecurity; the second case applies to industrialized regions such as Europe where environmental policies restrict nitrogen fertilizer use to reduce nitrogen pollution^23,24^.

### Scaling the annual LT nitrogen response of wheat in rotation in the Broadbalk experiment

**Fig. 3:**
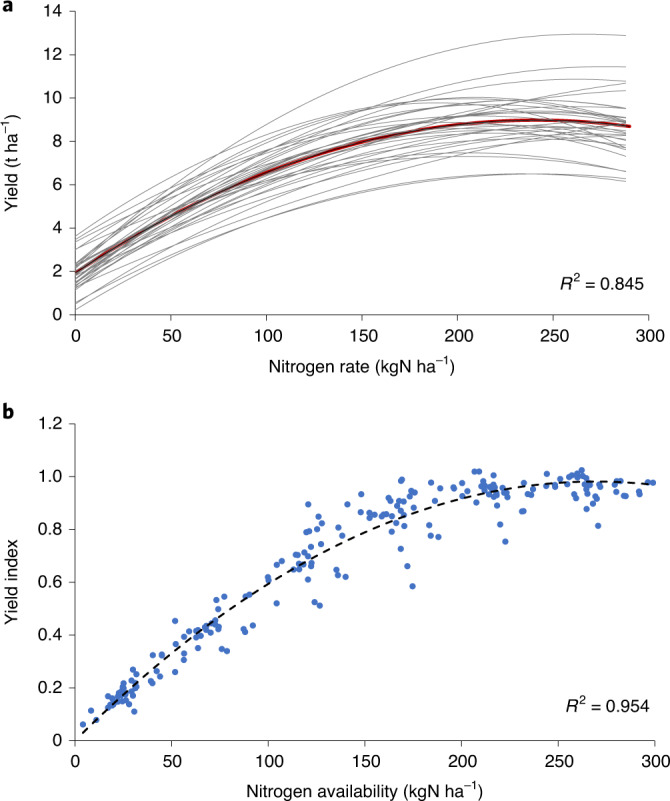
Effect of scaling on annual nitrogen response curves from 1985 to 2018 for winter wheat in rotation in the Broadbalk experiment. **a**, Second-order polynomial fits of annual nitrogen response curves from unscaled observations and mean curve (red). **b**, Yields indexed to maximum annual yield and nitrogen rates per year of observation transformed to available nitrogen by adding estimates of non-fertilizer sources, and fitted with a second-order polynomial with zero intercept (equation (1), black dashed line). Source data

Given its long duration, the soil nitrogen and carbon status at every rate in the Broadbalk experiment is in near steady state with its constant annual nitrogen rate. Every observation year between 1985 and 2018 delivers a nitrogen response curve with its own *Y*_max_, which ranges between 6 and 12 t ha^−1^, and a *Y*_0_ ranging between 0.23 and 3.62 t ha^−1^ (Fig. 3a). Differences in *Y*_max_ reflect differences in annual weather conditions and changes in cultivars (new cultivars were introduced in 1991 and 2013), while *Y*_0_ is also affected by annual nitrogen mineralization.

We hypothesize that the observed LT nitrogen response can be approximated with a single curve, which describes the relative (normalized) yield (*Y*_r_ = *Y*/*Y*_max_ = yield index) in steady state as a function of total available nitrogen (*N*_av_). *N*_av_ is defined as the sum of nitrogen from fertilizers and soil nitrogen supply, including atmospheric deposition (DEP) and biological fixation (BNF). The amount of available nitrogen from soil, DEP and BNF is referred to as SN, which governs crop production in unfertilized plots. This curve in Fig. 3b was derived by first scaling observed annual yields to the annual *Y*_max_, and then converting *N*_rate_ to *N*_av_ by addition of SN (Supplementary Note 3). For each observation year, both *Y*_max_ and SN were estimated from a second-order polynomial fit to the observations, where SN is the intercept on the horizontal axis. SN ranged between 4 and 64 kgN ha^−1^. Mean SN is 30 kgN ha^−1^ and shows no trend in time; with a local DEP of 20 kgN ha^−1^ in 2017, this would suggest a BNF of 10 kgN ha^−1^, which is in accordance with averages for the United Kingdom^26^. Finally, all scaled observations were fitted again with a second-order polynomial with zero intercept (Fig. 3b). The resulting nitrogen response relationship for Broadbalk wheat in rotation is expressed by:

1 $$\begin{array}{rcl}Y_{\mathrm{r}} & = & (- 1.354 \times 10^{-5} \times N_{\mathrm{av}}^{2})\\ && + (7.291 \times 10^{-3} \times N_{\mathrm{av}}) \quad (R^2 = 0.954)\end{array}$$

The same procedure was applied to observations for continuous wheat in the Broadbalk LTE and gives an almost identical quadratic yield response (*R*^2^ = 0.903; Supplementary Note 3). One LTE of relatively long duration (since 1961) for maize in Nebraska (United States) was comparable in set-up to Broadbalk (six nitrogen and three phosphorus rates)^27^. As for Broadbalk, the scaled annual nitrogen response data fit a quadratic function (*R*^2^ = 0.934; see Supplementary Note 4 for details). On average, *Y*_r_ at a given *N*_av_ for maize in the United States is somewhat (11%) higher than for winter wheat in the United Kingdom, indicating that maize has a stronger nitrogen response than wheat. Maize is a C4 crop with nitrogen dilution different from that in C3 crops such as wheat^28^, causing the grain nitrogen content in maize to be somewhat lower (1.5% versus 1.9% (ref. ^29^)). Furthermore, the harvest index of maize tends to be somewhat higher^30^.

Back-transformation of the scaled curves to the nitrogen response curves, as needed to plan regional long-term nitrogen requirements for a certain cereal yield target, would require independent estimates of site-specific SN and *Y*_max_.

### A generic LT nitrogen response for global wheat, maize and barley

**Fig. 4:**
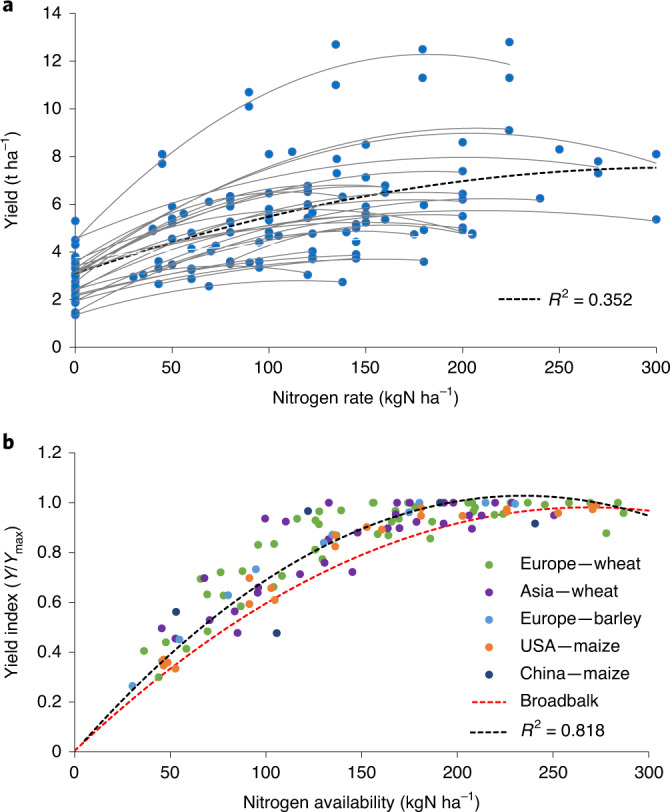
The LT nitrogen response for global wheat, maize and barley. Unscaled (**a**) and scaled (**b**) LT nitrogen response data for wheat, barley and maize trials in Europe, North America and Asia and second-order polynomial fit with zero intercept, as compared to the fit for Broadbalk wheat in rotation. Source data

We next seek to examine whether the LT nitrogen response established in the Broadbalk LTE holds for other cereals in experiments around the globe. The transformations using individual *Y*_max_ and SN values that allowed coalescence of data from the respective years in the Broadbalk LTE into a single curve were also applicable to a set of 25 global LTEs for wheat, maize and barley in Europe, North America and Asia. These 25 LTEs cover a wide range of soils, climates and practices, with nitrogen rates ranging from 0 to 300 kgN ha^−1^ and *Y*_max_ from 2.8 to 12.8 t ha^−1^ (Table 1 and Fig. 4a). The second-order polynomial fit of pooled scaled nitrogen response data for global cereals was:

2 $$\begin{array}{rcl}Y_{\mathrm{r}} & = & (- 1.870 \times 10^{-5} \times N_{\mathrm{av}}^2) \\ && + (8.768 \times 10^{-3} \times N_{\mathrm{av}})\quad (R^2 = 0.818)\end{array}$$

**Table 1:** Overview of the characteristics of the LT nitrogen trials used

Experiment	Region	Crop	Type	Start year and period used	Key reference
**Winter wheat**					
Broadbalk	United Kingdom	In rotation and continuous	Field, seven nitrogen rates	1843; 1985–2018	Johnston et al. 2018
Müncheberg	Germany	In rotation	Field, five nitrogen rates	1962; 1984–2002	Hijbeek et al. 2017
Limburgerhof	Germany	In rotation	Field, five nitrogen rates	1987; 1987–1994	Lang et al. 1995
Oldenburg	Germany	In rotation	Field, five nitrogen rates	1984; 1985–1993	Klasink and Steffens 1995
Rauischholzhausen	Germany	In rotation	Field, five nitrogen rates	1984	Von Boguslawski 1995
Speyer	Germany	In rotation	Field, five nitrogen rates	1984; 1994–1999	Bischoff 1995
Spröda	Germany	In rotation	Field, five nitrogen rates	1966; 1999–2010	Albert and Grunert 2013; Körschens et al. 2014
Grabow	Poland	In rotation	Field, four nitrogen rates	1980–current	Rutkowska and Skowron 2020
India, Pakistan, Bangladesh	South Asia		Nine sites, 16 field trials	1982–2008	Jat et al. 2014
Laiyang, Shandong	China	Maize–wheat rotation	Field, three nitrogen rates	1978–2013	B. Gu, personal communication
**Winter barley**					
Oldenburg	Germany	In rotation	Field, five nitrogen rates	1984; 1985–1993	Klasink and Steffens 1995
Speyer	Germany	In rotation	Field, five nitrogen rates	1984; 1994–1999	Bischoff 1995
**Maize**					
Wisconsin	USA	In rotation and continuous	Field, four nitrogen rates, 28 rotations, two replicates	1968; 1990–2004	Stanger et al. 2006
Kansas	USA	Irrigated continuous	Field, six nitrogen rates	1961; 1997–2006	Schlegel et al. 2017
Iowa	USA	Maize–soybean	Field + model, seven nitrogen rates	1996–2005	Thorp et al. 2007
Changping	China	Irrigated continuous	Field, three nitrogen rates	1984, 2011–2012	Wen et al. 2016
Laiyang, Shandong	China	Maize wheat rotation	Field, three nitrogen rates	1978–2013	B. Gu, Personal communication
Lossa, Konni	Niger	Maize, millet, sorghum,	Field, five nitrogen rates, not a LTE	1997–1998	Pandey et al. 2001
Chikwawa	Malawi	Irrigated maize–rice two-crop system	Field, four nitrogen rates, not a LTE	2007	Fandika et al. 2008
Sub-Saharan Africa	Nine countries	Continuous	Model supported by field data	Used for validation	Ten Berge et al. 2019
**Rice–wheat double-cropping systems**					
Parwanipur	Nepal	Irrigated	Field, four nitrogen rates	1980–2000	Gami et al. 2001
Bhairahawa	Nepal	Irrigated	Field, three nitrogen rates	1978–2013	Rawal et al. 2017
Ludhiana, Punjab	India	Irrigated	Field, four nitrogen rates	1984–1997	Bhandari et al. 2002
Bidhan, West Bengal	India	Irrigated	Field, two nitrogen rates	1986–2004	Majumbar et al. 2008

For details, full references and data, see Supplementary Tables 1 and 2.

The maximum SN was 88 kgN ha^−1^. The nitrogen response in equation (2) is very similar to Broadbalk wheat in rotation (equation (1) and Fig. 4b), but somewhat steeper, which could be the effect of maize in the United States (Supplementary Note 4).

Despite their empirical nature, models of the quadratic form $Y_{\mathrm{r}}=(a \times N_{\mathrm{av}}^2) + (b \times N_{\mathrm{av}})$ do satisfy some ‘general principles of biology’:

1. Relative yield, *Y*_r_ = 0 for *N*_av_ = 0; in the long term there cannot be dry matter production (by photosynthesis) without nitrogen.
2. The law of diminishing returns; with increasing nitrogen inputs the internal nitrogen concentration increases and the dry matter production per unit of nitrogen input (AE) decreases; the quadratic coefficient represents the rate of diminishing returns.
3. The presence of an *Y*_max_ induced by other negative feedbacks at high biomass or high tissue nitrogen concentrations; in cereals examples of such feedbacks are lodging or increased pest incidence.

The validity of the LT generic nitrogen response curve was verified by back-calculation of the original unscaled cereal grain yields for every LTE, using 25 alternative second-order polynomial fits of the dataset of indexed yield as a function of *N*_av_, each time leaving out the observations for the validation site. The high correlation (0.945) between original and back-calculated yields (Supplementary Note 6) and the low root mean square error of the prediction (0.52 t ha^−1^) shows that our generic curve indeed represents the local nitrogen response of cereal yields across a wide range of soils and climates and suggests that it can be used as a first approach for regions where LT curves are not available, such as in Africa and Latin America, provided that local SN and *Y*_max_ are known. Because we found a similar scaled nitrogen response as that in the Broadbalk LTE for the 25 global LTEs, *Y*_max_ can signify either mean attainable yield over many years, or the attainable yield for a given year, depending on the purpose.

In general, fertilizer rates and practices in countries change slowly and hence soils can be expected to be in near-equilibrium with nitrogen rates. Therefore we sought confirmation of our generic LT response curve using national data on crop yields and nitrogen fertilizer use. Modelled yield responses in Europe corresponded well with national data for rain-fed wheat, barley and maize^31^ (*Y*_max_ at 90% of *Y*_w_; *R*^2^ = 0.796; Supplementary Fig. 10) and for wheat^32^ (*R*² = 0.579; Supplementary Fig. 11). In developing countries these national data are often absent or unreliable. As an alternative, we compared maize response in nine countries in sub-Saharan Africa^10^, using modelled nitrogen requirements for target yields according to the Global Yield Gap Atlas^33^, with *Y*_max_ set to 80% of water-limited yield potential (*Y*_w_). We found a reasonable fit (*R*^2^ = 0.671; Supplementary Fig. 12), although in this sub-Saharan Africa study the modelled nitrogen requirements were proportional to *Y*_max_, which is not the case in our generic response curve. The applicability of the generic curve for Africa was further verified against trial results in Niger and Malawi (Supplementary Figs. 13–15).

Notably, the LT generic nitrogen response curve seems not to be applicable for lowland rice. A first provisional scaled nitrogen response curve based on four LT trials for lowland rice in India and Nepal, with wheat as a winter crop as common in South Asia, showed a much weaker increase of yields with *N*_av_ (Supplementary Note 8) and no *Y*_max_, probably because of higher rates of non-symbiotic BNF as compared to wheat and maize^34^, high ammonia loss from urea fertilizers and redox conditions promoting denitrification, all factors weakening yield response to nitrogen fertilizer addition^35,36^.

### A generic model for the AE of global cereals

**Fig. 5:**
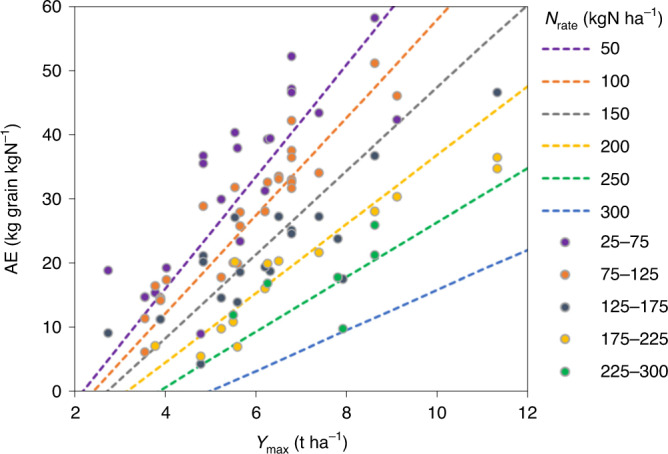
Agronomic efficiency for global cereals. AE increases with *Y*_max_ and decreases with increasing *N*_rate_ (visualized with isolines of applied nitrogen fertilizer with *Y*_0_ set at the mean observed value in LTEs of 2.9 t ha^−1^). Data points are unscaled observations for the 25 LTEs, with AE at observed *N*_rate_ adjusted to the nearest *N*_rate_ isoline using equation (3). Source data

The very close relation between absolute nitrogen rate and relative yield implies that the amount of fertilizer nitrogen required to produce, for example, 70% of *Y*_max_ is fixed, that is, is independent of the value of *Y*_max_ itself. This might be unexpected and is only possible if the conversion of fertilizer nitrogen into grain biomass becomes more efficient with rising *Y*_max_. The agronomic use efficiency of the applied nitrogen (AE = (*Y* − *Y*_0_)/*N*_rate_) expresses the efficiency with which applied nitrogen is converted to grain yield^37^. The long-term AE is achieved when the soil nitrogen supply is in steady state with the annual input rate^10^, and can be estimated from LTEs. For the ensemble of 25 LTEs for wheat, maize and barley in Europe, North America and Asia, the AE was calculated for each *N*_rate_ and LTE and could be fitted accurately as a linear function of *Y*_0_, *Y*_max_, *N*_rate_ and two interaction terms (equation (3) and Fig. 5).

3 $$\begin{array}{rcl}{\mathrm{AE}} & = & 4.62 - (8.37 \times Y_0) + (9.84 \times Y_{\mathrm{max}}) - (0.0365 \times N_{\mathrm{rate}}) \\ && + (0.0172 \times Y_0 \times N_{\mathrm{rate}}) - (0.0223 \times Y_{\mathrm{max}} \times N_{\mathrm{rate}}) \\ && (R^2 = 0.924,\,N = 94)\end{array}$$

This expression explains the positive impact of *Y*_max_ and the negative impact of nitrogen rate on the NUE, the latter causing diminishing returns from nitrogen input (Fig. 1). The positive impact of *Y*_max_ exemplifies Liebscher’s law^38^ that the use efficiency of a yield-constraining factor (here nitrogen) increases as the other factors become more optimal (here expressed in *Y*_max_, which comprises genotype, weather, soil and crop husbandry factors). De Wit^39^ demonstrated that Liebscher’s law holds for nitrogen responses in various crops, and showed that it relies on higher efficiencies in both nitrogen uptake and conversion into grain, at higher *Y*_max_. Taken to its extreme, this law leads to Mitscherlich’s assumption^40^ that the activity coefficient for any nutrient in the exponent of his response function is independent of other factors. In the words by De Wit on Mitscherlich: ‘This heroic assumption … implies that the absolute amount of nutrient needed to reach a certain fraction of the maximum yield is the same whether yields are low or high. Of course, such universal constants do not exist, but this does not exclude the possibility that constant activities manifest themselves in more restricted domains and yield ranges.’ Our present analysis of LTE data indicates the existence of this (near) constancy of nitrogen requirement. We find that the nitrogen requirement to reach *Y*_max_ (or a certain fraction thereof) is uncorrelated with *Y*_max_ itself.

The AE function in equation (3) can be converted to a *Y*_r_ relation with *N*_rate_. AE models derived for STEs also approximate the long-term response when using low values of *Y*_0_ (see Supplementary Note 7 for an example of an STE for maize in Nebraska). Global application of AE models for derivation of long-term sustainable nitrogen inputs requires *Y*_0_ data, which are increasingly available but not for all regions. In addition, *Y*_0_ depends on DEP, which changes over time. Regional SN, required for equation (2), is available from global models^26^.

### Implications of a generic LT nitrogen response

Our finding of a generic nitrogen response curve for wheat, maize and barley in Europe, the United States, South Asia and China, with *Y*_max_ ranging between 2 and 13 t ha^−1^, implies that the *Y*_max_ is attained in a fairly narrow range of *N*_av_. This *N*_av_ at *Y*_max_ (referred to as *N*_max_) is found by solving equation (2) for d*Y/*d*N*_av_ = 0. *N*_max_ for equation (1) was 234 kgN ha^−1^, and the mean *N*_max_ for the 25 LTEs was 217 kgN ha^−1^ (s.d., 41 kgN ha^−1^). The observed range of *N*_max_ across the 25 sites is 143–307 kgN ha^−1^ and *N*_max_ is uncorrelated with *Y*_max_ (*R*^2^ = 0.055). This implies that AE (and also NUE) at given relative yield *Y*_r_ increases with *Y*_max_. One explanation for a high *Y*_max_ in a specific region or year is a good synchrony between crop nitrogen demand and nitrogen availability from soil and fertilizer^41^. This leads to high AE when nitrogen fertilizer rates are not excessive^42^ (Fig. 5). One could also reason that a high *Y*_max_ is the result of a favourable climate, crop physiology and crop–soil system, including a well-functioning root system, allowing maximum interception and utilization of available nitrogen in addition to water and other nutrients.

The hypothesis of NUE increasing with *Y*_max_ could only be tested for nitrogen trials in the Broadbalk LTE, where the nitrogen content of grain and straw were also measured (Supplementary Note 9). For a nitrogen rate range of 144–288 kgN ha^−1^, similar to that giving *Y*_max_ for global cereals, NUE increased substantially (*R*^2^ = 0.166, *N* = 64), and almost proportionally with *Y*_max_, with an average NUE of 40% for an *Y*_max_ of 6 t ha^−1^, increasing to 80% at 10 t ha^−1^.

### Economic optimal nitrogen rates for cereals using generic LT nitrogen response

Farmers need insight into the marginal response of yield to nitrogen rate to determine the economic optimal nitrogen rate (EONR^43^), which is lower than *N*_max_ due to the cost of nitrogen fertilizer. Our EONR applies to the scale of national or regional cereal sectors. Marginal response depends on the time horizon of optimization and the choice of response curve. The net economic return is the gross return from crop sales minus the costs of labour, capital and nitrogen fertilizer inputs, and depends on prevailing prices of grain and nitrogen fertilizer. The nitrogen rate giving maximum financial return is a proxy for the mean optimal nitrogen rate for regional or national cereal farming. Taking into account the external cost of nitrogen pollution provides a proxy for the optimal nitrogen rate for society and provides guidance for fertilizer and nitrogen policies^13,44^.

The calculated range of nitrogen rates delivering a high net positive financial is reduced by: (1) using the ST nitrogen response, (2) using the LT nitrogen response and (3) including external costs. The corresponding ranges of nitrogen rates for price levels in the Netherlands, using ST and LT nitrogen response curves for wheat in rotation in the Broadbalk LTE, are: (1) 219 kgN ha^−1^ (range, 14–233 kgN ha^−1^), (2) decreasing to 157 kgN ha^−1^ (range, 61–218 kgN ha^−1^) and (3) decreasing further to 90 kgN ha^−1^ (range, 45–135 kgN ha^−1^) (Supplementary Note 10). In other words, including the external costs will reduce the optimal nitrogen level by 40% in this case.

**Fig. 6:**
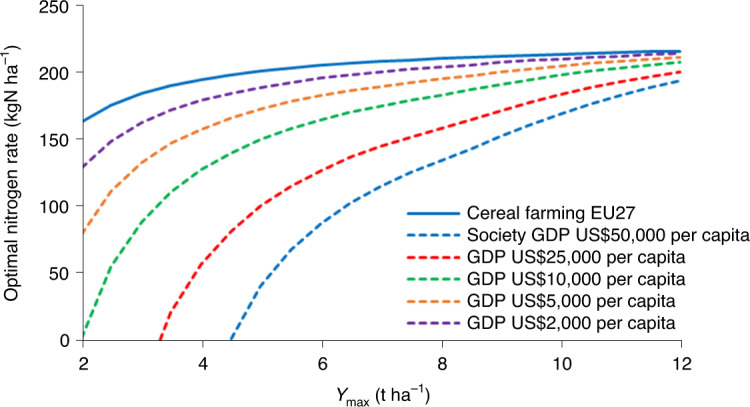
Societal optimal nitrogen rates for global cereals. Increase in societal optimal nitrogen rates for global cereals with increasing yield potential (*Y*_max_; from 2 to 12 t ha^−1^) and with decreasing GDP (from US$50,000 to US$2,000 per capita) using the generic LT response of yield to nitrogen availability (equation (2)), a ‘farm gate to food plate’ price ratio of 1 and a decrease in the marginal cost of nitrogen pollution with GDP. For comparison, the optimal nitrogen rate for farming in the European Union is shown. Source data

For global cereals, we varied *Y*_max_ from 2 to 12 t ha^−1^ and GDP from US$2,000 to US$50,000 per capita (Fig. 6). The marginal costs of crop production and nitrogen pollution for countries are estimated using an income (GDP) elasticity of 0.85 (Supplementary Note 11). The optimum nitrogen rate for farming profits increases with the maximum attainable yield. This increase is independent of GDP but depends on the prices of grain and fertilizer. In many regions with high market access, world market prices apply, but in some regions of the developing world, production and consumption of cereals are more controlled by local markets; for example in Kenya prices for wheat and nitrogen fertilizer are up to 1.5–2 times higher than in Europe and North America^45^. For economic analysis at a society scale we considered that the virtual price per kilogram of cereal on the ‘food plate’ is higher than at the farm gate. For this we ran scenarios with price ratios of 1 and 3. We did not account for feedback effects of reduced cereal supply on prices or for subsidies on cereal and fertilizer, although these are present in many regions; for example, nitrogen fertilizer subsidies are up to 70% of the world market price in India^46^.

The optimal nitrogen rate for society (SONR) is lower than the optimal rate for farming, and the difference between these values increases strongly with GDP. At an *Y*_max_ of 6 t ha^−1^ and a GDP of US$50,000 per capita, the optimal values for farm and society are 207 and 88 kgN ha^−1^, respectively, and for a GDP of US$2,000 capita the corresponding values are 209 and 197 kgN ha^−1^, respectively. However, *Y*_max_ also tends to increase with GDP because of better access to technology and high-yielding cultivars and better farm management. The lower the GDP, the lower the difference between the optimal nitrogen rates for farming and society. The higher the potential yield, the smaller the difference between the optimal nitrogen rates for countries.

### Assessing the safe operating space for nitrogen fertilizer application

For regional farm nutrient management and national environmental management, knowledge of the safe operating space for nitrogen application is more relevant than knowledge of the optimum point values. The concept of ‘safe space’ of nitrogen application^47^ can be defined as the nitrogen range where yields are high, pollution is low and where net economic benefits for both farming and society are in balance^48^. This ‘safe space’ of nitrogen rates is illustrated for three GDP levels, US$50,000 per capita (typical for North America and the northwestern European Union), US$25,000 per capita (typical for the central and southern European Union), US$10,000 per capita (typical for Eastern Europe and South America) and for two ‘food plate to farm gate’ cereal price ratios.

**Fig. 7:**
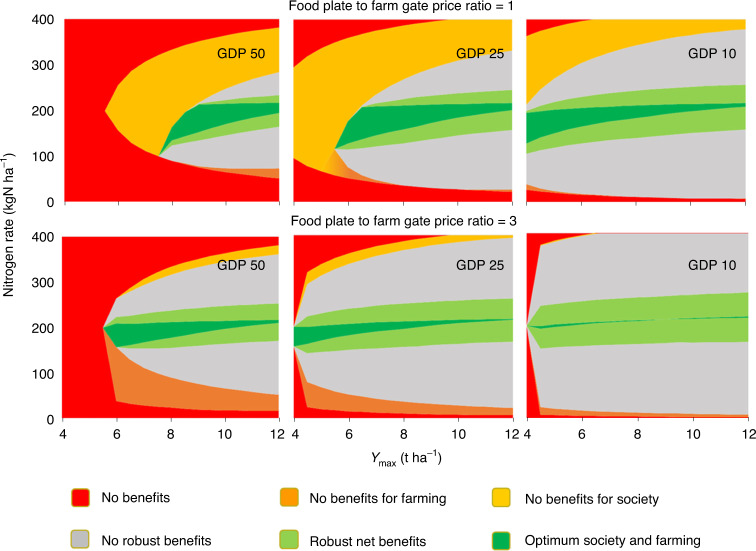
Classification of LT economic benefits for farming and society of adding nitrogen fertilizer to rain-fed wheat with increasing attainable yield (*Y*_max_), for three levels of GDP and two ‘food plate to farm gate’ price ratios of grain. Nitrogen application as calcium ammonium nitrate equivalents at a fertilizer price of US$1 kgN^−1^ and assuming no other nitrogen inputs than background (SN) of 25 kgN ha^−1^. Grain price is US$0.2 kg^−1^. Damage cost per kilogram of nitrogen surplus increases with GDP (by US$4, US$10 and US$20 kg^−1^ for GDPs of US$10,000 (right panels), US$25,000 (middle panels) and US$50,000 (left panels) per capita, respectively), as does fixed farm cost (US$185, US$480 and US$980 ha^−1^, respectively). The ‘robust’ range of nitrogen rates is set at 0.25 of the range with net benefits.

The minimum *Y*_max_ allowing ‘safe’ (beneficial) application of nitrogen fertilizer decreases with increasing GDP (Fig. 7). The safe range of nitrogen rates, with robust net benefits for both farming and society, is fairly constant, increasing only slightly with GDP. However, the optimum range with high benefits for both farming and society strongly decreases with increasing GDP and price ratio. With increasing GDP, society is increasingly willing to pay to prevent the impacts of nitrogen pollution and the fixed farm costs per hectare increase (for both, we use a GDP elasticity of 0.85).

When food prices are high—for example, in the event of food shortage or food hedonism—people tend to accept more nitrogen pollution. Our welfare analysis is simple and we did not consider feedback effects of regional changes in cereal production on prices of grain and land. If SONR were to be applied globally, global yield supply would not change very much as lower nitrogen rates and yields in developed regions would be compensated by increases in developing regions. Furthermore, the farm gate price of raw cereals contributes about 10% to the price of cereal food products in the United States and the European Union (Supplementary Note 13). Therefore the LT effects of a modest change in global cereal supply on cereal prices will be modest, in spite of the relative inelasticity of cereal demand^49^.

EONR is more sensitive to the shape of the LT nitrogen response curve than to the price of cereal. SONR is most sensitive to *Y*_max_ for high *Y*_max_ (8 t ha^−1^) and high GDP (US$50,000 per capita), while for medium *Y*_max_ (4 t ha^−1^) and medium GDP (US$10,000 per capita) it is also sensitive to GDP. Uncertainty in EONR is also dominated by uncertainty in the shape of the LT nitrogen response curve, while uncertainty in *Y*_max_ contributes most to uncertainty in SONR (Supplementary Note 12). Uncertainties in SONR (6–8%) are higher than for yield (1–2%) and do not affect our welfare analysis, which is intended to illustrate the direction and approximate size of effects of *Y*_max_ and *N*_rate_ on SONR, focusing on high- and middle-income countries. For low-income countries (GDP < US$1,000 per capita) SONR converges to EONR (Figs. 6 and 7), but our welfare analysis is less applicable here due to the lack of valuation data for nitrogen pollution.

For the Netherlands, which has a GDP close to US$50,000 per capita, current *Y*_max_ ranges between 8 and 10 t ha^−1^, while current (mineral) fertilizer equivalent nitrogen rates range between 150 and 200 kgN ha^−1^ (Supplementary Note 12). This current range overlaps with the safe space, but current nitrogen rates exceed the optimal nitrogen rates for society by 15–30 kgN ha^−1^. The same conclusion applies to France (GDP = US$35,000 per capita), while for Romania with a GDP of US$9,000 per capita, current nitrogen rates of between 50 and 100 kgN ha^−1^ are about 50 kgN ha^−1^ below the safe space (Supplementary Note 12). Transposing 30 kgN on a hectare under wheat from the Netherlands to Romania would increase the societal benefit, without yield loss. For countries with a GDP < US$5,000 per capita, optimal nitrogen rates for farming and society converge. For China (GDP = US$3,200 per capita; rainfed wheat *Y*_max_ = 6–9 t ha^−1^), current nitrogen rates of 200–300 kgN ha^−1^ exceed the farming optimum of 200–225 kgN ha^−1^. In India (GDP = US$1,000 per capita, *Y*_max_ = 3–6 t ha^−1^) current nitrogen rates are around 100 kgN ha^−1^ which is about half of EONR and SONR, while urea fertilizer is subsidized. In Kenya (GDP = US$1,200 per capita, *Y*_max_ = 3–6 t ha^−1^) current rates are around 50 kgN ha^−1^, and about 30% of EONR and SONR.

Gaps between current nitrogen rates and the safe operating space may appear quite modest but will tend to increase in the future for different reasons. In the European Union this gap will increase due to stricter environmental nitrogen policies and ambitions for extensification and nature inclusiveness, which both will tend to reduce yields per hectare. In Kenya and India, as examples of developing regions, increasing GDP will increase willingness to pay to reduce pollution and therefore increase marginal external nitrogen costs per kilogram of nitrogen surplus. The shape of the safe range of *Y*_max_–*N*_rate_ combinations illustrates that for nitrogen rates above 150 kgN ha^−1^ development and access to higher-yielding cultivars is a better strategy for more sustainable agriculture than strategies to increase application of synthetic nitrogen.

## Conclusions

Based on 25 LT field experiments with maize, wheat or barley we found a generic relationship expressing the responses of both cereal yield and agronomic nitrogen efficiency to nitrogen application rate. The relationship is applicable globally and for a wide range of conditions. It is very different from the short-term responses that are commonly used. The generic relationship applies for *Y*_max_ in the range 2–16 t ha^−1^ as in the underlying observations. A *Y*_max_ lower than 2 t ha^−1^ indicates strong growth limitation by water or other factors, hampering normal crop development and response to nitrogen fertilizer, while a *Y*_max_ above 16 t ha^−1^ may apply to special cultivars or management practices. The LT trials used in this study do not include use of manure, but our generic curve is probably also applicable for organic fertilizers using replacement values for manure nitrogen by assuming an observed LT fertilizer replacement value of 1 (ref. ^50^). While initial results for lowland rice are promising, more LTEs for other regions are needed for global applicability. Global application can be improved by compilation and analysis of observations of SN and *Y*_0_. The mere existence of these curves may point to universal principles of plant metabolism and scalable mass relationships as found by West et al.^51^. Application of our generic response curve has important implications for optimal nitrogen rates for agriculture and society as needed to ensure farm income, food sufficiency and sustainable agrofood systems. As an illustration for agriculture, we recalculated the global maize production reported by Mueller et al.^52^ using our generic response curve. This reduced the global maize yield by about 120 Mt (20%) for the same global amount of nitrogen fertilizer use. This implies that the LT nitrogen fertilizer needed to achieve a target maize yield (here, for 2000) is higher by 6 MtN (40%) than that based on the ST response. This indicates that current global maize production relies on unsustainable net soil nitrogen depletion (Supplementary Note 12). As an illustration for society, we find that the inclusion of external costs of nitrogen fertilizer use in intensive, high-income countries reduces optimum nitrogen rates for cereals by almost 25% compared with current optimum rates for farm economy. Using our generic response function, the nitrogen rate that safeguards robust farm returns, regional food sufficiency and more acceptable nitrogen pollution levels varies greatly across the world. ‘Too little’ regions need more nitrogen fertilizer to jump-start crop yields and replenish nitrogen-depleted soils, whereas ‘too much’ regions with high GDP need to reduce nitrogen fertilizer input^3,48^. Policies to implement SONR globally will both reduce and redistribute global use of synthetic nitrogen and may have important consequences for the current food system, for example, changes of land use (and land prices and rent), choice of cultivars and rotations to increase NUE, and possibly higher food prices and farm gate prices to compensate for lower yields per hectare. The route towards SONR needs to be evaluated against other options to reconcile nitrogen pollution and food sufficiency, both regarding farm nitrogen management (not only the nitrogen rate but also precision nitrogen timing and placement and use of fertilizer products with higher nitrogen efficiency) and nitrogen policies (for example, nitrogen regulation versus nitrogen taxation). Our long-term nonlinear response of yields to changed input of synthetic fertilizer could be incorporated in computable general equilibrium models to improve projection on how markets respond to changes in fertilizer regimes or policies. Our calculation of the external cost of nitrogen pollution could be used to define nitrogen pollution taxes as part of policies to offset the regressive distributional effects of internalizing external effects. Implementation of more inclusive nitrogen policies that account for environmental costs comes with the risk of increased land demand and will change the spatial allocation of cereal production and regional import/export of cereals (for example, in Europe^53^). These risks can be mitigated by additional policies to reduce food waste and change food choices^54^ to prevent export of nitrogen polluting agricultural activities from high- to low-GDP countries^44^. Dealing with nitrogen problems in global agriculture requires a holistic nitrogen^55^ and food system approach, balancing risks and opportunities for changes in land use and resource security for agriculture, rural livelihoods and dietary choice^56^.

## Methods

### Broadbalk wheat experiments

We used results from the Broadbalk LTE at Rothamsted Research to construct LT nitrogen response curves for winter wheat in rotation and continuous wheat^20^. Results apply to trials at the Broadbalk site for the period 1985–2018, where only mineral fertilizer was used and phosphorus, potassium, magnesium and pesticides were adequately supplied. Mineral nitrogen application levels were 0, 48, 96, 144, 192, 240 and 288 kgN ha^−1^. Phosphorus fertilizer rates were 0 and 35 kgP ha^−1^, and the potassium rate was fixed at 90 kgK ha^−1^. At low nitrogen levels, grain yields for 35 kgP ha^−1^ were somewhat higher but not significantly so (95% confidence interval; Supplementary Fig. 2). Therefore, the results for 0 and 35 kgP ha^−1^ were pooled for the analysis of nitrogen response. The zero-fertilizer plots further offer insight into effects of changing air pollution and climate on crop yield over the past 150 years. Interestingly, the yields of winter wheat in the zero-fertilizer plots have varied considerably over the past 150 years, between 0.5 and 1.5 t ha^−1^ but showed no net increase or decrease. However, yields of optimally fertilized plots in crop rotation showed a yield increase by a factor of 5 for winter wheat.

The wheat varieties used were Brimstone (1985–1990), Apollo (1991–1995), Hereward (1996–2012) and Crusoe (2013–2018); data for 2015 were excluded from the analysis as spring wheat was sown that year, due to very wet autumn weather conditions preventing the usual sowing of winter wheat. For wheat in rotation preceding crops were mostly potato or forage maize.

Data for the ST nitrogen response of winter wheat in rotation are based on 15 trials for commercial crops in different parts of England representative of the main arable areas in 1994–1998^25^. Mineral nitrogen application levels were 0, 80, 120, 160, 2000, 240 and 280 kgN ha^−1^, that is, in the same range as in the Broadbalk experiment (and without explicit information on the rotation).

### Long-term field trials

The ST–LT distinction is to some extent arbitrary. The most relevant consideration is that soil nitrogen should be sufficiently close to steady state that the yield response to a change in fertilizer input can be quantified, which could also be formulated as that response curves have adequate curvature to determine SN and *Y*_max_. For Europe we used 11 LTEs for winter wheat and two for barley^57^. We used eight LTEs for wheat in South Asia and found two for China. For maize we found three LTEs for the United States (Supplementary Note 4) and two for China. This added up to a total of 27 LTEs (Supplementary Note 1). These 27 LTEs cover a wide range of soils, climates, cultivars, fertilizer types and management regimes. In all trials, other nutrients were not deficient. Two of the 27 trials were discarded, a wheat trial in Bologna, Italy with SN = 103 kgN ha^−1^ and one in Punjab, Pakistan with SN = 389 kgN ha^−1^). We considered SN values exceeding 100 kgN ha^−1^ as an indication that soil nitrogen was too far from steady state.

Soil types were mostly loam and clay soils. Climates were temperate (mean annual temperature, 9 °C; annual precipitation, 700 mm), continental (mean monthly minimum temperature, −10 °C; maximum temperature, 30 °C; annual precipitation, 450–800 mm) and tropical (mean monthly maximum temperature, 35–45 °C; minimum temperature, 7–14 °C; annual precipitation, 1,500–1,800 mm). Fertilizer types in Europe and the United States were mostly ammonium nitrate and in Asia mostly urea with sometimes part of the nitrogen fertilization from diammonium phosphate. Fertilizers were applied as one to three dressings, but the number of dressings probably had little effect on nitrogen response^58^.

### Scaling procedure for nitrogen response

Experimental nitrogen response data were scaled and fitted by second-order polynomials, assuming scaled observations for multiple sites were uncorrelated. For scaling, two transformations were applied,

1. *y* axis: transformation of observed absolute site yield to yield index by dividing by *Y*_max_. The *y* index ranges from 0 to 1.
2. *x* axis: transformation of the rate of added nitrogen in mineral fertilizer to total nitrogen input rate, including nitrogen inputs from nitrogen deposition, biological nitrogen fixation and net soil nitrogen mineralization. The sum of nitrogen inputs from these other nitrogen sources was approximated by the *x* intercept of the second-order polynomial fit (Supplementary Fig. I3).

The assumption that the 119 scaled observations for the 25 LTEs are uncorrelated while in fact being stratified was tested by comparing the fitted second-order polynomial on the total dataset (equation (2)) to the 25 fitted polynomials for the individual sites. Equation (2) proved to be identical to the median regression line after sorting the 25 regressions by *Y*_r_ for *N*_av_ = 100 kgN ha^−1^.

### NUE and nitrogen loss for wheat in the Broadbalk LTE

While nitrogen fertilizer input is generally the main driver for increasing cereal yields, overfertilization and poor timing and placement of fertilizer is a major cause of nitrogen pollution^48,59^. Data on nitrogen content in grain and straw were only available for the Broadbalk LTE and not for the 25 global LTEs. The NUE in the Broadbalk LTE is expected to be higher than for most global practices in view of better management and assumed near steady state. The nitrogen content in grain for wheat in rotation in the Broadbalk LTE is about 1.5% up to a total nitrogen input of 100 kgN ha^−1^, increasing linearly up to 300 kgN ha^−1^ (Supplementary Note 9). A linear model of *N*% with *Y*_max_ and *N*_av_ was fitted to observations between 1985 and 2016, in which *Y*_max_ for a given year varied between 6.5 and 12.9 t ha^−1^.

4 $$\begin{array}{rcl}N\% & = & 1.873 + (3.26 \times 10^{-3} \times N_{\mathrm{av}})\\ && -(6.20 \times 10^{-2} \times Y_{\mathrm{max}})\quad (R^2 = 0.743,N = 224)\end{array}$$

where *N*% is the nitrogen percentage in grain. The nitrogen dilution effect with increasing *Y*_max_ and the nitrogen enrichment with *N*_av_ are both highly significant (>99.9%) and relevant, but the statistical significance of the effect of *N*_av_ (*t-*statistic, 24.5) is larger than that of *Y*_max_ (*t-*statistic, −5.6). In view of the LT nature of the Broadbalk LTEs and the application of nitrogen rate scaling, equation (4) has global applicability for wheat cultivation.

While grain yields level off with nitrogen input, the LT nitrogen removal in grain increases from a zero intercept almost proportionally with nitrogen input up to 250 kgN ha^−1^, which is in line with previous findings for arable systems on a country scale^59^ (Supplementary Fig. 22b). Nitrogen surpluses and the subsequent risk of nitrate leaching start at a total nitrogen availability of 180 kgN ha^−1^, which for Broadbalk corresponds to a nitrogen fertilizer rate of 150 kgN ha^−1^ (Supplementary Fig. 22c). The mean NUE for the 32 years of observation increases from 40% at 50 kgN ha^−1^ to a peak at about 80% at a total nitrogen availability of 150 kgN ha^−1^ and gradually decreases again to 60%. An *N*_av_ of 150 kgN ha^−1^ in the Broadbalk LTE corresponds to a nitrogen fertilizer rate of 120 kgN ha^−1^ (Supplementary Fig. 22d). The observed initial increase in NUE may be caused by increased tillering and root development with addition of nitrogen fertilizer, promoting efficient uptake of available nitrogen and internal nitrogen allocation (sink strength governed by tiller and grain numbers).

### Calculation procedure of optimal nitrogen rates

In this paper we combine approaches from microeconomics (production economics, individual optimizing agents), environmental economics (price on externalities) and macroeconomics (regional to global agriculture, society, welfare). Our basic macroeconomic analysis considers differences in prices and costs around the world, but does not account for multiple interacting markets and their effects on cereal prices when cereal supply changes. We calculate two economic optima for nitrogen application: for cereal farming and for society. In both cases, the optima depend on the slope of the nitrogen response curves (Supplementary Figs. 23 and 24). The net benefit function *B* is:

5 $$B = Y \times P_y-(N_{\mathrm{rate}} \times {\mathrm{PN}})-(C_{\mathrm{fixed}})-({\mathrm{CN}}_{\mathrm{pollut}})$$

where *P*_*y*_ is the crop price (US$ kg^−1^), PN is the fertilizer price (US$ kgN^−1^), *C*_fixed_ is the cost of seed, tillage, harvest and other inputs, and CN_pollut_ the external cost of nitrogen pollution. For farming, CN_pollut_ is not considered. By considering prices for both farming and welfare, equation (5) combines production economics and environmental economics because it addresses both producers and consumers. How both agents will respond to these prices to maximize their utility will depend on policy context. The negative externalities can be implemented as a tax on nitrogen and in that case the two optimal nitrogen applications would be the same. Alternative communication of nitrogen issues and design of nitrogen policies can make farmers beneficiaries of reduced nitrogen pollution and consumers virtual payers of improved nitrogen fertilizer management (for example, by food labelling and nitrogen footprinting, http://www.n-print.org/).

The economic optimum for farming can be determined from the following equation:

6 $${\mathrm{d}}Y/{\mathrm{d}}N_{\mathrm{av}} \times P_y - {\mathrm{PN}} = 0$$

where d*Y/*d*N*_av_ is the first derivative of the unscaled nitrogen response function as derived from equations (1) or (2), using case-specific values of *Y*_max_ and SN. For the calculation of the optimum nitrogen rates the quadratic global relation between cereal yield and *N*_*av*_ (equation (2)) is substituted in equation (6). The minimum value of *N*_av_ is calculated by solving equation (5) for *B* = 0. This minimum nitrogen rate depends strongly on *C*_fixed_; *C*_fixed_ increases per unit of yield with decreasing yield and provides the penalty for farmers when decreasing nitrogen input too much. The resulting equation for *B* can also be expressed as a second-order polynomial of nitrogen rate, and optima and cross-points simply follow from standard calculus for solution of quadratic functions.

The calculation of the optimum nitrogen rate for society also accounts for the increase in nitrogen pollution with increasing nitrogen input:

7 $${\mathrm{d}}Y/{\mathrm{d}}N_{\mathrm{av}} \times P_y-(PN)-({\mathrm{d}}C_{\mathrm{fixed}}/{\mathrm{d}}N_{\mathrm{av}})-({\mathrm{d}}CN_{{\mathrm{pollut}}_i}/{\mathrm{d}}N_{\mathrm{av}}) = 0$$

*P*_*y*_ is not the farm gate price of cereals as such, but the price equivalent as paid by those who are bearing the cost of nitrogen pollution. This virtual ‘food plate’ price of rough grain could be higher than the farm gate price and accounts for value creation in food processing after correction for assignable costs (for example, labour and energy for milling and baking) and reflects the cost of shareholder dividends, risk insurances or market imperfections in the cereal supply chain. Because this price is uncertain, we solved equation (7) for *P*_*y*_ equal to the farm gate price and three times this value. A ratio of 3 is consistent with the relative increase of gross added value of all agricultural commodities in food processing in the European Union (ratio of 2) and the United States (ratio of 3). A ratio of 3 is also consistent with a ratio of 1.2–2.6 based on the farm gate price of bread wheat in northwest Europe (US$0.25 kg^−1^) and in bread (US$0.3–0.6 kg^−1^) (Supplementary Note 13). To include the cost of the various impacts of nitrogen pollution, $$N_{{\mathrm{pollut}}_i}$$ (where subscript *i* refers to various nitrogen pollutants, for example, NO_3_, NH_3_, N_2_O) can be expressed in monetary units by multiplying the pollutant flux by their respective unit damage costs (US$ kgN^−1^)^11^. Pollutant fluxes are estimated as fractions of nitrogen inputs or nitrogen fluxes. Here we approximated *N*_pollut_ by nitrogen surplus and a lumped unit damage cost per kilogram of nitrogen surplus (Supporting Note 10). Nitrogen surplus was calculated as:

8 $$N_{\mathrm{surplus}} = {N_{\mathrm{av}}}-{N_{\mathrm{removal}}}$$

where *N*_removal_ is the nitrogen removed by the crop, calculated as: *Y* × *N*%/100 (where *N*% is calculated from equation (4)). The resulting relations between *B* and *N*_av_ can be expressed as third-order polynomials and optima and cross-points were determined using the SOLVER function in Excel.

To calculate the optimal values and safe ranges of *Y*_max_ and *N*_av_ we used a ceteris paribus approach, and did not take into account consequential effects of changes in cereal production on *P*_*y*_ or *C*_fixed_, the latter caused, for example, by effects of land prices and rent. This would require application of computable general equilibrium models to model global supply, demand and trade of cereals, and would involve many assumptions, for example, regarding changes in relative use of cereals for food, feed and fuel. Our simple economic approach is to demonstrate the effect of using LT instead of ST response curves and considering the social cost of nitrogen surplus and the safe operating range of *N*_av_.

### Calculation of nitrogen pollution cost and optimal nitrogen rates for countries

The current nitrogen rate for wheat on sandy to loamy soil in the Netherlands is 165 kgN ha^−1^ and 60% of nitrogen is applied as manure^60^. For the calculation of total allowable nitrogen rate, it is assumed that 1 kg of manure nitrogen has a fertilizer equivalence of 60% of 1 kgN applied as calcium ammonium nitrate, and manure nitrogen input for arable systems is limited to 170 kgN ha^−1^ (ref. ^60^). Current Dutch environmental legislation limits the total fertilizer equivalent nitrogen rate from synthetic fertilizer and manure for winter wheat to 165 kgN ha^−1^ for sandy soils and to 190 kgN ha^−1^ for loess. These rates are economically suboptimal for farming, irrespective of the use of ST or LT curves. However, winter wheat is grown in rotation which provides residual soil nitrogen for the subsequent wheat crop. For the fixed cost of (contracted) labour for planting, tillage, crop management and harvest for wheat cultivation in the Netherlands we used a value of US$680 ha^−1^ yr^−1^, and for other inputs such as phosphorus, potassium, pesticides and energy we used a value of US$430 ha^−1^ yr^−1^ (Supplementary Note 10).

For quantification of the cost of nitrogen pollution for other countries we used a GDP-dependent cost per unit of nitrogen surplus (UC), derived from results for 27 EU countries^3,44^ (Supplementary Note 11):

9 $${\mathrm{UC}} = 0.3412 \times {\mathrm{GDP}}^{1.0362}\quad (R^2 = 0.6673)$$

In the EU27 dataset the mean national GDP between 2010 and 2014 ranged from US$7,000 per capita in Bulgaria to US$59,000 per capita in Denmark (excluding the US$108,000 per capita for the very small country of Luxembourg), the nitrogen surplus in 2008 between 23 kgN ha^−1^ of used agricultural land in Romania and 176 kgN ha^−1^ in the Netherlands, and the UC ranged from US$2 kgN^−1^ (Bulgaria) to US$43 kgN^−1^ (Denmark). The GDP effect reflects increasing willingness to pay to prevent nitrogen pollution, making GDP a major determinant for external costs of nitrogen pollution of waters in Europe^13^, but less so for the United States and the rest of the world^61^.

In the Netherlands and many other areas with intensive use of manure or urea fertilizer, ammonia losses are mainly associated with manure, and the impacts of ammonia-containing aerosols on human health dominate nitrogen pollution cost^3,44^. Globally, ammonia losses depend on the choice between ammonia (often urea) or nitrate-type fertilizer (often calcium ammonium nitrate), the use of manure and the application of low-emission techniques. All these factors will change considerably in the near future due to improved management to increase cost-efficiency and as a consequence of environmental regulation.

### Reporting summary

Further information on research design is available in the Nature Research Reporting Summary linked to this article.

### Supplementary information

Supplementary Information Supplementary Notes 1–14 containing additional information regarding data used, methods and discussion in the main paper and supporting analyses and additional results in tables and figures.

Reporting Summary.

### Source data

Source Data Fig. 1 Source Data Fig. 1.

Source Data Fig. 3 Source Data Fig. 3.

Source Data Fig. 4 Source Data Fig. 4 and additional data from 25 long-term field trials and results of regression.

Source Data Fig. 5 Source Data Fig. 5.

Source Data Fig. 6 Source Data Fig. 6.

## Data Availability

Summaries of nitrogen response data for the Broadbalk winter wheat experiments at Rothamsted Research and for global cereals are provided in the Supplementary Information; details are available upon reasonable request. Selections of original observations for the Broadbalk experiment are available via the electronic Rothamsted archive (http://www.era.rothamsted.ac.uk/). Source data are provided with this paper.
