# Author Correction: Establishing long-term nitrogen response of global cereals to assess sustainable fertilizer rates

**DOI:** 10.1038/s43016-023-00900-z

**Published:** 2023-11-20

**Authors:** Hans J. M. van Grinsven, Peter Ebanyat, Margaret Glendining, Baojing Gu, Renske Hijbeek, Shu Kee Lam, Luis Lassaletta, Nathaniel D. Mueller, Felipe S. Pacheco, Miguel Quemada, Tom W. Bruulsema, Brian H. Jacobsen, Hein F. M. ten Berge

**Affiliations:** 1 Department of Water, Agriculture and Food, PBL Netherlands Environmental Assessment Agency, The Hague, Netherlands; 2 Department of Agricultural Production, School of Agricultural Sciences, Makerere University, Kampala, Uganda; 3 Department of Computational and Analytical Sciences (CAS), Rothamsted Research, Harpenden, UK; 4 College of Environmental and Resource Sciences, Zhejiang University, Hangzhou, PR China; 5 Plant Production Systems Group, Wageningen University, Wageningen, Netherlands; 6 School of Agriculture and Food, Faculty of Veterinary and Agricultural Sciences, The University of Melbourne, Melbourne, Victoria Australia; 7 Department Agricultural Production/CEIGRAM, Universidad Politécnica de Madrid, Madrid, Spain; 8 Department of Ecosystem Science and Sustainability, Department of Soil and Crop Sciences, Colorado State University, Fort Collins, CO USA; 9 National Institute for Space Research (INPE), Earth System Science Center, São José dos Campos, Brazil; 10 Plant Nutrition Canada, Guelph, Ontario Canada; 11 University of Copenhagen, Department of Food and Resource Economics (IFRO), Copenhagen, Denmark; 12 Wageningen Plant Research, Wageningen, Netherlands

**Keywords:** Environmental sciences, Environmental social sciences, Agriculture

Correction to: *Nature Food* 10.1038/s43016-021-00447-x, published online 31 January 2022.

This paper was originally published under a standard Springer Nature license (© The Author(s), under exclusive licence to Springer Nature Limited). It is now available as an open-access paper under a Creative Commons Attribution 4.0 International license, © The Author(s). The error has been corrected in the online version of the article.

