# Supplementary material for: Establishing long-term nitrogen response of global cereals to assess sustainable fertilizer rates
Source: Nat Food. 2022 Jan 31;3(2):122–32. doi: 10.1038/s43016-021-00447-x (PMC10661743; doi:10.1038/s43016-021-00447-x)
Supplement: Supplementary file 1 — Supplementary Notes 1–14 containing additional information regarding data used, methods and discussion in the main paper and supporting analyses and additional results in tables and figures. [file 43016_2021_447_MOESM1_ESM.pdf]

nature portfolio

https://doi.org/10.1038/s43016-021-00447-x

Supplementary information

Establishing long-term nitrogen response of global cereals to assess sustainable fertilizer rates

In the format provided by the authors and unedited

nature portfolio

https://doi.org/10.1038/s43016-021-00447-x

Supplementary information

Establishing long-term nitrogen response of global cereals to assess sustainable fertilizer rates

In the format provided by the authors and unedited

Supplementary Information to:

Establishing long-term nitrogen response of global cereals to assess sustainable fertilizer rates

Hans JM van Grinsven, Peter Ebanyat, Margaret Glendining, Baojing Gu, Renske Hijbeek, Shu Kee Lam, Luis Lassaletta, Nathaniel D Mueller, Felipe Pacheco, Miguel Quemada, Brian H. Jacobsen, Hein FM ten Berge

Contents[1]

Note 1 Characteristics of used long term N response studies 2

Note 2 Broadbalk wheat experiment, Rothamsted Research, UK 13

Note 3 Procedures for data scaling and curve fitting 15

Note 4 Long term N response of maize in the USA 20

Note 5 Statistics and cross validation of scaled yield response curve for global cereals 23

Note 6 Validation of generic N response at country scale for Europe and Africa 24

Note 7 Predicting long-term N response from short-term field trials 29

Note 8 Generic long-term N-response curve for paddy irrigated rice in Asia 33

Note 9 Crop and soil nitrogen response to total N availability for Winter wheat at Broadbalk and implications for *NUE* 35

Note 10 Optimal N fertilizer rates for farm and society; concept and illustrative results for the Netherlands 38

Note 11 External cost of N surplus in the European Union 40

Note 12 N fertilizer benefits and optimal N rates for farm and society; examples and uncertainty analysis 43

Note 13 Farm gate and food plate prices of cereals 47

Note 14 Implications of using LT N response for global maize cultivation 51

[1] Numbered references can be found in main article, additional references are inserted in text

Note 1 Characteristics of used long term N response studies

Experiment	Region	Crop	Type	Start and used Period	Key Reference
Winter wheat					
Broadbalk	United Kingdom, Rothamsted, Chromic or Vertic Luvisol. Mean rainfall 696 mm. Mean Temp 1878-1988: 9 °C; 1989-2010: 10 °C	Two data sets: In rotation with other arable crops and continuous wheat	Field, 7 N rates; N as ammonium nitrate. One dressing. 0-35kgP/ha; 90 kgK/ha; 12 kgMg/ha	1843; 1985-2018	Johnston et al., 2018; Macdonald 2018
Müncheberg	Germany. Leptic Podzol. Light loamy sand. Annual rainfall 511 mm, mean annual temperature 8.4°C.	In rotation with root crops	Field, 5 N rates. 30 kg/ha P_2O_5; 100 kg/ha K_2O	1962; 1984-2002	Rogasik et al., 2001; Hijbeek et al., 2017
Limburgerhof	Germany. Loamy sand. Pseudogley-Paternia. Annual rainfall 545 mm, mean annual temperature 10°C.	In rotation with maize, barley and turnip	Field, 5 N rates. 60 kg/ha P_2O_5; 120 kg/ha K_2O	1987; 1987-1994	Lang et al., 1995
Oldenburg	Germany. Light loamy sand. Annual rainfall 728 mm, mean annual temperature 8.4°C.	In rotation with barley and sugar beet.	Field, 5 N rates. Basic fertilization with P, K and Mg according to recommendation	1984; 1985-1993	Klasink and Steffens 1995

Rauischholzhausen	Germany. Loess loam. Annual rainfall 583 mm, mean annual temperature 8°C.	In rotation with barley and sugar beet.	Field, 5 N rates. 40 kg P/ha, 140 kg K/ha	1984; 1985-1990	Von Boguslawski, 1995
Speyer	Germany, **Current** climate: Annual rainfall 671 mm, mean annual temperature 10.3°C.	In rotation with barley and sugar beet.	Field, 5 N rates, 3 dressings. Fertilization with P and K according to recommendation	1984; 1994-1999	Bischoff and Emmerling, 2001
Spröda	Germany	In rotation	Field, 5 N rates	1966; 1999-2010	Albert and Grunert 2013; Körschens et al. 2014
Grabow	Poland, sandy loam, Annual rainfall 294 mm, mean annual temperature 13°C.	In rotation	Field, 4 N rates	1980; 2003-2016	Rutkowska and Skowron, 2020
India, Pakistan, Bangla Desh	South Asia		Nine sites, 16 field trials; probably urea	1982-2008	Jat et al., 2014
Laiyang, Shandong	China	Maize - wheat rotation	Field, 3 N rates. Urea. Two dressings; hole and furrow application	1978-2013	Personal communication
Lossa, Konni	Niger. Psammentic Paleustalf sand	Maize, millet, sorghum,	Field, 5 N rates, Urea. Half of N at planting, half 6 weeks after planting.<br>38 kgP/ha as triple superphosphate and 100 kg K/ha as potassium chloride.<br>Not a LTE.	1997-1998	Pandey et al., 2001

Chikwawa	Malawi	Irrigated maize-rice two crop system	Field, 4 N rates, not an LTE, 10-30 kgP/ha	2007	Fandika et al., 2008
Winter Barley					
Oldenburg	Germany	In rotation	Field, 5 N rates	1984; 1985-1993	Klasink and Steffens 1995
Speyer	Germany	In rotation	Field, 5 N rates	1984; 1994-1999	Bischoff 1995
Maize					
Wisconsin, Lancaster (Univ. of Wisc. Exp station)	USA, Rozetta silt loam. Mean temp 7.5; monthly low -13, max 27 °C. rainfall 900 mm	In rotation and continuous	Field, 4 N rates, 7 rotations, 2 replicates; ammonium nitrate; broadcast	1968, 1990-2004	Stanger et al., 2006; Vanotti and Bundy 1994.
Kansas, Tribune Unit, Southwest Research-Extension Center	USA, Tribune, silt loam. Rainfall 443 mm, mean temp 11.2; mean month low -9; month max 33	Irrigated continuous	Field, 6 N rates, ammonium nitrate. Furrow irrigation until 2000, from then sprinkler	1961; 1997-2006	Schlegel et al., 2017
Iowa, Story City	USA. Kossuth silty clay loam and Ottosen clay loam. Rain 770 (906) mm. Mean temp 10.5. Max/min temp 30/-10 C	Maize-Soybean	Field + Model, 7 N rates, 3 dressings; liquid urea ammonium nitrate (UAN)	1996-2005	Thorp et al., 2007
Changping	China. Calcareous alluvial fluvo-aquic soil with a	Irrigated continuous	Field, 3 N rates; urea, 60% of which was applied as base fertilizer and the rest was	1984, 2011-2012	Wen et al., 2016

	silty loam texture. Mean temp. 13 and rainfall 699 mm,		top-dressed at jointing stage. Split-plot, factorial design; 12 different combinations of N and/or P with or without chicken manure (M) as treatments in 4 replications		
Laiyang, Shandong	China	Maize wheat rotation	Field, 3 N rates. Urea. Two dressings; hole and furrow application	1978-2013	Personal communication Boajing Gu
Lossa, Konni	Niger. Psammentic Paleustalf sand	Maize, millet, sorghum,	Field, 5 N rates, Urea. Half of N at planting, half 6 weeks after planting.<br>38 kgP/ha as triple superphosphate and 100 kg K/ha as potassium chloride. Not a LTE.	1997-1998	Pandey et al., 2001
Chikwawa	Malawi	Irrigated maize-rice two crop system	Field, 4 N rates, not a LTE, 10-30 kgP/ha	2007	Fandika et al., 2008

Rice-wheat two cropping systems					
Parwanipur	Nepal, Inceptisol. Rainfall 1550 mm. Max/min temp 36/8.5	Irrigated	Field, 4 N rates, urea, 2 dressings, also N from (NH4)2HPO4	1980-2000	Gami et al., 2001
Bhairahawa	Nepal, Typic Heplaquepts, silty loam. Rainfall 1700 mm. Max/min temp 45/7	Irrigated	Field, 3 N rates. Two splits, 50% as [NH4]2HPO4 and Urea $(NH_2)_2CO$) at the time of planting, and 50% as urea after 25-30 days of planting	1978-2013	Rawal et al., 2017
Ludhiana, Punjab	India, Tolewal loamy Sand. Rainfall 800 mm. Max/min temp 35/18 July/Oct, 23/7 Nov/Apr	Irrigated	Field, 4 N rates, urea and (NH)2HPO4. 3 dressings	1984-1997	Bhandari et al., 2002
Bidhan, West Bengal	India, sandy loam hyperthermic Aeric Haplaquept. Rainfall 1480 mm. Max/min temp 36/12.5	Irrigated	Field, 2 N rates, urea	1986-2004	Majumber et al., 2008

Table SI 1 Extended overview of characteristics of used Long term N trials

Additional references (others are given in main article):

Hijbeek, R., van Ittersum, M.K., ten Berge, H.F., Gort, G., Spiegel, H. and Whitmore, A.P., 2017. Do organic inputs matter–a meta-analysis of additional yield effects for arable crops in Europe. Plant and Soil, 411(1-2), pp.293-303. (here the references can be found to to Albert and Grunert 2013; Bischoff 1995; Klasink and Steffens 1995; Lang et al., 1995; Körschens et al. 2014 and Von Boguslawski, 1995).

Bhandari, A. L. et al. Yield and Soil Nutrient Changes in a Long-Term Rice-Wheat Rotation in India. Soil Science Society of America Journal 66, 162-170 (2002).

Bischoff, R., & Emmerling, R. (2001). Einfluss Kombinierter organischer und mineralischer Düngung auf den Ertrag und Rohproteingehalt von Winterweizen und Wintergerste sowie die Rübenqualität im IOSDV Speyer. Archives of Agronomy and Soil Science, 47(5-6), 445-457.

Fandika, I. R., Zingore, S., Magreta, R., Chiipanthenga, M. & Ndlovu, W. in Annual Report-Soil and Agricultural Engineering Commodity Group, 2007/08 126-132 (Department of Agricultural Research Services Headquarters 2008).

Gami, S. et al. Long-term changes in yield and soil fertility in a twenty-year rice-wheat experiment in Nepal. Biology and Fertility of Soils 34, 73-78, doi:10.1007/s003740100377 (2001).

Majumder, B. et al. Organic Amendments Influence Soil Organic Carbon Pools and Rice-Wheat Productivity. Soil Science Society of America Journal 72, 775-785, doi:10.2136/sssaj2006.0378 (2008).

Jat, M. L., Bijay, S. & Gerard, B. Advances in Agronomy 171-259 (2014).

Pandey, R. K., Maranville, J. W. & Bako, Y. Nitrogen Fertilizer Response and Use Efficiency for Three Cereal Crops in Niger. Communications in Soil Science and Plant Analysis 32, 1465-1482, doi:10.1081/css-100104206 (2007).

Rawal, N., Ghimire, R. & Chalise, D. Crop Yield and Soil Fertility Status of Long-Term Rice-Rice-Wheat Cropping Systems. International Journal of Applied Sciences and Biotechnology 5, 42-50, doi:10.3126/ijasbt.v5i1.17001 (2017).

Rogasik, J., Schroetter, S., Schnug, E., & Kundler, P. (2001). Langzeiteffekte ackerbaulicher Massnahmen auf die Bodenfruchtbarkeit. Archives of Agronomy and Soil Science, 47(1-2), 3-17.

Stanger, T. F., Lauer, J. G. & Chavas, J.-P. The Profitability and Risk of Long-Term Cropping Systems Featuring Different Rotations and Nitrogen Rates. Agronomy Journal 100, 105-113, doi:10.2134/agronj2006.0322 (2008).

Thorp, K. R., Malone, R. W. & Jaynes, D. B. Fertilizer application rates on corn yield and nitrogen dynamics. Transactions of the ASABE 50, 1287-1303 (2007).

Vanotti, M. B., & Bundy, L. G. (1994). An alternative rationale for corn nitrogen fertilizer recommendations. Journal of Production agriculture, 7(2), 243-249.

Wen, Z. et al. Combined Applications of Nitrogen and Phosphorus Fertilizers with Manure Increase Maize Yield and Nutrient Uptake via Stimulating Root Growth in a Long-Term Experiment. Pedosphere 26, 62-73, doi:10.1016/s1002-0160(15)60023-6 (2016).

Site information			Observations			Regression parameters				Regression results			Scaled data	
						Yr = A = B x *Nrate* + C x *Nrate*^{2}							Y-index	N-availa-bility
**Cereal**		Site	*Nrate*	Yield (*Y*)	*Ymax*	*A*	*B*	*C*	R^2	*Nrate* (*Ymax*)	*Nrate* (*Y*=0)	*Yr* max	*Y*/*Ymax*	*Nrate* + *SN*
				grain							*-SN*		*Yr*	Nav
			kgN/ha	t/ha	t/ha	[-]	x 10^3	x 10^5		kgN/ha	kgN/ha	[-]	[-]	kgN/ha
Wheat	1	Limburgerhof	0	2.37	6.49	0.37	7.10	-1.98	1.000	179	-46	1.00	0.37	46
Wheat		Limburgerhof	80	5.27									0.81	126
Wheat		Limburgerhof	120	6.00									0.92	166
Wheat		Limburgerhof	160	6.49									1.00	206
Wheat		Limburgerhof	200	6.44									0.99	246
Wheat	2	Muncheberg	35	3.05	4.83	0.35	9.16	-3.19	0.998	144	-34	1.01	0.63	69
Wheat		Muncheberg	70	4.03									0.83	104
Wheat		Muncheberg	105	4.68									0.97	139
Wheat		Muncheberg	140	4.83									1.00	174
Wheat		Muncheberg	175	4.74									0.98	209
Wheat	3	Oldenburg	0	3.06	7.39	0.42	6.24	-1.69	0.998	185	-58	1.00	0.41	58
Wheat		Oldenburg	50	5.22									0.71	108
Wheat		Oldenburg	100	6.46									0.87	158
Wheat		Oldenburg	150	7.13									0.96	208
Wheat		Oldenburg	200	7.39									1.00	258
Wheat	4	Speyer	0	1.87	6.25	0.31	6.42	-1.50	0.996	214	-44	1.00	0.30	44
Wheat		Speyer	60	4.14									0.66	104
Wheat		Speyer	120	5.43									0.87	164
Wheat		Speyer	180	5.97									0.96	224
Wheat		Speyer	240	6.25									1.00	284
Wheat	5	Sproda	0	2.99	6.79	0.45	7.98	-2.86	0.996	140	-48	1.00	0.44	48

Wheat		Sproda	40	4.92									0.73	88
Wheat		Sproda	80	6.20									0.91	128
Wheat		Sproda	120	6.57									0.97	168
Wheat		Sproda	160	6.79									1.00	208
Wheat	6	Rauischholzhausen	0	3.00	6.20	0.49	5.86	-1.68	0.986	174	-69	1.00	0.48	69
Wheat		Rauischholzhausen	60	4.80									0.77	129
Wheat		Rauischholzhausen	100	5.80									0.94	169
Wheat		Rauischholzhausen	150	5.90									0.95	219
Wheat		Rauischholzhausen	200	6.20									1.00	269
Wheat	7	Vienna	0	3.06	5.24	0.60	5.36	-1.80	0.969	149	-87	1.00	0.58	87
Wheat		Vienna	50	4.55									0.87	137
Wheat		Vienna	100	4.84									0.92	187
Wheat		Vienna	150	5.24									1.00	237
Wheat		Vienna	200	5.02									0.96	287
Wheat	8	Grabow	0	2.75	6.77	0.40	9.71	-3.92	0.999	124	-36	1.00	0.41	36
Wheat		Grabow	40	4.88									0.72	76
Wheat		Grabow	80	6.34									0.94	116
Wheat		Grabow	120	6.77									1.00	156
Wheat	9	Bologna	0	3.33	5.50	0.62	4.05	-0.97	0.964	209	**-119**	1.04	0.61	#N/A
Wheat		Bologna	100	5.33									0.97	#N/A
Wheat		Bologna	200	5.50									1.00	#N/A
Wheat		Bologna	300	5.37									0.98	#N/A
Wheat	10	Novi Sad	0	3.51	5.59	0.65	6.34	-2.63	0.935	120	-78	1.03	0.63	78
Wheat		Novi Sad	50	5.40									0.97	128
Wheat		Novi Sad	100	5.50									0.98	178
Wheat		Novi Sad	150	5.59									1.00	228
Wheat		Novi Sad	200	4.91									0.88	278
Wheat	11	Madrid	0	2.47	3.55	0.68	7.25	-4.67	0.931	78	-66	0.96	0.69	66
Wheat		Madrid	30	2.95									0.83	96

Wheat		Madrid	60	3.30								0.93	126
Wheat		Madrid	90	3.55								1.00	156
Wheat		Madrid	120	3.04								0.86	186
Wheat	12	India, Punjab	0	2.57	5.65	0.43	7.01	-2.26	0.969	155	-53	0.98	0.45	53
Wheat		India, Punjab	43	3.61									0.64	96
Wheat		India, Punjab	80	4.82									0.85	133
Wheat		India, Punjab	123	5.65									1.00	175
Wheat		India, Punjab	160	5.36									0.95	213
Wheat	13	India, Haryana	0	2.15	4.03	0.48	4.71	-1.06	1.000	222	-85	1.00	0.48	85
Wheat		India, Haryana	60	3.25									0.72	145
Wheat		India, Haryana	123	4.03									0.90	208
Wheat	14	India, Punjab2	43	3.30	5.00	0.40	6.71	-1.80	1.000	187	-53	1.03	0.66	96
Wheat		India, Punjab2	95	4.40									0.88	148
Wheat		India, Punjab2	145	5.00									1.00	198
Wheat	15	Pakistan, Punjab	0	2.20	3.90	0.57	5.36	-1.69	0.994	159	-84	0.99	0.56	84
Wheat		Pakistan, Punjab	80	3.50									0.90	164
Wheat		Pakistan, Punjab	95	3.60									0.92	179
Wheat		Pakistan, Punjab	123	3.70									0.95	206
Wheat		Pakistan, Punjab	145	3.90									1.00	229
Wheat	16	India, Uttar Pradesh	0	2.00	3.78	0.51	5.95	-1.91	0.969	156	-71	0.98	0.53	71
Wheat		India, Uttar Pradesh	60	2.87									0.76	131
Wheat		India, Uttar Pradesh	123	3.78									1.00	193
Wheat		India, Uttar Pradesh	180	3.59									0.95	251
Wheat	17	Pakistan, Punjab2	80	3.60	5.16	-0.17	14.46	-4.50	0.982	161	12	0.99	0.70	68
Wheat		Pakistan, Punjab2	123	4.77									0.92	110
Wheat		Pakistan, Punjab2	145	5.16									1.00	133
Wheat		Pakistan, Punjab2	180	4.92									0.95	168
Wheat		Pakistan, Punjab2	205	4.73									0.92	193
Wheat	18	Pakistan, Punjab3	0	3.73	4.78	0.79	1.32	-0.15	0.958	429	**-404**	1.07	0.78	#N/A

Wheat		Pakistan, Punjab3	73	4.26									0.89	#N/A
Wheat		Pakistan, Punjab3	145	4.43									0.93	#N/A
Wheat		Pakistan, Punjab3	205	4.78									1.00	#N/A
Wheat	19	Bangladesh	43	2.66	3.73	0.50	5.59	-1.48	1.000	189	-75	1.03	0.71	118
Wheat		Bangladesh	95	3.35									0.90	170
Wheat		Bangladesh	145	3.73									1.00	220
Wheat	20	China, Lhaijang	0	1.36	2.74	0.50	9.11	-3.96	1.000	115	-45	1.02	0.50	45
Wheat		China, Lhaijang	69	2.56									0.94	99
Wheat		China, Lhaijang	138	2.74									1.00	168
Barley	21	Oldenburg	0	3.06	6.78	0.46	7.14	-2.39	0.995	149	-55	1.00	0.45	55
Barley		Oldenburg	40	4.97									0.73	95
Barley		Oldenburg	80	5.91									0.87	135
Barley		Oldenburg	120	6.52									0.96	175
Barley		Oldenburg	160	6.78									1.00	215
Barley	22	Speyer	0	1.46	5.53	0.27	8.16	-2.25	0.998	181	-30	1.01	0.26	30
Barley		Speyer	50	3.47									0.63	80
Barley		Speyer	100	4.64									0.84	130
Barley		Speyer	150	5.53									1.00	180
Barley		Speyer	200	5.50									1.00	230
Maize	23	USA, IA, Story City	0	3.00	8.60	0.37	6.19	-1.52	0.965	204	-53	1.00	0.33	53
Maize		USA, IA, Story City	50	5.90									0.66	103
Maize		USA, IA, Story City	100	8.10									0.90	153
Maize		USA, IA, Story City	150	8.50									0.95	203
Maize		USA, IA, Story City	200	8.60									0.96	253
Maize		USA, IA, Story City	250	8.30									0.92	303
Maize		USA, IA, Story City	300	8.10									0.90	353
Maize	24	USA, KS, Tribune (40 kg P/ha, 2006)	0	4.30	11.30	0.37	6.98	-1.92	0.984	182	-46	1.00	0.37	46
Maize		USA, KS, Tribune (40 kg P/ha, 2006)	45	8.10									0.70	91
Maize		USA, KS, Tribune (40 kg P/ha, 2006)	90	10.10									0.87	136

Maize		USA, KS, Tribune (40 kg P/ha, 2006)	135	11.00								0.95	181
Maize		USA, KS, Tribune (40 kg P/ha, 2006)	179	11.30								0.97	226
Maize		USA, KS, Tribune (40 kg P/ha, 2006)	224	11.30								0.97	271
Maize		USA, KS, Tribune (40 kg P/ha, 2006)	0	4.50	12.80	0.37	6.98	-1.92	0.984	182	-46	1.00	0.35	46
Maize		USA, KS, Tribune (40 kg P/ha, 2006)	45	7.70								0.59	91
Maize		USA, KS, Tribune (40 kg P/ha, 2006)	90	10.70								0.82	136
Maize		USA, KS, Tribune (40 kg P/ha, 2006)	135	12.70								0.98	181
Maize		USA, KS, Tribune (40 kg P/ha, 2006)	179	12.50								0.96	226
Maize		USA, KS, Tribune (40 kg P/ha, 2006)	224	12.80								0.99	271
Maize	25	USA, WI, Lancaster	0	3.30	9.10	0.34	6.34	-1.53	0.988	207	-49	1.00	0.36	49
Maize		USA, WI, Lancaster	56	5.60								0.61	105
Maize		USA, WI, Lancaster	112	8.20								0.89	161
Maize		USA, WI, Lancaster	224	9.10								0.99	273
Maize	26	China, Changping	0	3.80	7.80	0.57	4.26	-1.06	0.900	201	-106	0.99	0.48	106
Maize		China, Changping	135	7.30								0.92	241
Maize		China, Changping	270	7.80								0.98	376
Maize		China, Changping	0	5.30	7.90								0.66	106
Maize		China, Changping	135	7.90								0.98	241
Maize		China, Changping	270	7.30								0.91	376
Maize	27	China, Lhaijang	0	3.55	6.31	0.56	8.56	-3.90	1.000	110	-53	1.03	0.56	53
Maize		China, Lhaijang	69	6.11								0.97	122
Maize		China, Lhaijang	138	6.31								1.00	191

Table SI 2 Observations and results of regressions and scaling for used Long term N trials

Note 2 Broadbalk wheat experiment, Rothamsted Research, UK

The Broadbalk wheat experiment is one of the oldest continuous agronomic experiments in the world. Started by Lawes and Gilbert in the autumn of 1843, wheat has been sown and harvested on all or part of the field every year since then. The original aim of the experiment was to test the effects of various combinations of inorganic fertilizers (supplying N, P, K, Na and Mg) and different organic manures on the yield of winter wheat; a control strip has received no fertilizer or organic manures since 1843. For the first few years these treatments varied a little, but in 1852 a scheme was established that continues, with some modifications, today[53] (http://www.era.rothamsted.ac.uk/Broadbalk).

**Strip**	5	6	7	8	9	15	16
**kgN/ha 1985-2018**	0	48	96	144	192	240	288
**kgN/ha 1852-1984**	0	48	96	144	192 (1968-1984)<br>48 (1852-1967)	144 (1968-1984)<br>96 (1852-1967)	96

Table SI 3 **Broadbalk annual fertilizer N treatments** used in this study, all with non-limiting PKMg.

Winter wheat varieties: Bread making varieties, selected for their yield potential. 1985-1990 Brimstone; 1991-1995 Apollo; 1996-2012 Hereward; 2013-2018 Crusoe. Data for 2015 is omitted as spring wheat was sown, due to a very wet autumn/winter.

Continuous wheat v 1st wheat in rotation: Originally the whole experiment was in continuous wheat, with occasional fallowing to control weeds. In 1926 the experiment was divided into five Sections, crossing all the treatment strips, and bare fallowed sequentially to control weeds. In 1968 the experiment was divided into 10 Sections, to allow the comparison of wheat grown continuously with wheat grown in rotation with other arable crops. The rotations for this study period were: 1985-1995: wheat>wheat>wheat>fallow>potatoes; 1996-2018: wheat>wheat>wheat>oats> forage maize. No fertilizer N was applied to the fallow or oats treatments; the potatoes and forage maize received fertilizer N at the same rate as the wheat. Yields and crop N uptake from the first wheat in the rotation, from Sections 2-5 and 7 were compared with wheat grown continuously in Section 1 (last fallowed in 1966). Yields are at 85% dry matter.

General agronomy: All plots in this study have received adequate P (35kg/ha as triple superphosphate), K (90 kgK/ha as potassium sulphate) and Mg (12 kgK/ha as Kieserite). N is applied in the spring as ammonium nitrate; lime is applied as necessary. Applications of herbicides, fungicides and insecticides follow standard farm practice. The experiment is ploughed every autumn and straw is removed at harvest by baling. There is no irrigation.

Site details: Rothamsted Research, south east England, 51.807 N, -0.360 E. Clay loam to silty clay loam over clay-with flints, Chromic Luvisol (FAO Classification), 28% clay.

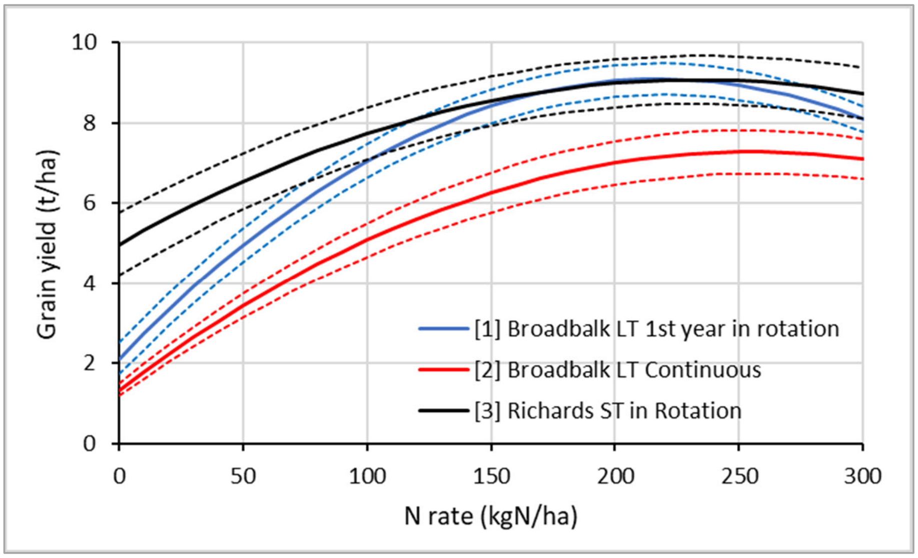

***Fig. SI 1 Long- and short-term N response for winter wheat in the UK**: 1. LT N response for winter wheat in first year after preceding crop in rotation, 2. LT N response for winter wheat in continuous cultivation as observed in Broadbalk, and 3. the classical 1st year N response for winter wheat in rotation (dashed lines, 95% Confidence Intervals).*

LTEs for continuous wheat and wheat in rotation In Broadbalk have both different *Yo* and *Ymax* as compared to wheat in rotation (Fig SI1). One reason for the higher *Yo* (0.4 t/ha) - and more in general yields at low N rates - for wheat in rotation compared to continuous wheat is the N availability from residues from preceding crops (in Broadbalk potato, oat or forage maize). Among the two LTEs, wheat in rotation shows a *Ymax* that is 1.3 t/ha higher than for continuously cropped wheat, as continuous cropping incurs a higher burden of pests and disease.

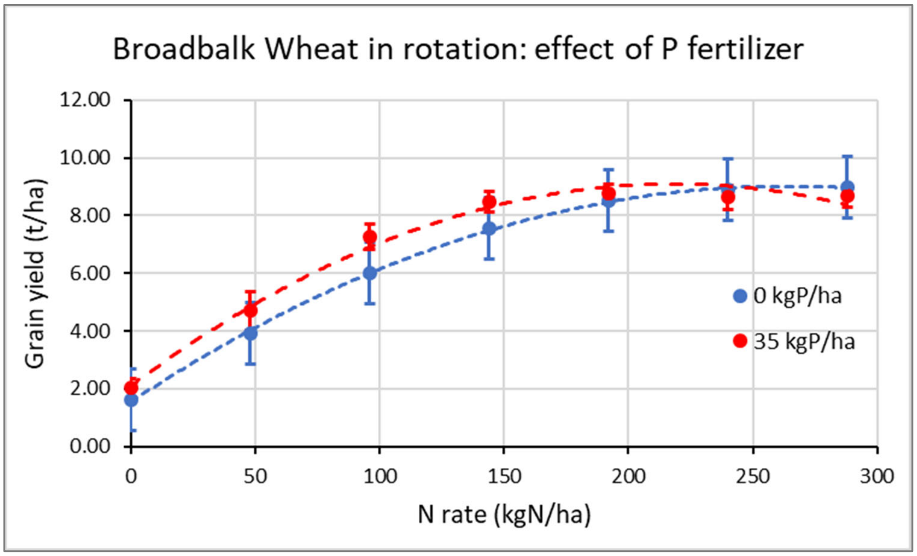

***Fig SI 2 N response curves for winter wheat in rotation in the UK for P inputs** of 0 and 35 kgP/ha and 95% error bars (Rothamsted, 2019).*

Note 3 Procedures for data scaling and curve fitting

Transformation of N fertilizer rate to total N availability

The sum of N inputs from these other N sources (*SN*) is approximated by the X intercept of the 2nd order polynomial fit (see example below where this N input is 54 kgN/ha).

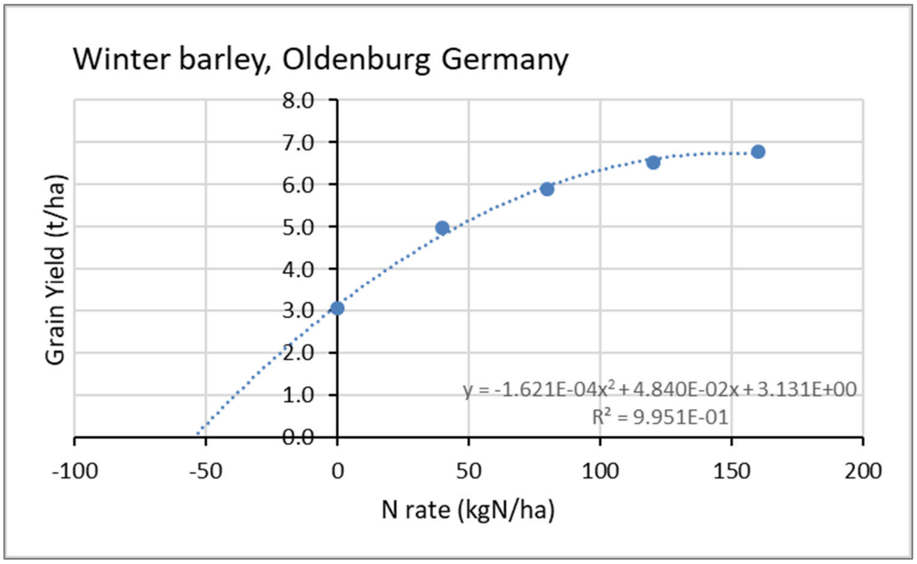

***Fig SI 3 Estimation of N input from other sources than added fertilizer (SN)** by extrapolation of the fitted 2nd order polynomial of the yield response to N rate for Y=0. SN includes net soil N mineralization**,** atmospheric N deposition, BNF.*

Alternative functions to describe yield response to nitrogen input

For this research we chose to fit response of relative yield to N availability (*Nav*) by a 2nd order polynomial with zero intercept, as then optima and cross points simply follow from standard calculus for solution of quadratic functions.

Many different response models have been proposed in literature, some with clear agronomic / biophysical meaning attached to their parameters. Those models are of exponential, power, quadratic, spherical or Michaelis-Menten nature; or they construct yield response in various steps from nutrient availability, apparent recovery and attainable ranges of plant nutrient concentrations, as in the case of the QUEFTS model (Janssen et al., 1990). All such models express diminishing yield returns with increasing N availability. In all cases, however, they express the response of absolute (rather than relative) yield Y, to N rate (or N availability). If such models are scaled by dividing both left- and right-hand sides of the equation by attainable yield *Ymax*, then relative yield (Y/*Ymax*) becomes an expression of not only N rate (or N availability), but also of *Ymax*. (Scaling either introduces *Ymax* on the RHS, or does not entirely remove it, depending on the model.) Relative yield then somehow depends on N availability relative to *Ymax*. That is essentially different from our simple polynomial expression, which relates relative yield to **j**ust N availability. This implies that agronomic N use efficiency in our model is directly proportional to *Ymax*; unlike in other models.

Below we show result of fitting observed response of relative yield to N availability in 25 long-term field trials by (Table SI 1) by other mathematical functions to show that a 2nd order polynomials (with zero intercept) fit equally well. All three models give *Y=0* at *Nav=0* and a *Ymax* or Y plateau but are normally used to fit unscaled N response data.

1. A Michaelis-Menten type model as used in Lassaletta et al. (2014) to model N removal as function of N rate:

   $Y/Ymax = [Ymax \times Nc \times Nav]/[Ymax \times Nc + Nav]^b$ (Eq. SI 1)

   With Nc as relative N content in grain needed to convert Y to kgN/ha
2. A Mitscherlich type model as used in Dobermann et al., (2011)[43] to model short-term N response:

   $Y/Ymax = a - b \times e^{-c \times Nav}$ (Eq. SI 2)
3. The George model as used in Hijbeek et al. (2017) to model short-term N response:
   $Y/Ymax = a \times 0.99^{Nav} + b \times Nav$ (Eq. SI 3).

R^2 for the correlation between predicted and observed *Y/Ymax* (N=121) was 0.71 for the Michaelis-Menten model, 0.85 for the Mitscherlich model, and 0.87 for the George model, as compared to 0.84 for our quadratic function. In short, fits of the various models are quite similar.

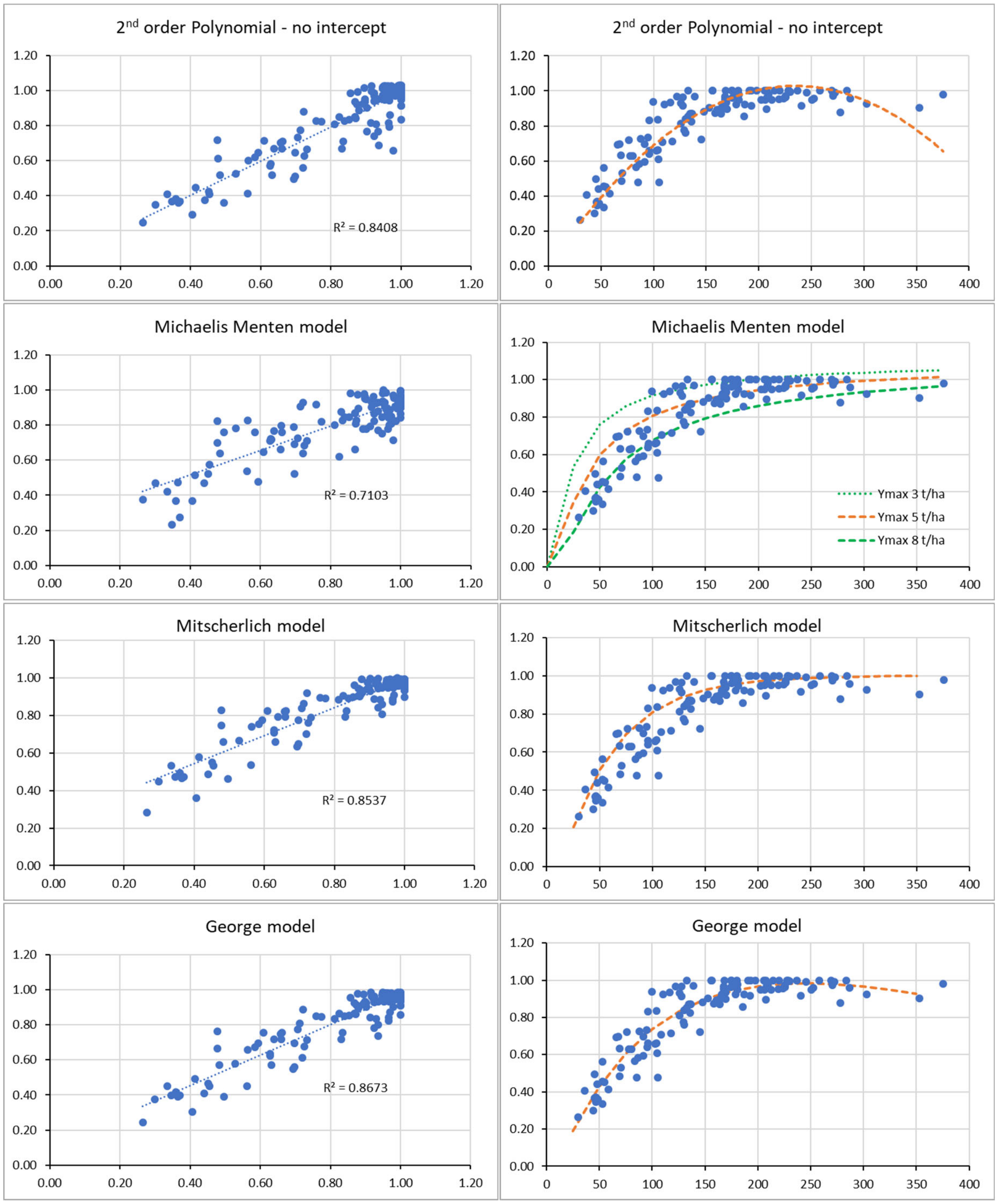

Fig SI 4 Alternative functions for a 2nd order polynomial *to fit response of relative yield (Y/Ymax) to N availability (kgN/ha); Left hand side scatterplot of predicted against observed Yr and* R^2*, right hand side the fitted response curve*

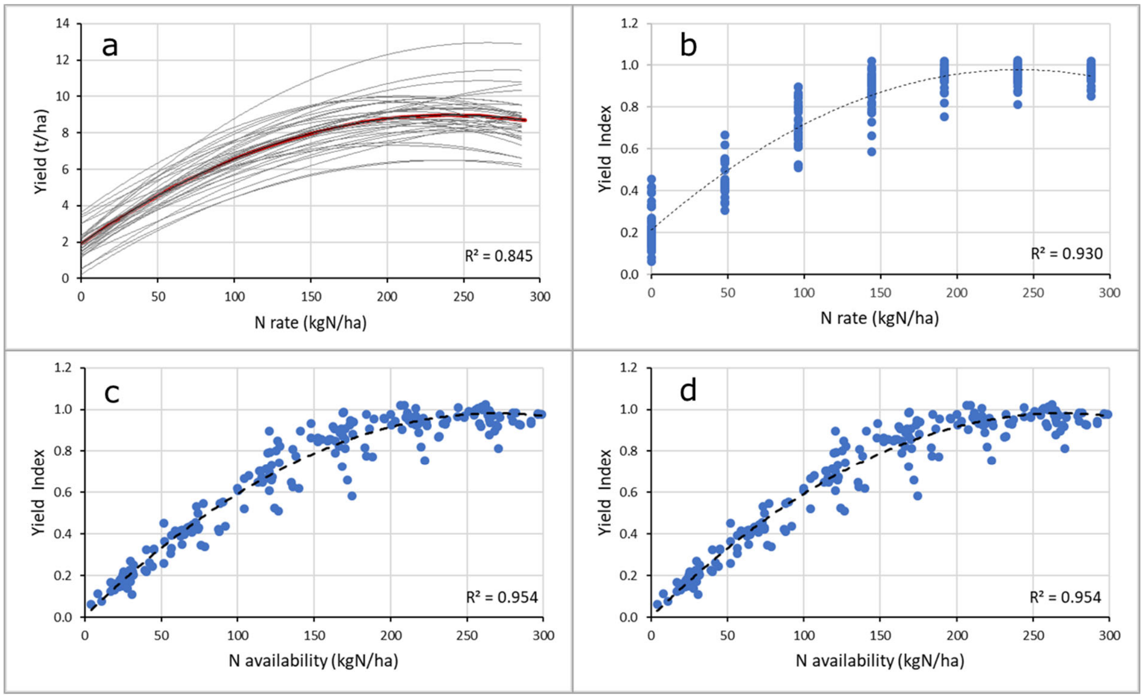

***Fig. SI 5 Effect of scaling on annual N response curves for winter wheat in rotation** from 1985 to 2018 for winter wheat in rotation at Broadbalk, Rothamsted. **a**: 2nd order polynomial fits of annual N response curves from unscaled observations and mean curve; **b**: for yields indexed to maximum annual yield; **c**: as Fig. SI 4b with N rates per year of observation transformed to available N by adding estimates of non-fertilizer sources (SN); **d**: as Fig. SI 4b but with a forced zero intercept for 2nd order polynomial N response to derive Eq 1.*

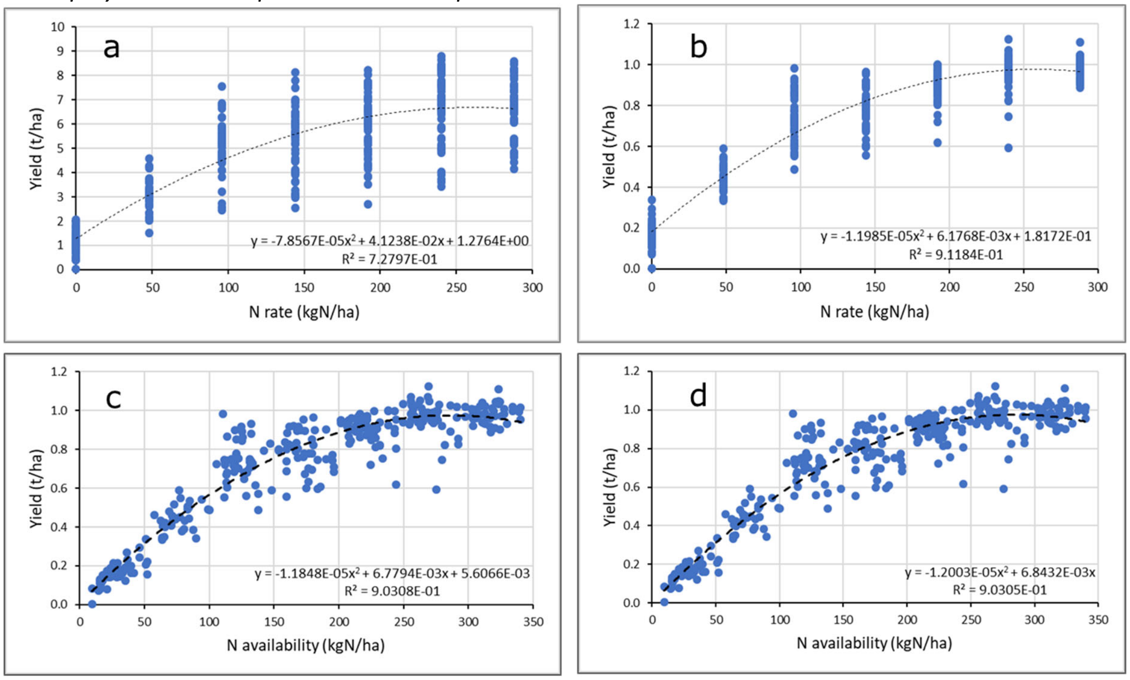

***Fig. SI 6 Effect of scaling on annual N response curves for continuous winter wheat** from 1985 to 2018 at Broadbalk, Rothamsted. **a**: 2nd order polynomial fits of annual N response curves from unscaled observations and mean curve; **b**: for yields indexed to maximum annual yield; **c**: as Fig. SI 4b with N rates per year of observation transformed to available N by adding estimates of non-fertilizer sources (SN); **d**: as Fig. SI 4b but with a forced zero intercept for 2nd order polynomial N response to derive Eq 1.*

The R^2 of the fits for N response for the individual years for winter wheat in rotation ranged between 0.943 and 0.999, for continuous winter wheat between 0.841 and 0.991.

Resulting generic curvatures are:

Wheat in rotation: $Yr = -1.354E\text{-}5 \times Nav^2 + 7.291E\text{-}3 \times Nav$ (Eq. SI 4, R^2 0.954; N=245)

	Value	*Stand. Error*	*t Stat*	*P-value*	*Lower 95%*	*Upper 95%*
Coefficient 1	7.291E-03	8.541E-05	8.536E+01	3.429E-183	7.123E-03	7.460E-03
Coefficient 2	-1.354E-05	3.214E-07	-4.214E+01	1.050E-113	-1.418E-05	-1.291E-05

Wheat continuous: $Yr = -1.200E\text{-}5 \times Nav^2 + 6.843E\text{-}3 \times Nav$ (R^2 0.903; N=322).

	Value	*Stand. Error*	*t Stat*	*P-value*	*Lower 95%*	*Upper 95%*
Coefficient 1	6.843E-03	1.027E-04	6.662E+01	1.271E-189	6.641E-03	7.045E-03
Coefficient 2	-1.200E-05	3.916E-07	-3.065E+01	3.237E-97	-1.277E-05	-1.123E-05

The scaled curves are very similar with a mean difference in *Yr* of 4% at a given *Nav*. This is to be expected for the same crop, grown on the same soil under the same climate.

Additional references

Janssen, B. H., Guiking, F. C. T., Van der Eijk, D., Smaling, E. M. A., Wolf, J., & Van Reuler, H. (1990). A system for quantitative evaluation of the fertility of tropical soils (QUEFTS). Geoderma, 46(4), 299-318.

Richards, I. R. Energy balances in the growth of oilseed rape for biodiesel and of wheat for bioethanol. 38 (Levington Agriculture Ltd. Levington Park lPSWlCH Suffolk IP10 2000).

Note 4 Long term N response of maize in the USA

For maize (corn) we found five LTEs (Table 1). The LTEs for irrigated maize in Kansas, USA stand out with 6 N rates and having run since 1961 (Schlegel et al., 2017)[27]. In view of its duration the soil N status likely is in steady state with the N rates and low yields are expected at zero N input. To verify this, annual results between 2001 and 2010 were analyzed as demonstrated for Rothamsted wheat in rotation

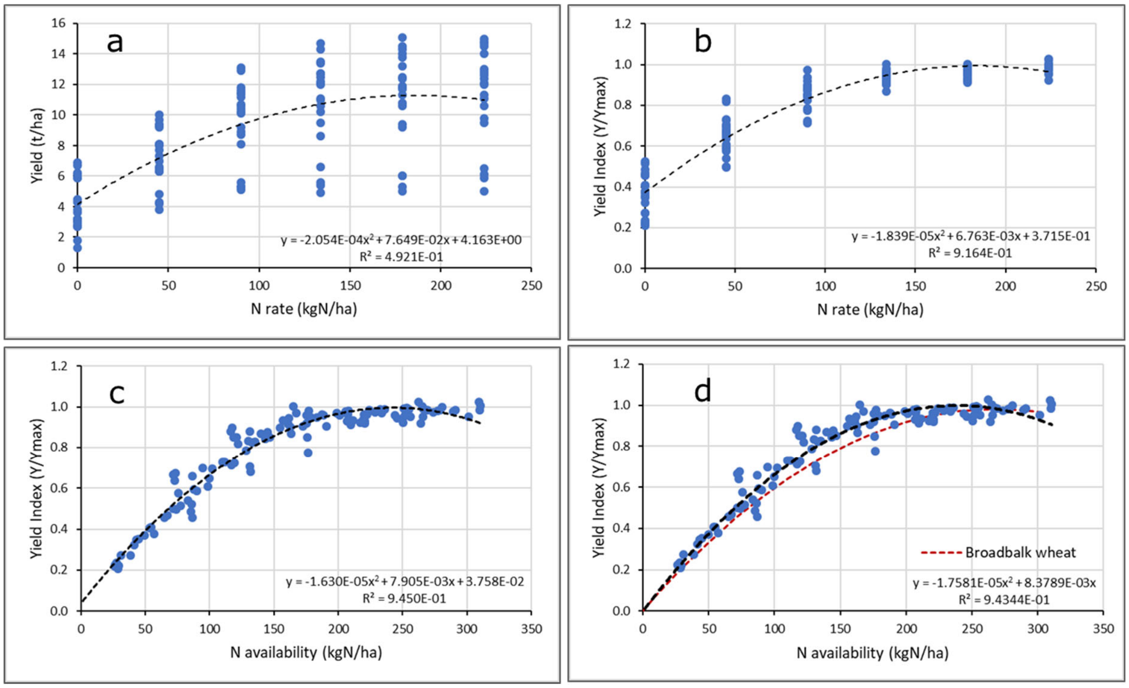

***Fig. SI 7 Results of for irrigated continuous maize in Tribune Kansas, USA** (data 2001-2010; scaling and fitting 2nd order polynomials on annual N response curves; respectively, **a:** original observations; **b**: indexed yield; **c**: transformed N rates and **d**: as **c** with forced zero intercept and comparison to scaled N response curve for wheat in rotation in Rothamsted.*

The resulting generic N response relationship for continuous maize is:

$$Yr = -1.758E\text{-}5 \times Nav^2 + 8.379\ E\text{-}3 \times Nav \qquad \text{(Eq. SI 5, } R^2\ 0.934\text{; N=120).}$$

	Value	*Stand. Error*	*t Stat*	*P-value*	*Lower 95%*	*Upper 95%*
Coefficient 1	8.379E-03	1.105E-04	7.580E+01	6.168E-102	8.160E-03	8.598E-03
Coefficient 2	-1.758E-05	4.747E-07	-3.704E+01	8.051E-67	-1.852E-05	-1.664E-05

However, maize yields at zero N input each year are on average 38% of the maximum yield in that year, as compared to 22% in Rothamsted. N deposition at Tribune is around 8 kg/N/ha (Zhang et al., 2012) and lower than in Rothamsted, but irrigation provides an additional N input around 10 kgN/ha. However, the total background input would not allow a long term sustainable maize yield of around 4 t/ha, which suggest other N sources at Tribune or ongoing soil N depletion. The fully scaled N response curve with no intercept for maize at Tribune shows a stronger N response than for wheat in rotation at Rothamsted, which was also found for the LTE's for wheat in Europe and Asia. Results may suggest that scaling of LTE's yields generic N response curves which still slightly overestimate N response in the intermediate range of N rates.

Stanger et al (2006) investigated the effect of various rotations on maize yields in LTEs at Lancaster Wisconsin. Precession of maize by N fixing crops like soybean and alfalfa increased *Ymax* from 9.2 t/ha to 10.3 and 10.8 t/ha, respectively, but the yield increase could be fully explained by the increase of *Nav* as estimated from extrapolation of the fitted 2nd order polynomial to Y=0 (see Supplementary Note 3). *SN* for Corn-Soy was 140 kgN/ha for Corn-Alfalfa 216 kgN/ha. These results indicate that the generic N response approach also is applicable for rotations of cereals and legumes.

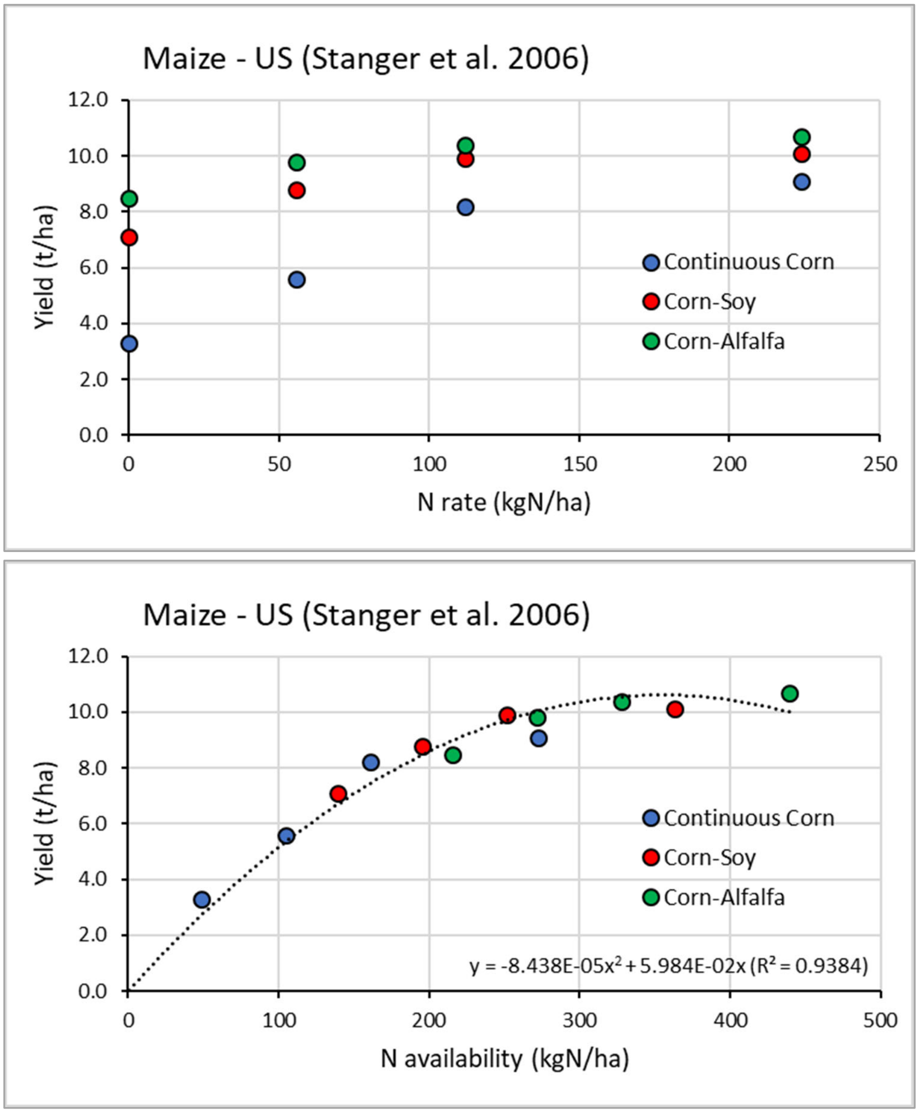

***Fig. SI 8 Effect of rotations with legumes on response of maize yields** to N rate and to total N availability as observed in LTEs in Wisconsin, USA*

Additional reference

Stanger, T. F., Lauer, J. G. & Chavas, J.-P. The Profitability and Risk of Long-Term Cropping Systems Featuring Different Rotations and Nitrogen Rates. Agronomy Journal 100, 105-113, doi:10.2134/agronj2006.0322 (2008).

Schlegel, A. J. & Havlin, J. L. Corn Yield and Grain Nutrient Uptake from 50 Years of Nitrogen and Phosphorus Fertilization. Agronomy Journal 109, 335-342, doi:10.2134/agronj2016.05.0294 (2017).

Thorp, K. R., Malone, R. W. & Jaynes, D. B. Fertilizer application rates on corn yield and nitrogen dynamics. Transactions of the ASABE 50, 1287-1303 (2007).

Zhang, L., Jacob, D. J., Knipping, E. M., Kumar, N., Munger, J. W., Carouge, C. C., ... & Chen, D. (2012). Nitrogen deposition to the United States: distribution, sources, and processes. Atmospheric Chemistry and Physics.

Note 5 Statistics and cross validation of scaled yield response curve for global cereals

The 2nd order polynomial fit of pooled scaled N response data for wheat, maize and barley was:

$$Yr = -1.870E\text{-}5 \times Nav^2 + 8.768E\text{-}3 \times Nav \qquad (R^2\ 0.818;\ N=119)$$

	Value	*Stand. Error*	*t Stat*	*P-value*	*Lower 95%*	*Upper 95%*
Coefficient 1	8.77E-03	1.54E-04	5.71E+01	2.90E-87	8.46E-03	9.07E-03
Coefficient 2	-1.87E-05	6.76E-07	-2.76E+01	4.04E-53	-2.00E-05	-1.74E-05

The validity of the LT generic N response curve was verified by back calculation of the original unscaled cereal grain yields for every LTE, using 25 alternative 2nd order polynomial fits of the dataset of indexed yield as a function of *Nav*, each time leaving out the observations for the validation site.

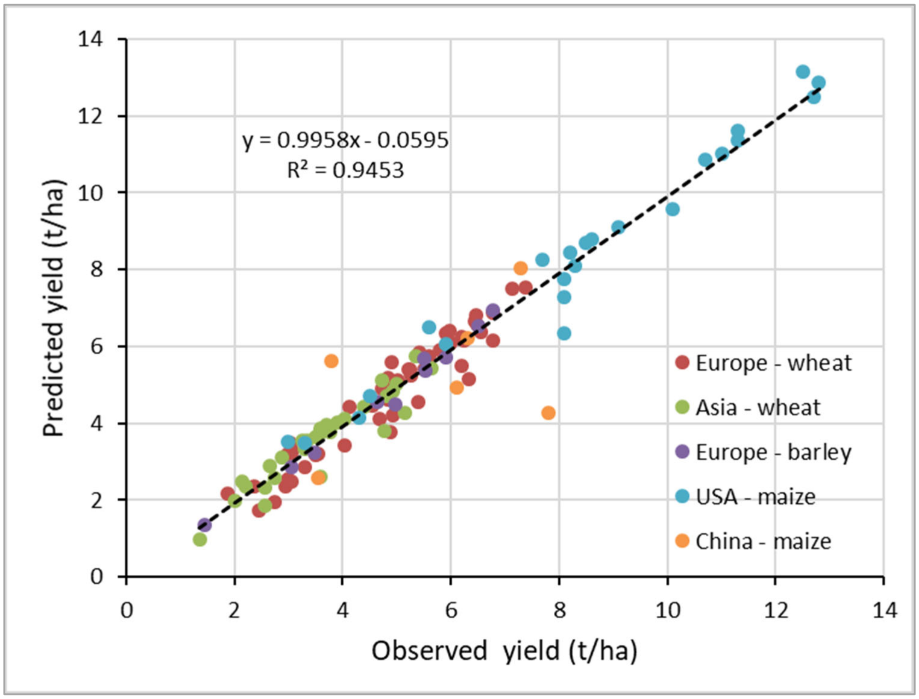

***Fig. SI 9 Cross validation of scaled yield response curve for global cereals**, where unscaled yields per site and applied N rate are back calculated using 25 alternative 2nd order polynomials fits of the dataset of indexed yield as a function of Nav, each time leaving out the observations for the validation site.*

Note 6 Validation of generic N response at country scale for Europe and Africa

To validate the scaled generic N response relation for Europe we plotted scaled grains yield for rainfed wheat, barley and maize by Schils et al., (2019)[31] against N availability inferred from modelled N uptake (Fig. SI 11). For this yield were indexed to 90% of *Yw* as published by the Global Yield Gap Atlas (http://www.yieldgap.org/web/guest/home). When N availability is set at 78% of N uptake, data points per country can be well described by 2nd order polynomial with no intercept (R^2 0.796) and fall in between the scale N response curve for wheat in rotation at Broadbalk and for the 25 global LTEs.

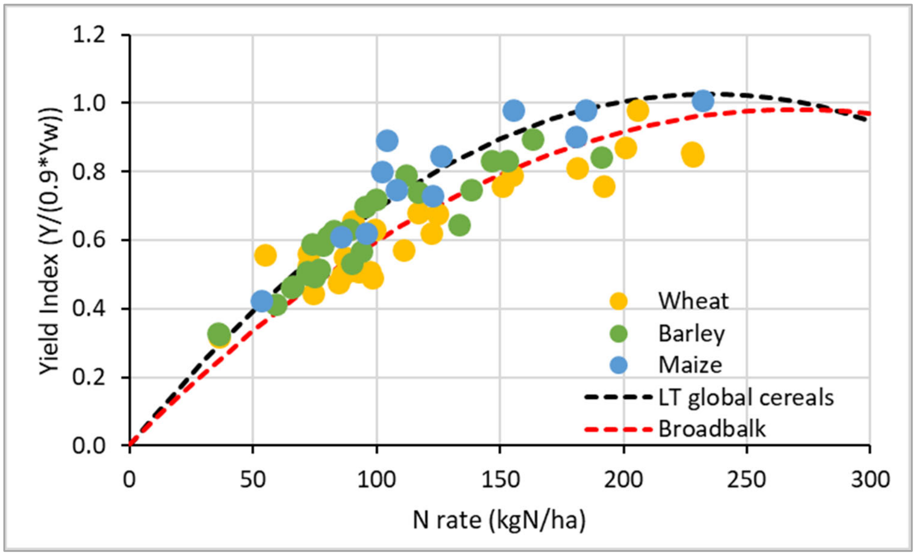

Fig SI 10 Scaled N response in Europe for wheat, barley and maize at country level, *as compared to scaled N response for global cereals and for winter wheat in Broadbalk*

The explanation of a fairly good match between our scaled N response curve and a response curve based on the variation of current national mean values of cereal yield and N fertilizer rates per hectare in Europe is that these national values represent near steady state between N inputs and soil N stocks. Such a steady state is quite plausible as in Europe N rates and *Ymax* have been fairly stable in the past decades. We found a similar good match when using data from Fertilizer Europe (2019) based on farm surveys.

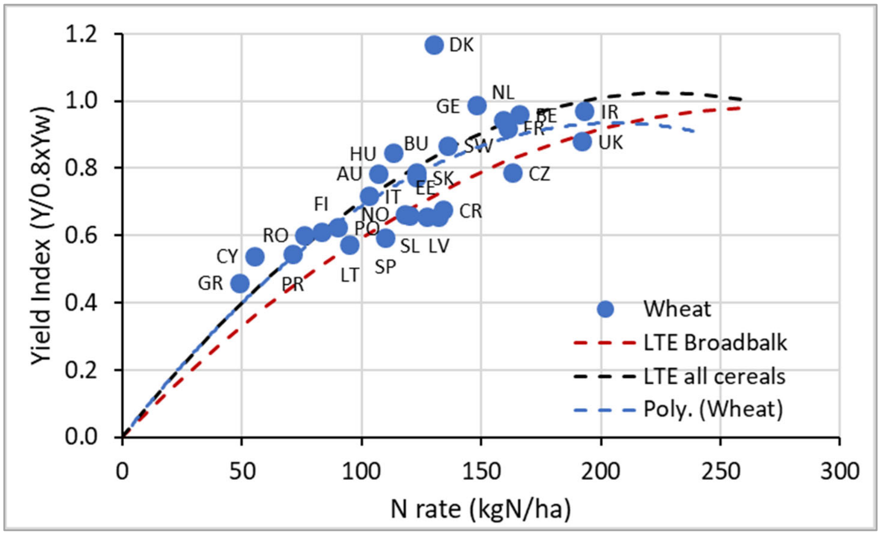

Fig SI 11 Scaled N response in Europe for wheat at country level, *as compared to scaled N response for global cereals and for winter wheat in Broadbalk*

When using N fertilizer rates based on national fertilizer statistics, and fertilizer attribution rules for individual crops as used in the Miterra model (Velthof et al., 2009) a poor match was found with our scaled N relationship. Quantification of representative national, crop specific N fertilizer rates is a notoriously difficult problem (Conant et al., 2013).

Ten Berge et al. (2019)[8] estimated LT (A_{LT}) N-requirement for nine countries in sub-Saharan Africa (Burkina Faso, Ethiopia, Ghana, Kenya, Mali, Nigeria, Tanzania, Uganda and Zambia) for maize self-sufficiency in 2050 (target yield, Y_T), for two scenarios. The Scenario-I matches maize production and demand at country level, and maize does not compensate for shortfall in yield potential of non-maize cereals. Scenario-II matches total cereal production and demand at regional level (West or East Africa) and extra maize production compensates for shortfall in yield potential of non-maize cereals. Model variables were *Yw* (water-limited maize yield, potential), Y_T (target maize yield; 15.5% moisture), relative maize yield (Y_T/Yw), yield increment ratio (Y_T/Ya), long-term minimum input requirements of nitrogen (N), phosphorus (P) and potassium (K). All variables are area-weighted means per country or region.

$$A_{LT} = Y_T / AE_{LT}$$

Where agronomic efficiency (AE, kg grain per kg N applied) is a function of recovery of fertilizer N (typically around 0.6 for ST) and internal N efficiency (for details see Ten Berge et al. (2019)[10]. For Fig. SI 7 we indexed yield with *0.8*Yw* to force a maximal index yield of 1. LT N requirement was converted *Nav* by adding national average *DEP* and *BNF* values for arable agriculture for 2010[26].

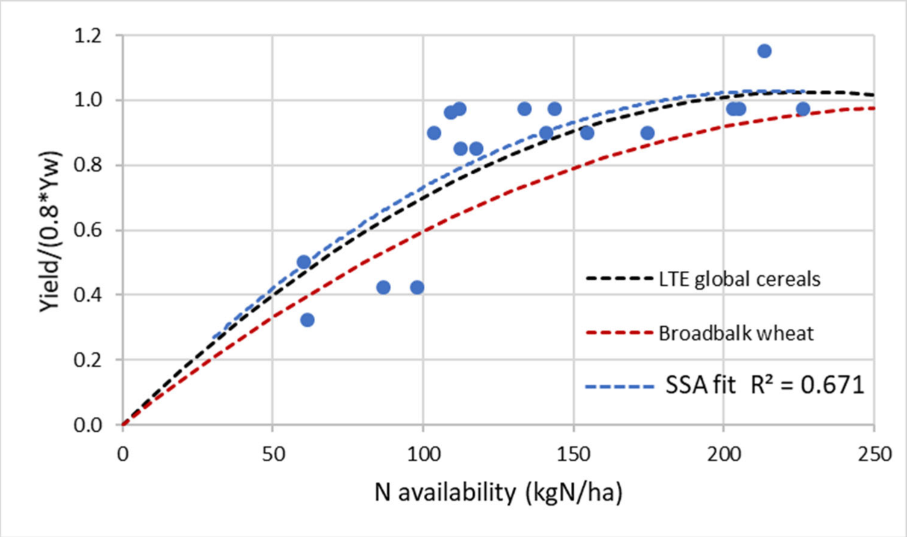

***Fig. SI 12 N response of maize in Sub-Saharan Africa** inferred from modelled LT N requirement for national and regional food sufficiency[8], and 2nd order polynomial fit with zero intercept as compared to generic LT N response for global cereals (Eq 5) and winter wheat in Broadbalk, UK (Eq 1).*

Applicability of the generic curve for Africa was further verified. We looked for suitable LT trials for maize from, generally, short-term nutrient response trials across different locations in sub-Saharan Africa as aggregated by OFRA (OFRA, 2017), and selected trials in Niger (Pandey et al., 2007) and in Malawi (Fandika et al., 2008). For details of trial see Table SI 1. These trials did not meet our criteria for a duration of more than 15 years. However, because these sites had a 'low' fertilizer history, the low-N rates in these trials were most likely in near-equilibrium and the ST and LT N response curves are similar (Fig. 2 main article).

Trials in Niger were carried out at three sites, with maize, pearl millet and sorghum and response data were available for 1997 and 1998. Maximum observed yields were 4.4, 2.4 and 3.4 t/ha for pearl millet, sorghum and maize, respectively. The N availability from net soil N mineralization, N deposition, *BNF* and other sources, as inferred from regression analysis range between 30 and 50 kgN/ha. The scaled response data are well described (R^2 0.875) by the 2nd order polynomial with no intercept, which falls in between the scaled curves for global cereals and for winter wheat in Rothamsted (Fig SI 8).

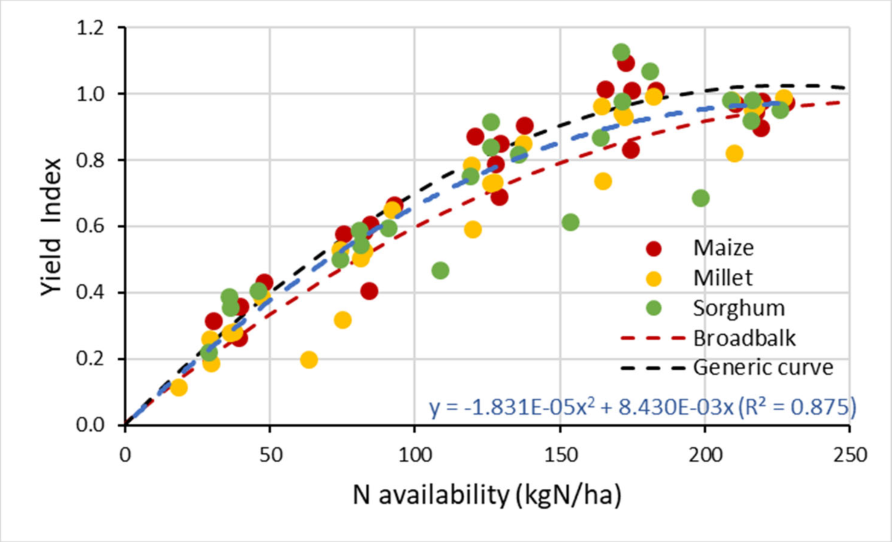

Fig. SI 13 Scaled N response in Niger *for maize, millet and sorghum as compared to scaled N response for global cereals and for winter wheat in Rothamsted*

The maximum yield for Malawi was 2 t/ha, and the N availability from other sources was estimated at 18 kgN/ha. The scaled N response for Maize in Niger corresponded with the generic N response curve, while in Malawi N response was stronger reaching the *Ymax* at a total N input of 100 kg/ha (80kgN/ha of mineral fertilizer (Fig. SI 9). Repeating the regression and cross validation as in Fig. SI 6 including the African sites indicates that the generic N response curve also is applicable for low yield regions as in Africa (Fig. SI 10). However, we stress that the duration of the two African trials does not satisfy our criterion for being in steady state. The relatively low yields at zero fertilizer input for African sites, which for the LT trials at Rothamsted and Kansas are an indication of steady state, here may be caused by a low N fertilizer history, while the attainment of a plateau at relatively low N inputs may be caused by sub-optimal management leading to low maximum attainable yields with increasing N application. Applicability of the generic N response for Africa remains a concern and needs additional work.

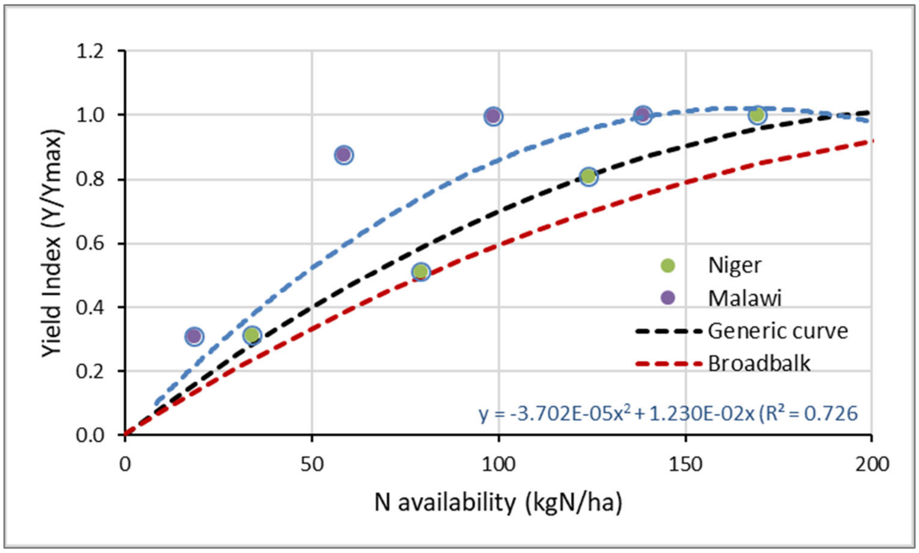

Fig. SI 14 Scaled N response in Niger and Malawi for maize*, as compared to scaled N response for global cereals and for winter wheat in Rothamsted*

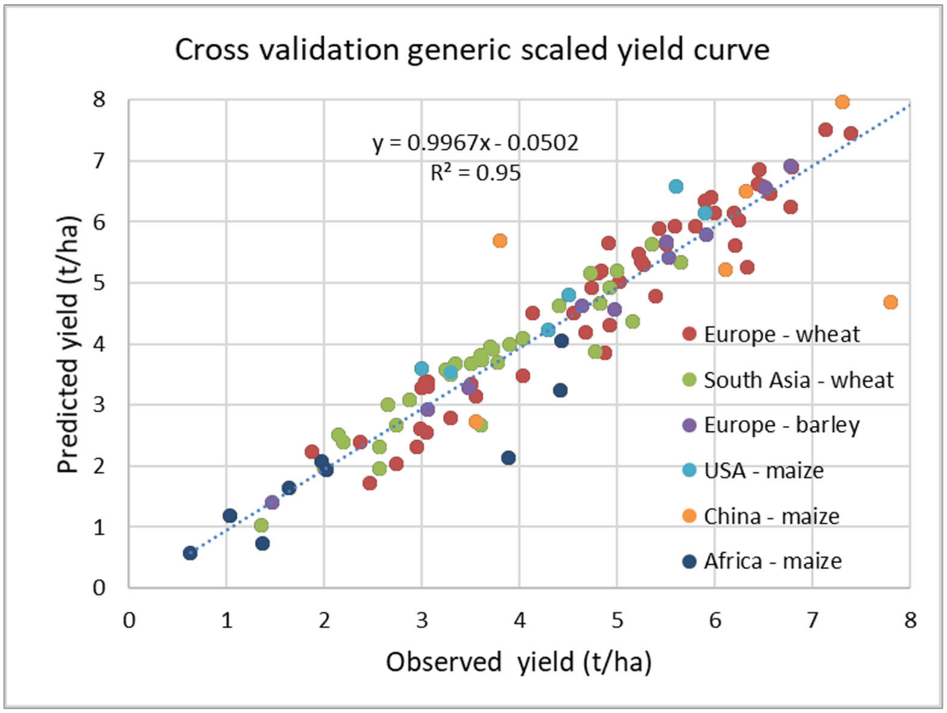

Fig. SI 15 Cross validation of scaled yield response curve for global cereals including maize in Africa*, where unscaled yields per site and applied N rate are back calculated using the 2nd order polynomial describing the indexed yield as function of total N rate.*

Additional references

Conant, R. T., Berdanier, A. B., & Grace, P. R. (2013). Patterns and trends in nitrogen use and nitrogen recovery efficiency in world agriculture. Global Biogeochemical Cycles, 27(2), 558-566.

Fandika, I. R., Zingore, S., Magreta, R., Chiipanthenga, M. & Ndlovu, W. in Annual Report-Soil and Agricultural Engineering Commodity Group, 2007/08 126-132 (Department of Agricultural Research Services Headquarters 2008).

Fertilizer Europe (2019). Forecast of food, farming and fertilizer use 2019-2029. VOLUME 2: Country data and national scenarios. Fertilizers Europe asbl, 9-31 Avenue des Nerviens, B-1040 Brussels

OFRA (Optimizing Fertilizer Recommendations in Africa), 2017. OFRA fertilizer trails. Available at: http://ec2-54-93-187-255.eu-central-1.compute.amazonaws.com/.

Pandey, R. K., Maranville, J. W. & Bako, Y. Nitrogen Fertilizer Response and Use Efficiency for Three Cereal Crops in Niger. Communications in Soil Science and Plant Analysis 32, 1465-1482, doi:10.1081/css-100104206 (2007).

Velthof, G. L., Oudendag, D., Witzke, H. P., Asman, W. A. H., Klimont, Z., & Oenema, O. (2009). Integrated assessment of nitrogen losses from agriculture in EU-27 using MITERRA-EUROPE. Journal of Environmental Quality, 38(2), 402-417.

Wen, Z. et al. Combined Applications of Nitrogen and Phosphorus Fertilizers with Manure Increase Maize Yield and Nutrient Uptake via Stimulating Root Growth in a Long-Term Experiment. Pedosphere 26, 62-73, doi:10.1016/s1002-0160(15)60023-6 (2016).

Note 7 Predicting long-term N response from short-term field trials

We analyzed an extensive, complete and publicly available dataset for short term maize trials in Nebraska (Wortman et al, 2017; https://datadryad.org/stash/dataset/doi:10.5061/dryad.p30c6). For most year-site combinations in this data set, soil N supply was very high and N response therefore relatively weak. This renders the data unsuitable for extrapolation towards an intercept on the 'available N' axis, to estimate *SN* and convert the *Nrate* to *Nav*. However, analysis of these short-term response trials confirmed our main concept of differences between short-term N response with a substantial part of N removal by crop from the soil N pool and long-term N response, where soil N is close to steady state.

We used two approaches to compare the yield response to N input observed in short-term and long-term field trials

(1) regression analysis with *AE* based on unscaled observations and

(2) regression analysis with *Yr* based on scaled observations

We could fit a regression model for agronomic efficiency (AE, kg grain per kg of added N, as we did earlier for LTEs (Figure 5 and Eq. 3 in main text). The advantage of interpreting the STE data via a regression model for AE is that it allows to reset the data to a common and low Y_0 values (yield at zero input of N fertilizer), to replace the very high Y_0 found in most of the site x year combinations of the Nebraska set. (Those high values make a direct comparison with our LTE data impossible, as Y_0 is much smaller in the LTEs). The regression model for AE in the Nebraska set has Y_0 as an extra regressor.

$$AE = -0.9 + 9.74 \times Nrate - 0.0063 \times Ymax - 9.74 \times Y_0 - 0.0201 \times (Nrate \times Ymax) + 0.0211 \times (Nrate \times Y_0)$$

(Eq. SI 6)

The new model fits equally well to the Nebraska STEs with R^2 0.87, as did our AE model to the LTEs (see Fig. 5, main paper).

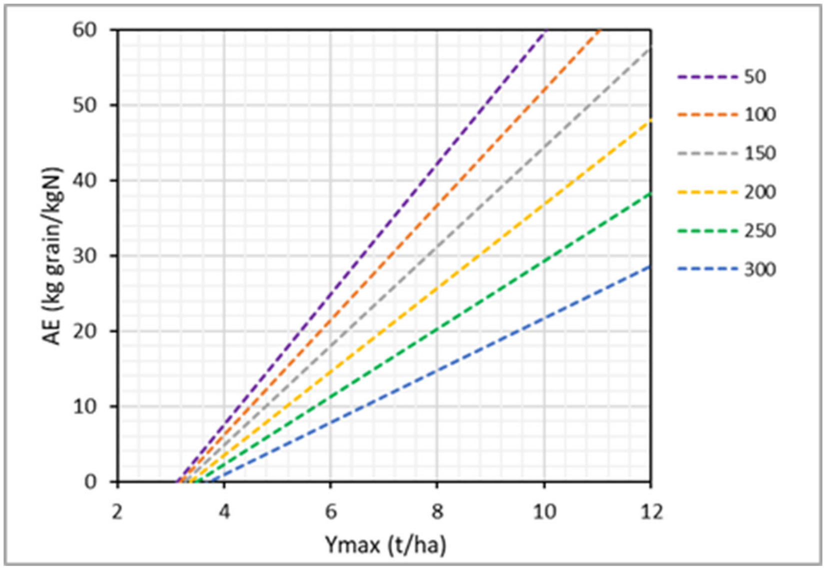

Fig SI 16 Agronomic Efficiency (AE) for maize in Nebraska derived from ST trials. *AE is fitted to observations as function of N rate (Eq. SI 6), Ymax and* Y_0*, for 34 STEs: AE is calculated for an* Y_0 *of 3 t/ha which is the average of the observed* Y_0 *for our 25 LT global trials.*

We compared the scaled N response for the Nebraska dataset to our generic N response function for global cereals, applying regression analysis for two response models, one with AE and one with *Yr*

1. *AE(Ymax, Yo, Nrate)*
   AE = $a + b \times Nrate + c \times Ymax + d \times Y_0 + e \times (Nrate \times Ymax) + f \times (Nrate \times Y_0)$ (Eq. SI 7)
   which can be rewritten to
   $Yr = ((AE \times Nrate) + Yo)/Ymax$, (Eq. SI 8)

   after substitution of AE by the regression model with *Nrate* the resulting function is also a quadratic relation with *Nrate* (and with intercept > 0)

2. *Yr(Nav) with Nav=Nrate + SN gives*
   $Yr = a \times (Nrate + SN) + b \times (Nrate + SN)^2$ (Eq. SI 9)

(1) regression analysis with AE based on unscaled observations

We applied the AE model for the Nebraska trials for a range of *Ymax* values and a *Yo* of 3 t/ha, corresponding to the mean *Yo* as found for the 25 LT global cereal trials. Actual *Ymax* in the Nebraska trials ranged between 12 and 18 t/ha, and *Yo* ranged between 6 and 13 t/ha (Table 2; Dobermann et al., 2011)[43]. We ran our generic N function for global cereals using a *SN* value of 30 kgN/ha to produce a LT relative *Yo* of 0.25 *Ymax* in Nebraska as found in the STEs. For *Ymax* values between 8 and 16 t/ha, representative for Nebraska, we find a good match between the scaled N response curve for Nebraska maize and our generic N function (see Figure A below). This suggests that the AE model for Nebraska indeed can be used to predict long term N response by adjusting *Yo* to observed values in LTEs (the used *Yo* of 3 t/ha is much lower than the observed ST *Yo* in Nebraska). The AE model for maize in Nebraska appears not to be applicable for *Ymax* values below 8 t/ha beyond N rates higher than 100 kgN/ha. Such conditions e.g. apply to maize in Asia and Latin America.

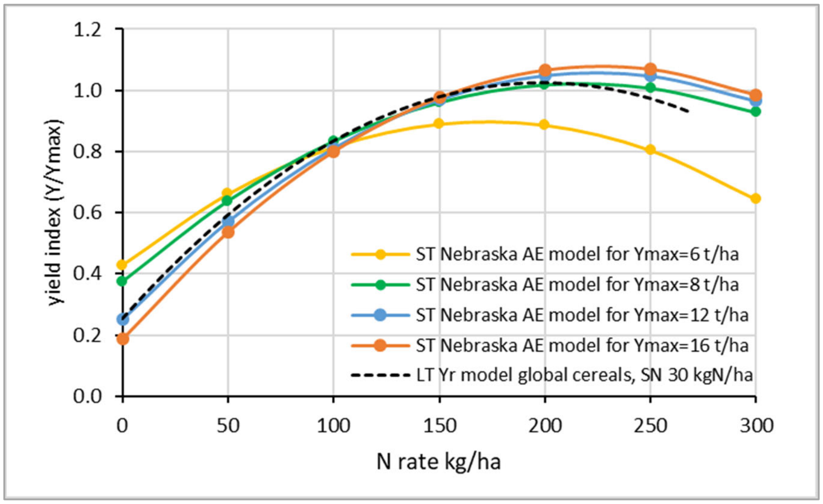

***Fig SI 17 Yield index by generic scaled LT N response function for global cereals as compared to yield index predicted by ST AE model for maize in Nebraska** (Eq. SI 9) using a SN value of 30 kgN/ha with (A.) results of the newly derived AE model for ST N response in Nebraska using a Yo of 3 t/ha and for Ymax ranging between 7 and 16 t/ha*

The AE model for our 25 global LTEs, which does not use *Yo*, can also be used to construct the long-term relation between *Yr* and N input (Figure SI 18) and gives a wider range of *Yr*(N rate) relations as it does not account for the variation of *Yo* in the 25 long-term trials; *Yo/Ymax* ranged between 0.26 and 0.78 (mean 0.48) as compared to between 0.45 and 0.99 (mean 0.71) for the Nebraska maize trials.

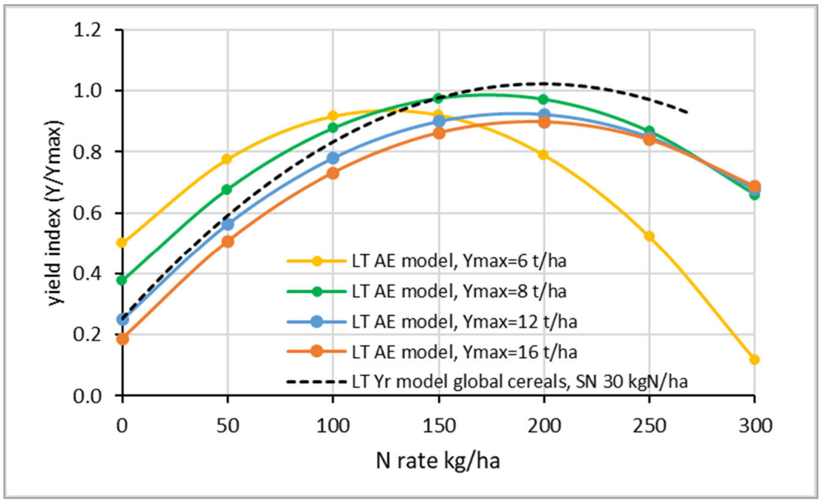

Fig SI 18 Yield index by generic scaled LT N response function for global cereals as compared to yield index predicted by LT AE model *(see Eq. 3 and Figure 5 in min paper). LT AE model no function of Yo while ST AE model is.*

(2) regression analysis with Yr based on scaled observations

In a second approach to compare long-term and short-term N response curves we selected eight trials from the total number of 34 (short-term) trials in the of Nebraska dataset with *SN* < 150 kgN/ha as an indication that N delivery form the soil N pool was not too high. This *SN* criterion was less strict than used for the LTEs in the main manuscript. Next, we scaled the Nebraska observations as for the LTEs and fitted a 2nd order polynomial with zero intercept on the selection of 8 trials with 216 observations. While the quadratic fit is quite good (R^2=0.714) the N response is weaker than for the 25 LTEs as was found for winter wheat in the UK comparing Broadbalk to first year N response in short-term trials (see Figure 1 in original submission and a scaled version below).

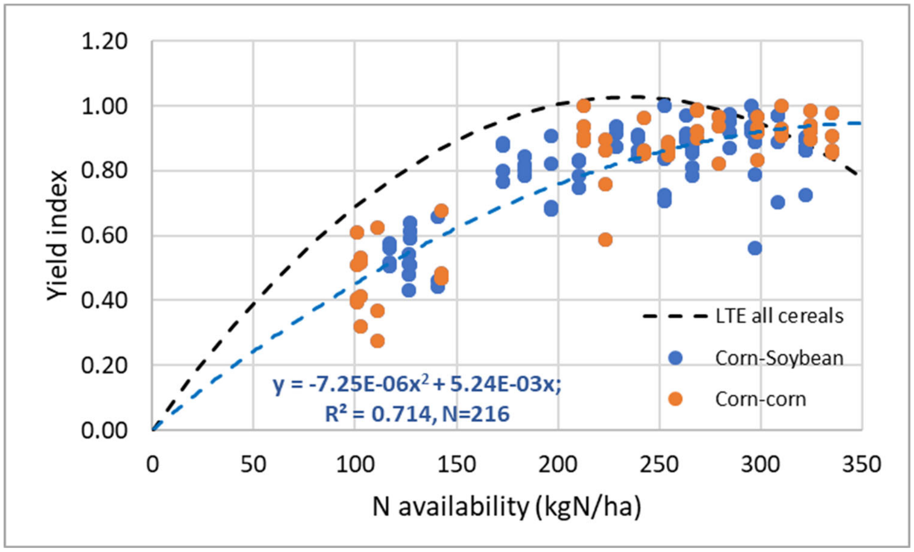

Fig SI 19 Scaled N response for short-term maize yield response to N rate in Nebraska as compared to scaled N response derived for 25 long-term trials *for maize, wheat and Barely (see Fig. 4 main text)). Results confirm that N response in short term trials is weaker (AE is lower) than in long-term trials because of the effect on net N soil delivery caused by build-up of soil fertility in higher fertilized plots.*

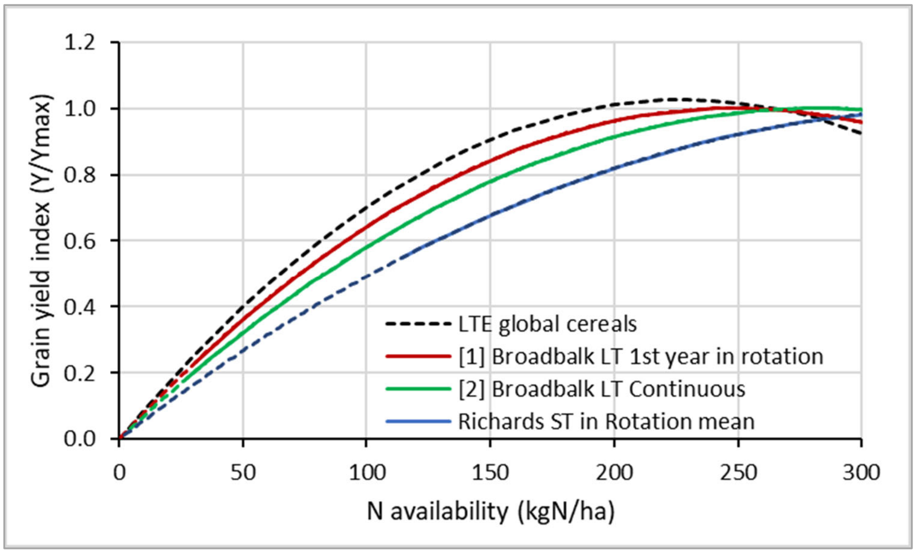

Fig SI 20 Scaled long-term and short-term N response for wheat in the UK *showing that ST response is weaker than LT.*

We conclude that the analysis of the Nebraska STEs confirms that LT response of grain yield to fertilizer N is steeper than in STEs, due to exhaustion of the control plot and build-up of soil fertility in fertilized plots, at least relative to the unfertilized control. Global application of the ST AE model to predict LT N response, as needed for sustainability analysis, would require knowledge of regional values for *Yo* which in general are not available.

Additional references

Wortmann, C. S., Tarkalson, D. D., Shapiro, C. A., Dobermann, A. R., Ferguson, R. B., Hergert, G. W., & Walters, D. (2011). Nitrogen use efficiency of irrigated corn for three cropping systems in Nebraska. Agronomy Journal, 103(1), 76-84.

Wortmann, C. S. et al. (2017), Data from: High-yielding corn response to applied phosphorus, potassium, and sulfur in Nebraska, Dryad, Dataset, https://doi.org/10.5061/dryad.p30c6.

Note 8 Generic long-term N-response curve for paddy irrigated rice in Asia

For the case of rice four long term N trials were found for South Asia, two for India (Bhandari et al., 2002; Majumber et al., 2008) and two for Nepal (Gami, et al., 2001; Rawal et al., 2017)). The curves apply to cropping system with irrigated (paddy) rice in summer followed by wheat in winter, which constitutes the most widely adopted cereal production system in South Asia. Wheat contributed about one third to the total annual cereal yield. In spite of difference in climate and rainfall (amount and distribution), genotype, soil type, and P and K status and fertilization and use of FYM, yields are indexed to the maximum yield of the four sites, the LT N response curves for the four experiments could be fitted quite well by a 2nd order polynomial (Fig. SI 12b). The uncertainty in the long-term yield at zero N input is largest, which is understandable. The yield at zero N fertilizer input depends most on soil type and history and the duration of the experiment (typically about 15-25 years). The best fit is obtained when plotting the cumulative yield of rice and wheat against the total mineral N input for both crops.

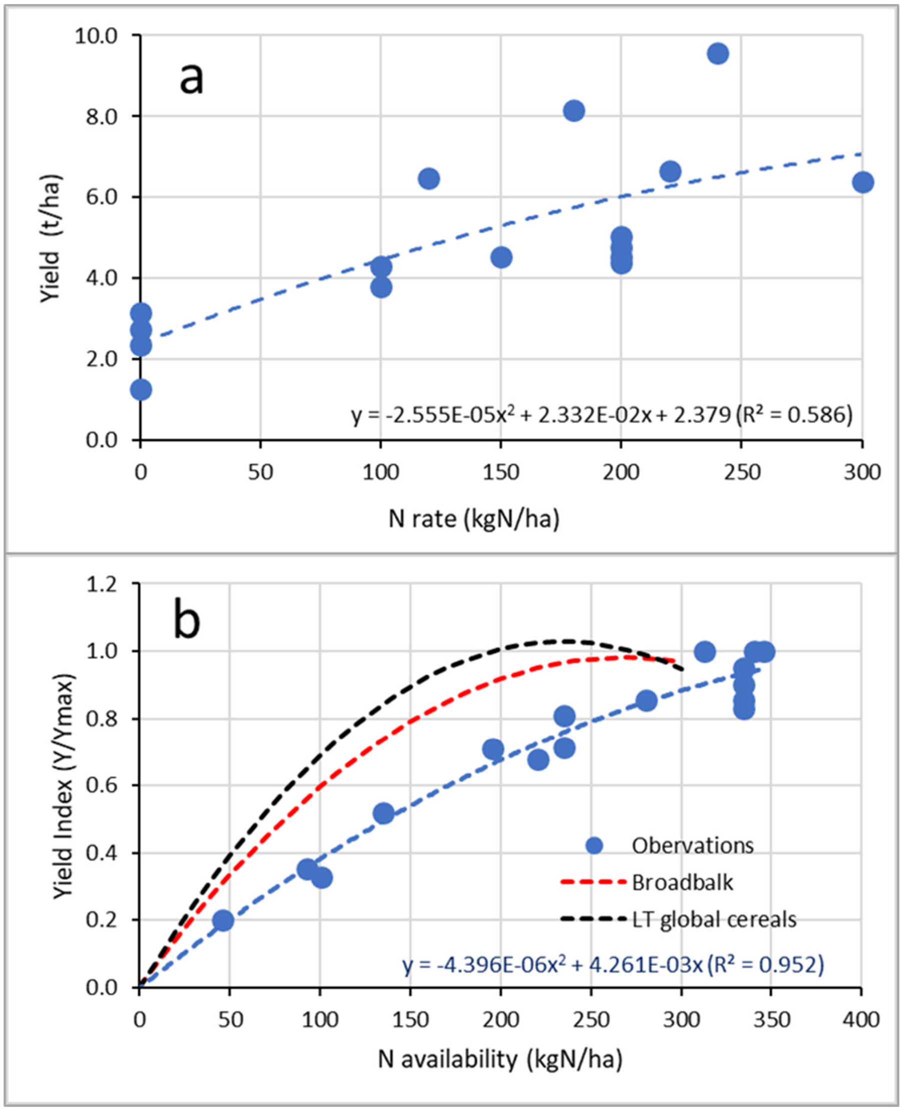

***Fig SI 21 Unscaled (a) and scaled (b) long-term N response data for rice-wheat** two-crop systems as observed in two long term trials in India and two in Nepal (a) and 2nd order polynomial fit with zero intercept, as compared to the scaled N response for global cereals and for winter wheat in rotation Broadbalk (b).*

Rice+Wheat: *Yr =* $-4.396E\text{-}6 \times Nav^2 + 4.261E\text{-}3 \times Nav$ (Eq. SI 10, R^2 0.952; N=16)

	Value	*Stand. Error*	*t Stat*	*P-value*	*Lower 95%*	*Upper 95%*
Coefficient 1	4.261E-03	2.972E-04	1.434E+01	9.227E-10	3.624E-03	4.898E-03
Coefficient 2	-4.396E-06	9.640E-07	-4.560E+00	4.451E-04	-6.464E-06	-2.328E-06

The number of four sites is very limited and cover a small part of Asia. In view of its specific agro-climatic conditions, hydrology and growing systems (see for example Cassman et al., 1998) more data and further analysis is needed to derive a generic LT N response curve for rice.

Additional references

Bhandari, A. L. et al. Yield and Soil Nutrient Changes in a Long-Term Rice-Wheat Rotation in India. Soil Science Society of America Journal 66, 162-170 (2002).

Cassman, K. G., Peng, S., Olk, D. C., Ladha, J. K., Reichardt, W., Dobermann, A., & Singh, U. (1998). Opportunities for increased nitrogen-use efficiency from improved resource management in irrigated rice systems. Field crops research, 56(1-2), 7-39.

Gami, S. et al. Long-term changes in yield and soil fertility in a twenty-year rice-wheat experiment in Nepal. Biology and Fertility of Soils 34, 73-78, doi:10.1007/s003740100377 (2001).

Majumder, B. et al. Organic Amendments Influence Soil Organic Carbon Pools and Rice-Wheat Productivity. Soil Science Society of America Journal 72, 775-785, doi:10.2136/sssaj2006.0378 (2008).

Rawal, N., Ghimire, R. & Chalise, D. Crop Yield and Soil Fertility Status of Long-Term Rice-Rice-Wheat Cropping Systems. International Journal of Applied Sciences and Biotechnology 5, 42-50, doi:10.3126/ijasbt.v5i1.17001 (2017).

Note 9 Crop and soil nitrogen response to total N availability for Winter wheat at Broadbalk and implications for *NUE*

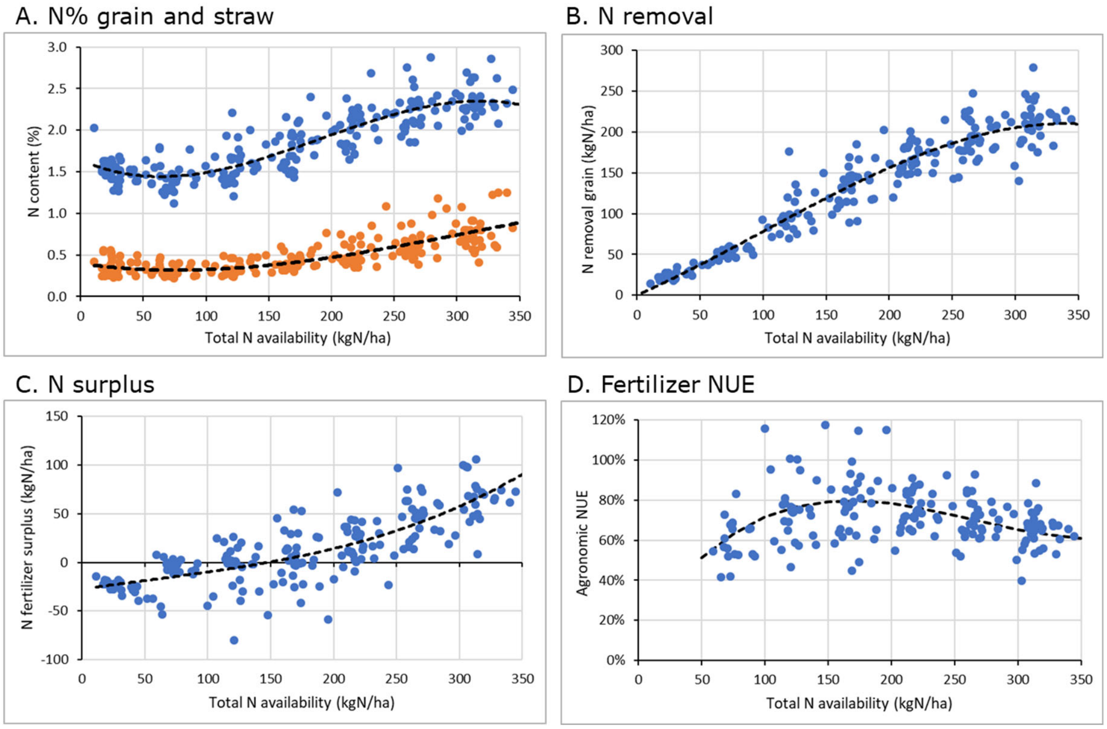

Fig. SI 22 Crop and soil nitrogen response to total N availability for Winter wheat *in rotation at Broadbalk for the period 1985-2016 and effect on agronomic nitrogen use efficiency.*

A linear model of N% with *Ymax* and Nav was fitted to observations between 1985 and 2016

N% = 1.873 + 3.26E-3 x Nav – 6.20E-2 x Ymax (Eq. SI 11, R^2 = 0.743, N=224)

	Value	*Stand. Error*	*t Stat*	*P-value*	*Lower 95%*	*Upper 95%*
Intercept	1.873E+00	1.055E-01	1.776E+01	1.899E-44	1.666E+00	2.081E+00
Coefficient 1	3.259E-03	1.331E-04	2.449E+01	6.567E-65	2.997E-03	3.522E-03
Coefficient 2	-6.198E-02	1.097E-02	-5.649E+00	4.945E-08	-8.361E-02	-4.036E-02

Implications for NUE

When the N%(Nav) relation is combined with the relation between *Yr(Nav)* N surplus can be calculated as:

Nsurplus = Nav – Nremoval (Eq. 8, main paper)

Below stepwise results are shown when applied for Broadbalk wheat in rotation as function of Nav and *Ymax*:

$Yr = -1.354E\text{-}5 \times Nav^2 + 7.291E\text{-}3 \times Nav$ (Eq. 1 main paper R^2 0.954)

**N%**							
	Nav						
Ymax	25	75	125	175	225	275	325
3	1.77	1.93	2.09	2.26	2.42	2.58	2.75
4	1.71	1.87	2.03	2.20	2.36	2.52	2.68
5	1.64	1.81	1.97	2.13	2.30	2.46	2.62
6	1.58	1.75	1.91	2.07	2.23	2.40	2.56
7	1.52	1.68	1.85	2.01	2.17	2.34	2.50
8	1.46	1.62	1.78	1.95	2.11	2.27	2.44
9	1.40	1.56	1.72	1.89	2.05	2.21	2.37
10	1.34	1.50	1.66	1.82	1.99	2.15	2.31
**Yield (t/ha)**							
	Nav						
Ymax	25	75	125	175	225	275	325
3	0.64	1.69	2.44	2.90	3.07	2.94	2.51
4	0.85	2.25	3.26	3.87	4.09	3.92	3.35
5	1.06	2.81	4.07	4.84	5.12	4.90	4.19
6	1.27	3.38	4.89	5.81	6.14	5.88	5.03
7	1.49	3.94	5.70	6.78	7.16	6.86	5.86
8	1.70	4.50	6.52	7.75	8.19	7.84	6.70
9	1.91	5.06	7.33	8.71	9.21	8.82	7.54
10	2.12	5.63	8.15	9.68	10.23	9.80	8.38
**N-removal grain (kgN/ha)**							
	Nav						
Ymax	25	75	125	175	225	275	325
3	11	33	51	66	74	76	69
4	14	42	66	85	97	99	90
5	17	51	80	103	118	120	110
6	20	59	93	120	137	141	129
7	23	66	105	136	156	160	147
8	25	73	116	151	173	178	163
9	27	79	126	164	189	195	179
10	28	84	135	177	203	211	194
**NUE grain**							
	Nav						
Ymax	25	75	125	175	225	275	325
3	45%	43%	41%	37%	33%	28%	21%
4	58%	56%	53%	49%	43%	36%	28%
5	70%	68%	64%	59%	52%	44%	34%
6	81%	79%	75%	69%	61%	51%	40%
7	90%	88%	84%	78%	69%	58%	45%
8	99%	97%	93%	86%	77%	65%	50%
9	107%	105%	101%	94%	84%	71%	55%
10	113%	112%	108%	101%	90%	77%	60%

**N surplus kgN/ha (exl. straw removal)**							
	Nav						
Ymax	25	75	125	175	225	275	325
3	14	42	74	109	151	199	256
4	11	33	59	90	128	176	235
5	8	24	45	72	107	155	215
6	5	16	32	55	88	134	196
7	2	9	20	39	69	115	178
8	0	2	9	24	52	97	162
9	-2	-4	-1	11	36	80	146
10	-3	-9	-10	-2	22	64	131

Table SI 4 Effects of N availability (Nav) and maximum attainable grain yield (Ymax) on N balance and NUE for wheat in the UK *derived* ***from*** *generic relations for long-term effect of Nav on relative grain yield (Eq. 3 main paper) and on N percentage in grain (Eq. SI 11).*

NUE is calculated here as the ratio of N removal by grain and total N input. *NUE* values exceeding 80% are unrealistic in view of inevitable gaseous N losses. Values exceeding 100% further imply net soil N depletion (so a negative N surplus) and indicate that the statistical models are applied too far beyond the range of observations.

Note 10 Optimal N fertilizer rates for farm and society; concept and illustrative results for the Netherlands

Farmers need insight into the marginal response of yield to N rate to determine the economic optimal N rate. Marginal response depends on the time horizon of optimization and the choice of response curve. The net economic return is the gross return from crop sales minus the fixed and variable costs of farm and contracted labor, capital and N fertilizer inputs, for which the price per unit of grain and N fertilizer are required. Two relevant parameters for farming are the minimum N input to generate a net financial benefit, and the N input for which the financial benefit is at its maximum.

The derivation of optimal yields depends on inflection points, and therefore on the slope of yield – N rate relation. At a typical N rate for cereals of 150 kgN/ha the slope of the yield response for LT N response curves at Rothamsted is almost twice as high as those for 1-2 year commercial trials (Richards et al., 2000)

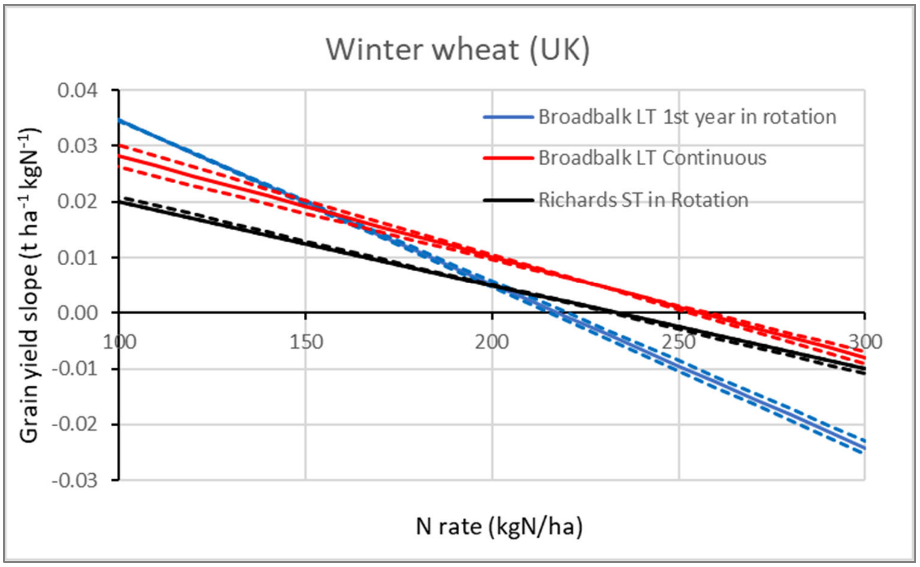

Fig SI 23 Marginal long- and short term N response for winter wheat in the UK*: (1. Blue) Long term N response for winter wheat in rotation, (2. Red) Long term N response for winter wheat in continuous cultivation at Broadbalk and (3. Black) the classical 1st year N response for winter wheat in rotation (Richards et al., 2000) (dashed lines, 95% Confidence Intervals).*

The graphs below illustrate the derivation of the range of sustainable N rates for farming and society for cultivation of winter wheat in the Netherlands. The curve for the net N benefits for society is obtained by subtracting the external cost from the net N benefit curve at farm level. In this example, the price ratio adjustment to reflect what consumers actually pay for food is not made Accounting for external cost of N pollution narrows the range of N rates that deliver robust net benefits as compared to farm scale.

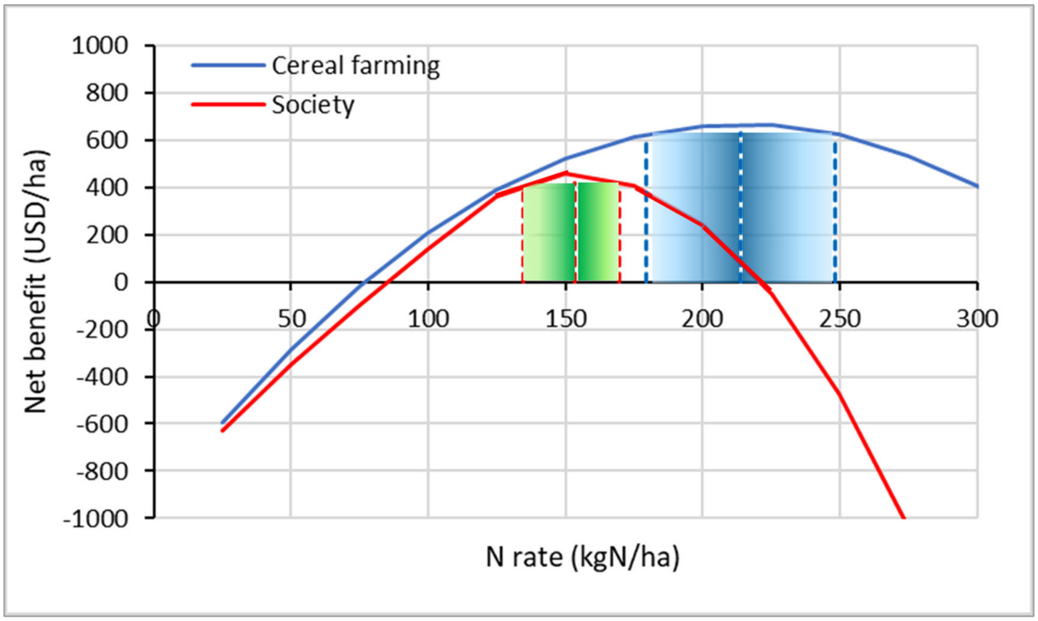

Fig SI 24 Illustration of safe range of N inputs, *with maximum economic benefits for cereal farming (blue line and range) and for society (red line and green range). Input data for cultivation of winter wheat in the Netherlands (Ymax = 9 t/ha, GDP 50 kUSD/cap, N price 1 USD/kgN, wheat grain price farm gate and food plate both 0.2 USD/kg) and the N response as for Broadbalk; fixed and variable cost for Dutch arable agriculture were taken from* https://www.wur.nl/nl/show/kwin-agv.htm*).*

For northwestern Europe the price of N fertilizer is around 1 USD/kgN, and of wheat grain 0.2 USD/kg[11]. We assume that the N response curves and N budgets for Winter wheat in the UK are also applicable for the Netherlands, in view of similar climate and *Ymax* values of around 9 t/ha and took into account that in the Netherlands 60% of N is applied as manure[24].

The range of mineral N rates that ensures a net (>0 USD/ha) to maximum financial return for wheat in rotation at farm scale in the Netherlands would have been 14-233 (range A: 219) kgN/ha when using the 1st year N response curve (not shown), narrowing to 61-218 (range B: 157; 70% of range A) kgN/ha. When also considering the external cost of N pollution[13,44] the range of N rates with both LT net benefits for farm and society further narrows to 45-135 (range C; 40% of range A) kgN/ha. The current N rate of 165 kgN/ha exceeds the upper bound of range C (135 kgN/ha) which implies net costs for society as the cost of N pollution exceeds the net yield benefit for the farmer.

Note 11 External cost of N surplus in the European Union

	GDP	N surplus	Total N cost	Unit cost
	2010-2014	2008	2008	
	current kUSD/cap	GgN/yr	Geuro/yr	Euro/kgN surplus
Austria	49.5	95.3	1.26	13.2
Belgium	46.3	171.8	4.09	23.8
Bulgaria	7.3	164.3	0.39	2.4
Cyprus	29.2	21.1	0.18	8.8
Czech Republic	20.1	197.9	2.59	13.1
Denmark	59.4	160.3	6.91	43.1
Estonia	17.5	28.0	0.10	3.6
Finland	48.7	180.1	1.48	8.2
France	42.1	1645.3	23.10	14.0
Germany	45.1	1073.5	20.24	18.9
Greece	23.8	290.3	5.77	19.9
Hungary	13.4	198.3	1.39	7.0
Ireland	50.4	389.8	4.06	10.4
Italy	35.9	759.1	14.64	19.3
Latvia	14.1	65.7	0.19	2.8
Lithuania	14.6	105.1	0.21	2.0
Luxembourg	108.3	16.1	0.46	28.4
Malta	21.5	3.0	0.04	13.6
Netherlands	51.1	336.9	7.62	22.6
Poland	13.5	1557.0	7.21	4.6
Portugal	22.0	164.0	1.47	8.9
Romania	9.0	319.5	1.26	3.9
Slovak Republic	17.6	71.2	0.65	9.2
Slovenia	23.6	35.2	0.49	13.9
Spain	30.4	1014.9	11.51	11.3
Sweden	57.6	110.5	4.57	41.4
United Kingdom	41.6	943.9	16.57	17.6

***Table SI 5 N surplus and N damage cost data for agriculture in the EU27 in 2008** used to derive the relationship between GDP per capita and the unit damage cost per kg N surplus. Differences in unit damage cost reflect differences in (a) sources and application techniques of fertilizer (incl. manure), (2) fates and impacts of N losses and (3) Willingness to pay (WTP) to prevent these impacts (data taken from Grinsven et al., 2018[44]; see also for further details on methods))*

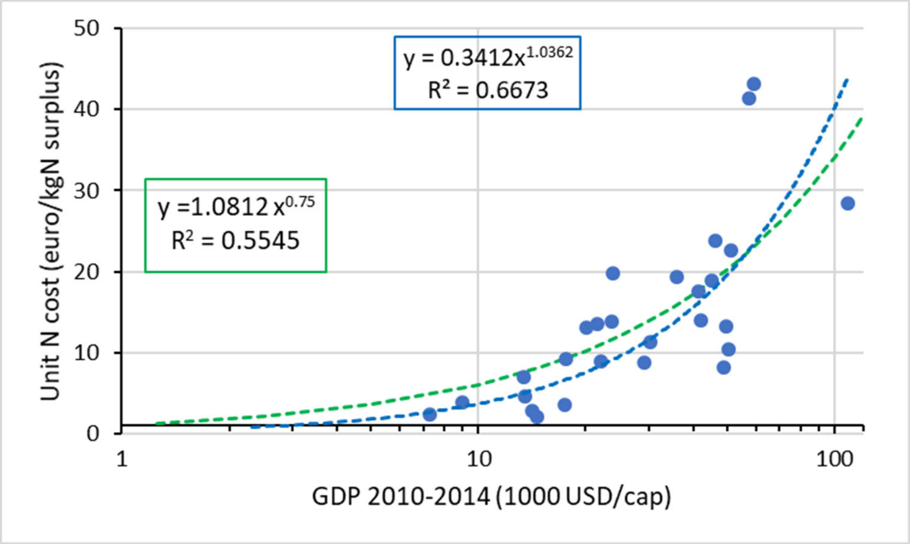

Figure SI 25 Relationship between GDP and the unit cost per kg N surplus for EU27 around 2010 *(based on data in* ***Table SI 3.*** *and van Grinsven et al., 2018[44]). The exponent of the power relation between Unit costs and GDP can be viewed as an income elasticity. Fitting a power function (linear regression on log transformed GDP and Unit costs) gives an elasticity of 1.036, and therefore almost a linear relation between Unit Costs and GDP. This function was used to estimate unit costs for countries outside the EU27, with GDP in developing countries up to one order of magnitude lower than in poorest EU27 member state (Bulgaria). For illustration also a fit with a power function with an income elasticity of 0.75 is shown, which gives a poorer fit (0.554 versus 0.667) for log-transformed data but a better fit on untransformed data (R2 0.530 for elasticity 0.75 as compared to 0.481 for elasticity 1.036). The model an elasticity of 0.75 predicts higher Units costs for countries with a GDP below 50 kUSD/cap, amounting to a factor of 2 at GDP 5 kUSD/cap and a factor of 3 at GDP 1 kUSD cap.*

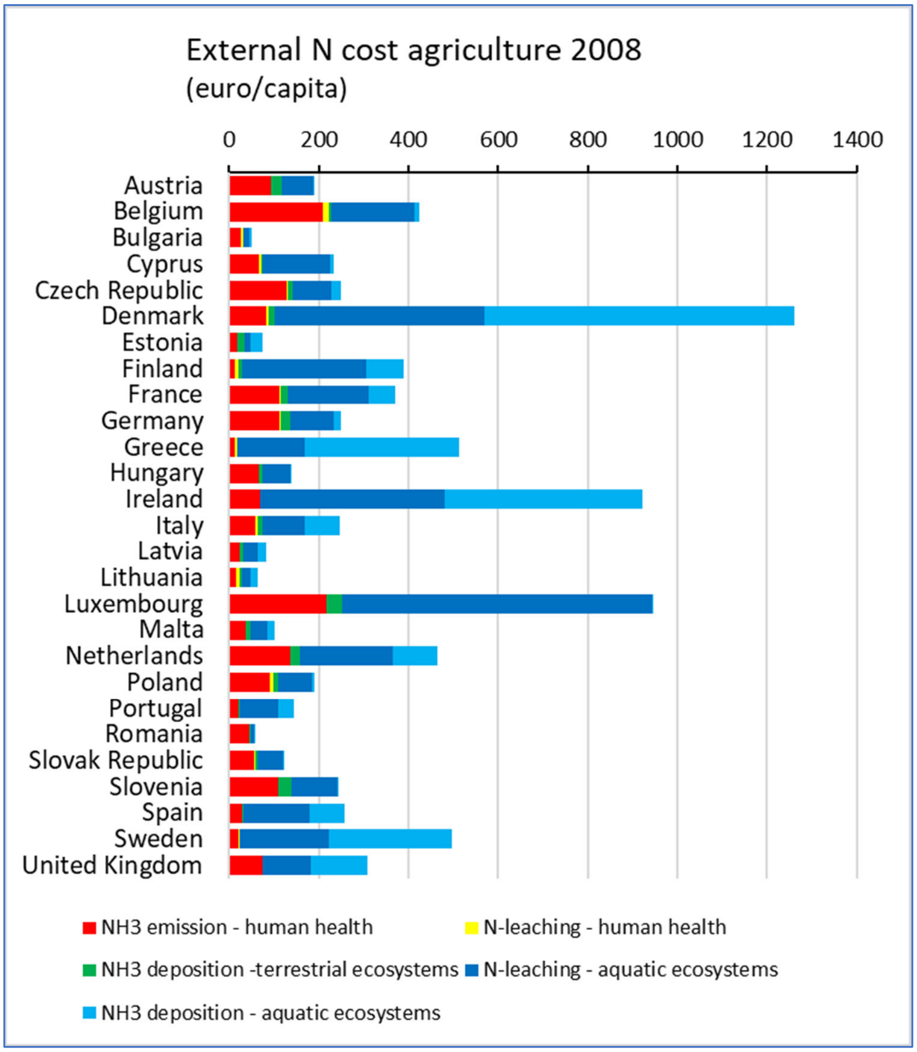

***Fig SI 26 External cost by N pollution from agriculture in the EU27 in 2008 per capita** and breakdown to the most relevant impact mechanisms. Countries with relatively high costs for human health from NH_3 containing aerosols often have relatively high shares of manure N in total N fertilizer input. Countries with high cost by aquatic impacts of NH3-N deposition are countries with large marine territories. For details see Van Grinsven et al., (2013)[13] and (2018)[44].*

The food plate equivalent price of cereals (FPP) is needed to determine the SONR and is underestimated by using the farm price (FGP). The ratio of FPP over FGP was approximated in two ways (See Supplementary Note 13).

Farm level

Netherlands: GDP=51100 USD/cap

Net farm benefit USD/ha

Ymax/Nrate	25	50	75	100	125	150	175	200	225	250	275	300	325	350
2.0	-915	-865	-825	-795	-774	-764	-763	-772	-791	-820	-858	-907	-965	-1033
2.5	-894	-825	-769	-725	-693	-673	-666	-671	-688	-718	-760	-815	-881	-960
3.0	-873	-785	-712	-654	-611	-583	-569	-570	-586	-617	-662	-722	-797	-887
3.5	-851	-745	-656	-584	-530	-492	-472	-469	-484	-515	-564	-630	-714	-814
4.0	-830	-705	-600	-514	-448	-402	-375	-369	-381	-414	-466	-538	-630	-741
4.5	-809	-665	-544	-444	-367	-312	-279	-268	-279	-313	-368	-446	-546	-668
5.0	-788	-625	-487	-374	-285	-221	-182	-167	-177	-211	-270	-354	-462	-595
5.5	-767	-585	-431	-304	-204	-131	-85	-66	-74	-110	-172	-262	-379	-522
6.0	-745	-545	-375	-234	-122	-41	12	35	28	-9	-74	-170	-295	-449
6.5	-724	-505	-319	-164	-41	50	109	135	130	93	24	-78	-211	-376
7.0	-703	-465	-262	-94	41	140	205	236	232	194	122	14	-127	-303
7.5	-682	-425	-206	-24	122	231	302	337	335	296	220	107	-43	-230
8.0	-661	-385	-150	47	204	321	399	438	437	397	318	199	40	-157
8.5	-639	-345	-93	117	285	411	496	539	539	498	415	291	124	-84
9.0	-618	-305	-37	187	366	502	593	639	642	600	513	383	208	-11
9.5	-597	-265	19	257	448	592	690	740	744	701	611	475	292	62
10.0	-576	-226	75	327	529	683	786	841	846	803	709	567	375	135
10.5	-554	-186	132	397	611	773	883	942	949	904	807	659	459	207
11.0	-533	-146	188	467	692	863	980	1043	1051	1005	905	751	543	280
11.5	-512	-106	244	537	774	954	1077	1143	1153	1107	1003	843	627	353
12.0	-491	-66	300	607	855	1044	1174	1244	1256	1208	1101	935	710	426

National society level

CP=0.2; FP=1; FC=1000; EC=20

Net societal benefit USD/ha

Ymax/Nrate	25	50	75	100	125	150	175	200	225	250	275	300	325	350
2.0	-1261	-1563	-1881	-2219	-2580	-2965	-3377	-3818	-4291	-4798	-5342	-5926	-6550	-7219
2.5	-1204	-1452	-1721	-2014	-2334	-2685	-3069	-3490	-3949	-4452	-4999	-5595	-6243	-6945
3.0	-1149	-1344	-1564	-1813	-2094	-2411	-2768	-3168	-3614	-4111	-4662	-5271	-5941	-6676
3.5	-1094	-1238	-1411	-1616	-1859	-2143	-2473	-2852	-3285	-3777	-4331	-4952	-5644	-6412
4.0	-1041	-1135	-1261	-1424	-1629	-1880	-2183	-2542	-2963	-3449	-4006	-4639	-5353	-6152
4.5	-990	-1035	-1115	-1236	-1404	-1623	-1900	-2239	-2647	-3128	-3688	-4332	-5067	-5896
5.0	-939	-937	-972	-1053	-1184	-1372	-1623	-1942	-2337	-2812	-3375	-4031	-4786	-5646
5.5	-890	-841	-833	-874	-969	-1126	-1351	-1652	-2034	-2504	-3069	-3735	-4510	-5399
6.0	-843	-748	-697	-699	-759	-886	-1086	-1367	-1737	-2201	-2768	-3445	-4239	-5158
6.5	-796	-657	-565	-528	-554	-651	-827	-1089	-1446	-1905	-2474	-3161	-3974	-4920
7.0	-751	-569	-437	-362	-355	-423	-574	-817	-1162	-1615	-2186	-2882	-3714	-4688
7.5	-708	-483	-311	-201	-160	-199	-327	-552	-884	-1331	-1903	-2610	-3459	-4460
8.0	-665	-400	-190	-43	29	18	-86	-293	-612	-1054	-1627	-2343	-3209	-4236
8.5	-654	-371	-115	110	214	230	149	-40	-347	-783	-1358	-2081	-2964	-4017
9.0	-652	-370	-118	115	340	437	378	207	-88	-518	-1094	-1826	-2725	-3803
9.5	-648	-365	-116	111	328	547	601	447	164	-260	-836	-1576	-2491	-3593
10.0	-642	-359	-111	112	322	532	754	682	410	-8	-584	-1332	-2262	-3388
10.5	-636	-349	-103	116	321	524	737	909	650	238	-339	-1093	-2039	-3187
11.0	-628	-338	-91	125	325	520	726	954	883	478	-99	-861	-1820	-2991
11.5	-619	-323	-76	139	334	523	721	941	1110	711	134	-634	-1607	-2799
12.0	-608	-307	-57	157	348	531	721	933	1181	937	361	-413	-1399	-2612

France: GDP=35400 USD/cap

Net farm benefit USD/ha

Ymax/Nrate	25	50	75	100	125	150	175	200	225	250	275	300	325	350
2.0	-599	-549	-508	-478	-458	-447	-446	-455	-474	-503	-542	-590	-648	-717
2.5	-577	-509	-452	-408	-376	-357	-349	-354	-372	-402	-444	-498	-565	-644
3.0	-556	-469	-396	-338	-295	-266	-253	-254	-270	-300	-346	-406	-481	-571
3.5	-535	-429	-340	-268	-213	-176	-156	-153	-167	-199	-248	-314	-397	-498
4.0	-514	-389	-283	-198	-132	-85	-59	-52	-65	-97	-150	-222	-313	-425
4.5	-493	-349	-227	-128	-50	5	38	49	37	4	-52	-130	-230	-352
5.0	-471	-309	-171	-57	31	95	135	150	140	105	46	-37	-146	-279
5.5	-450	-269	-115	13	113	186	232	250	242	207	144	55	-62	-206
6.0	-429	-229	-58	83	194	276	328	351	344	308	242	147	22	-133
6.5	-408	-189	-2	153	276	366	425	452	447	409	340	239	106	-60
7.0	-386	-149	54	223	357	457	522	553	549	511	438	331	189	13
7.5	-365	-109	111	293	439	547	619	654	651	612	536	423	273	86
8.0	-344	-69	167	363	520	638	716	754	754	714	634	515	357	159
8.5	-323	-29	223	433	601	728	812	855	856	815	732	607	441	232
9.0	-302	11	279	503	683	818	909	956	958	916	830	699	524	305
9.5	-280	51	336	573	764	909	1006	1057	1061	1018	928	791	608	378
10.0	-259	91	392	644	846	999	1103	1158	1163	1119	1026	884	692	451
10.5	-238	131	448	714	927	1089	1200	1258	1265	1220	1124	976	776	524
11.0	-217	171	504	784	1009	1180	1297	1359	1368	1322	1222	1068	859	597
11.5	-195	211	561	854	1090	1270	1393	1460	1470	1423	1320	1160	943	670
12.0	-174	251	617	924	1172	1361	1490	1561	1572	1525	1418	1252	1027	743

CP=0.2; FP=1; FC=680; EC=14

Net societal benefit USD/ha

Ymax/Nrate	25	50	75	100	125	150	175	200	225	250	275	300	325	350
2.0	-835	-1025	-1230	-1452	-1692	-1951	-2233	-2537	-2867	-3223	-3607	-4020	-4466	-4944
2.5	-790	-937	-1103	-1289	-1498	-1732	-1992	-2281	-2601	-2953	-3341	-3766	-4229	-4734
3.0	-745	-851	-978	-1130	-1308	-1516	-1755	-2029	-2339	-2689	-3080	-3515	-3996	-4527
3.5	-701	-766	-856	-973	-1122	-1304	-1523	-1781	-2082	-2428	-2822	-3268	-3767	-4323
4.0	-658	-683	-735	-820	-939	-1096	-1294	-1538	-1829	-2172	-2569	-3025	-3541	-4123
4.5	-616	-601	-618	-669	-759	-892	-1070	-1299	-1581	-1920	-2321	-2786	-3319	-3925
5.0	-575	-522	-502	-521	-583	-691	-850	-1064	-1337	-1673	-2076	-2551	-3101	-3731
5.5	-535	-444	-389	-377	-410	-495	-634	-833	-1097	-1429	-1835	-2319	-2886	-3539
6.0	-495	-367	-279	-235	-241	-302	-422	-607	-862	-1191	-1599	-2092	-2674	-3351
6.5	-457	-293	-171	-96	-75	-113	-214	-385	-631	-956	-1367	-1868	-2466	-3166
7.0	-420	-220	-65	39	87	72	-11	-167	-404	-726	-1139	-1649	-2262	-2984
7.5	-383	-148	39	172	246	253	189	46	-181	-500	-915	-1433	-2061	-2805
8.0	-347	-79	140	302	401	431	384	255	36	-278	-695	-1222	-1864	-2629
8.5	-333	-47	208	428	553	604	576	460	250	-61	-480	-1014	-1670	-2456
9.0	-325	-33	224	454	665	774	763	660	459	152	-268	-810	-1480	-2286
9.5	-315	-17	243	474	683	878	946	857	664	361	-61	-610	-1294	-2120
10.0	-305	0	264	496	704	896	1081	1049	865	565	142	-414	-1111	-1956
10.5	-294	19	288	522	729	919	1100	1236	1061	765	340	-222	-931	-1796
11.0	-281	40	314	550	758	945	1123	1299	1253	961	535	-34	-756	-1639
11.5	-268	62	342	581	789	976	1150	1321	1440	1153	726	150	-583	-1485
12.0	-254	86	373	616	825	1010	1181	1348	1521	1340	912	330	-415	-1334

Romania: GDP=9027 USD/cap

Net farm benefit USD/ha

Ymax/Nrate	25	50	75	100	125	150	175	200	225	250	275	300	325	350
2.0	-81	-31	9	39	60	71	71	62	43	15	-24	-73	-131	-199
2.5	-60	9	65	110	141	161	168	163	146	116	74	20	-47	-126
3.0	-39	49	122	180	223	251	265	264	248	217	172	112	37	-53
3.5	-17	89	178	250	304	342	362	365	350	319	270	204	120	20
4.0	4	129	234	320	386	432	459	465	453	420	368	296	204	93
4.5	25	169	290	390	467	522	555	566	555	521	466	388	288	166
5.0	46	209	347	460	549	613	652	667	657	623	564	480	372	239
5.5	67	249	403	530	630	703	749	768	760	724	662	572	455	312
6.0	89	289	459	600	712	794	846	869	862	826	760	664	539	385
6.5	110	329	516	670	793	884	943	969	964	927	858	756	623	458
7.0	131	369	572	740	875	974	1039	1070	1066	1028	956	848	707	531
7.5	152	409	628	811	956	1065	1136	1171	1169	1130	1054	941	791	604
8.0	174	449	684	881	1038	1155	1233	1272	1271	1231	1152	1033	874	677
8.5	195	489	741	951	1119	1245	1330	1373	1373	1332	1249	1125	958	750
9.0	216	529	797	1021	1200	1336	1427	1473	1476	1434	1347	1217	1042	823
9.5	237	569	853	1091	1282	1426	1524	1574	1578	1535	1445	1309	1126	896
10.0	258	609	909	1161	1363	1517	1620	1675	1680	1637	1543	1401	1209	969
10.5	280	648	966	1231	1445	1607	1717	1776	1783	1738	1641	1493	1293	1041
11.0	301	688	1022	1301	1526	1697	1814	1877	1885	1839	1739	1585	1377	1114
11.5	322	728	1078	1371	1608	1788	1911	1977	1987	1941	1837	1677	1461	1187
12.0	343	768	1134	1441	1689	1878	2008	2078	2090	2042	1935	1769	1544	1260

CP=0.2; FP=1; FC=108; EC=2.2

Net societal benefit USD/ha

Ymax/Nrate	25	50	75	100	125	150	175	200	225	250	275	300	325	350
2.0	-139	-147	-166	-197	-240	-295	-363	-443	-538	-646	-768	-906	-1058	-1226
2.5	-111	-95	-93	-105	-131	-173	-231	-305	-396	-504	-630	-774	-937	-1120
3.0	-84	-44	-20	-13	-23	-52	-100	-167	-255	-363	-492	-643	-817	-1014
3.5	-58	7	53	78	84	68	30	-31	-115	-223	-355	-514	-698	-909
4.0	-31	57	124	169	190	187	159	105	24	-84	-220	-385	-580	-805
4.5	-5	107	196	258	295	305	286	239	162	54	-85	-257	-462	-702
5.0	21	157	266	347	400	422	413	372	299	191	48	-130	-346	-600
5.5	47	206	336	436	503	538	539	505	434	327	181	-4	-230	-498
6.0	72	255	406	523	606	653	664	636	569	462	313	121	-115	-397
6.5	98	303	475	610	708	767	787	766	703	595	443	245	-2	-297
7.0	123	351	543	696	809	881	910	895	835	728	573	368	111	-197
7.5	148	399	611	781	909	993	1032	1023	967	860	701	490	224	-98
8.0	173	446	678	866	1009	1105	1153	1151	1097	990	829	611	335	0
8.5	192	484	737	950	1107	1215	1272	1277	1226	1120	955	731	445	97
9.0	210	518	784	1009	1196	1325	1391	1402	1355	1248	1081	850	555	193
9.5	229	552	831	1067	1262	1419	1509	1526	1482	1376	1205	969	664	289
10.0	247	586	878	1125	1329	1492	1615	1649	1608	1502	1329	1086	772	384
10.5	266	621	927	1185	1397	1566	1693	1770	1733	1627	1451	1202	879	478
11.0	285	657	976	1244	1465	1640	1772	1862	1857	1752	1573	1318	985	572
11.5	304	692	1025	1305	1535	1716	1852	1944	1980	1875	1693	1432	1090	664
12.0	324	728	1075	1367	1605	1793	1933	2027	2077	1997	1812	1546	1194	756

-2500	-2000	-1500	-1000	-500	0	500	1000	1500	USD/ha

Ymax (t/ha); *Nrate* (kgN/ha)
Current ranges; **Optimum rate**

GDP=Gross Domestic Product; CP=Cereal price (USD/kg); FP=Fertilizer price (USD/kgN); FC=Fixed cultivation cost (USD/ha); EC=External N cost (USD/kgN Surplus)

***Fig SI 27 Surface plots of economic benefits** as a function of N fertilizer rate and Ymax for farm (LHS) and benefits corrected for cost of N pollution as a proxy for benefits of society (RHS). Europe*

Farm level	National society level

China: GDP=3200 USD/cap

Net farm benefit USD/ha

Ymax/Nrate	25	50	75	100	125	150	175	200	225	250	275	300	325	350
2.0	78	158	225	277	316	341	352	350	333	303	259	201	129	43
2.5	107	214	304	376	431	468	488	491	477	445	397	330	247	146
3.0	137	270	383	474	545	595	625	633	621	588	534	460	364	249
3.5	167	326	462	573	660	722	761	775	765	730	672	589	482	351
4.0	197	383	541	671	774	849	897	916	908	873	809	719	600	454
4.5	227	439	620	770	889	976	1033	1058	1052	1015	947	848	718	556
5.0	256	495	699	868	1003	1103	1169	1200	1196	1158	1085	977	835	659
5.5	286	551	778	967	1117	1230	1305	1341	1340	1300	1222	1107	953	761
6.0	316	607	857	1065	1232	1357	1441	1483	1483	1443	1360	1236	1071	864
6.5	346	663	936	1164	1346	1484	1577	1624	1627	1585	1498	1366	1188	966
7.0	376	720	1015	1262	1461	1611	1713	1766	1771	1727	1635	1495	1306	1069
7.5	406	776	1094	1361	1575	1738	1849	1908	1915	1870	1773	1624	1424	1171
8.0	435	832	1173	1459	1690	1865	1985	2049	2058	2012	1911	1754	1541	1274
8.5	465	888	1252	1558	1804	1992	2121	2191	2202	2155	2048	1883	1659	1376
9.0	495	944	1331	1656	1919	2119	2257	2333	2346	2297	2186	2013	1777	1479
9.5	525	1000	1410	1755	2033	2246	2393	2474	2490	2440	2324	2142	1895	1581
10.0	555	1057	1489	1853	2148	2373	2529	2616	2633	2582	2461	2271	2012	1684
10.5	584	1113	1569	1952	2262	2500	2665	2757	2777	2724	2599	2401	2130	1786
11.0	614	1169	1648	2050	2377	2627	2801	2899	2921	2867	2737	2530	2248	1889
11.5	644	1225	1727	2149	2491	2754	2937	3041	3065	3009	2874	2660	2365	1992
12.0	674	1281	1806	2247	2605	2881	3073	3182	3209	3152	3012	2789	2483	2094

CP=0.28; FP=1; FC=57; EC=1.1

Net societal benefit USD/ha

Ymax/Nrate	25	50	75	100	125	150	175	200	225	250	275	300	325	350
2.0	58	118	165	197	214	216	204	177	135	78	5	-84	-187	-307
2.5	90	179	250	303	338	354	352	332	292	234	156	59	-57	-193
3.0	122	239	334	409	461	492	500	486	449	390	308	202	73	-80
3.5	153	299	419	514	584	629	647	640	606	546	458	344	203	34
4.0	185	358	503	620	707	765	794	793	762	701	609	486	332	147
4.5	216	418	587	725	830	902	941	946	918	856	759	628	461	260
5.0	248	477	671	830	952	1038	1087	1099	1073	1010	909	769	590	372
5.5	279	537	755	934	1074	1174	1233	1251	1229	1164	1058	910	719	485
6.0	311	596	839	1039	1196	1309	1378	1403	1383	1318	1207	1051	847	597
6.5	342	655	922	1143	1317	1444	1524	1555	1538	1472	1356	1191	975	709
7.0	373	714	1005	1247	1438	1579	1669	1706	1692	1625	1505	1331	1103	820
7.5	404	773	1088	1351	1559	1714	1813	1857	1846	1778	1653	1470	1230	932
8.0	435	831	1171	1454	1680	1848	1957	2008	1999	1930	1800	1610	1357	1043
8.5	464	887	1251	1557	1800	1982	2101	2158	2152	2082	1948	1749	1484	1153
9.0	493	941	1327	1652	1917	2115	2245	2308	2305	2234	2095	1887	1611	1264
9.5	522	995	1403	1746	2026	2243	2388	2458	2457	2385	2242	2026	1737	1374
10.0	551	1049	1479	1841	2136	2364	2527	2607	2609	2536	2388	2164	1863	1484
10.5	580	1104	1555	1936	2246	2486	2657	2756	2760	2687	2534	2301	1988	1594
11.0	609	1158	1632	2031	2356	2607	2787	2894	2911	2837	2680	2439	2114	1704
11.5	638	1213	1709	2126	2466	2729	2917	3029	3062	2987	2825	2576	2239	1813
12.0	667	1268	1785	2222	2577	2852	3047	3165	3204	3136	2970	2713	2363	1922

India: GDP=1030 USD/cap

Net farm benefit USD/ha

Ymax/Nrate	25	50	75	100	125	150	175	200	225	250	275	300	325	350
2.0	82	132	173	203	223	234	235	226	207	178	139	91	33	-36
2.5	104	172	229	273	305	324	332	327	309	279	237	183	116	37
3.0	125	212	285	343	386	415	428	427	411	381	335	275	200	110
3.5	146	252	341	413	468	505	525	528	514	482	433	367	284	183
4.0	167	292	398	483	549	596	622	629	616	584	531	459	368	256
4.5	189	332	454	553	631	686	719	730	718	685	629	551	451	329
5.0	210	372	510	624	712	776	816	831	821	786	727	644	535	402
5.5	231	412	567	694	794	867	913	931	923	888	825	736	619	475
6.0	252	452	623	764	875	957	1009	1032	1025	989	923	828	703	548
6.5	273	492	679	834	957	1047	1106	1133	1128	1090	1021	920	787	621
7.0	295	532	735	904	1038	1138	1203	1234	1230	1192	1119	1012	870	694
7.5	316	572	792	974	1120	1228	1300	1335	1332	1293	1217	1104	954	767
8.0	337	612	848	1044	1201	1319	1397	1435	1435	1395	1315	1196	1038	840
8.5	358	652	904	1114	1283	1409	1493	1536	1537	1496	1413	1288	1122	913
9.0	379	692	960	1184	1364	1499	1590	1637	1639	1597	1511	1380	1205	986
9.5	401	732	1017	1254	1445	1590	1687	1738	1742	1699	1609	1472	1289	1059
10.0	422	772	1073	1325	1527	1680	1784	1839	1844	1800	1707	1565	1373	1132
10.5	443	812	1129	1395	1608	1770	1881	1939	1946	1901	1805	1657	1457	1205
11.0	464	852	1185	1465	1690	1861	1978	2040	2049	2003	1903	1749	1540	1278
11.5	486	892	1242	1535	1771	1951	2074	2141	2151	2104	2001	1841	1624	1351
12.0	507	932	1298	1605	1853	2042	2171	2242	2253	2206	2099	1933	1708	1424

CP=0.2; FP=1; FC=17; EC=0.4

Net societal benefit USD/ha

Ymax/Nrate	25	50	75	100	125	150	175	200	225	250	275	300	325	350
2.0	76	120	154	178	192	196	189	173	146	109	61	3	-65	-144
2.5	98	161	212	251	276	289	290	277	252	214	163	100	23	-67
3.0	120	203	270	323	360	383	390	382	359	320	266	196	110	9
3.5	142	244	328	395	445	476	490	487	465	425	368	292	198	86
4.0	164	285	386	467	529	570	591	591	571	531	470	388	285	162
4.5	185	326	444	540	613	663	691	695	677	636	571	484	373	238
5.0	207	367	502	612	697	756	791	800	783	741	673	579	460	314
5.5	229	408	559	684	780	849	890	904	889	846	775	675	547	390
6.0	250	449	617	756	864	942	990	1008	995	951	876	771	634	466
6.5	272	490	675	827	948	1035	1090	1112	1100	1056	978	866	721	542
7.0	294	530	732	899	1031	1128	1189	1215	1206	1160	1079	961	808	618
7.5	315	571	790	971	1115	1221	1289	1319	1311	1265	1180	1057	894	693
8.0	337	612	847	1043	1198	1313	1388	1423	1416	1369	1281	1152	981	769
8.5	358	652	904	1114	1281	1406	1487	1526	1521	1474	1382	1247	1068	844
9.0	379	691	959	1183	1364	1498	1587	1629	1627	1578	1483	1342	1154	920
9.5	400	730	1014	1252	1443	1589	1686	1733	1731	1682	1584	1437	1241	995
10.0	421	770	1070	1321	1523	1677	1783	1836	1836	1786	1684	1531	1327	1071
10.5	442	809	1125	1390	1603	1766	1878	1939	1941	1890	1785	1626	1413	1146
11.0	463	849	1181	1459	1683	1855	1973	2039	2046	1994	1885	1721	1499	1221
11.5	484	888	1236	1528	1764	1944	2068	2137	2150	2097	1986	1815	1585	1296
12.0	505	928	1292	1597	1844	2033	2163	2236	2252	2201	2086	1909	1671	1371

Kenya: GDP=1160 USD/cap

Net farm benefit USD/ha

Ymax/Nrate	25	50	75	100	125	150	175	200	225	250	275	300	325	350
2.0	88	160	216	256	280	288	280	255	215	159	87	-1	-105	-225
2.5	123	225	307	370	412	434	437	419	382	324	246	148	31	-107
3.0	157	290	399	484	544	581	594	583	548	489	405	298	167	12
3.5	192	355	490	598	677	728	751	747	714	653	565	448	303	130
4.0	226	420	582	711	809	875	909	911	880	818	724	597	439	249
4.5	261	485	673	825	942	1022	1066	1074	1047	983	883	747	575	367
5.0	295	550	765	939	1074	1169	1223	1238	1213	1148	1042	897	711	486
5.5	329	615	856	1053	1206	1316	1381	1402	1379	1312	1201	1046	848	605
6.0	364	680	947	1167	1339	1462	1538	1566	1545	1477	1361	1196	984	723
6.5	398	745	1039	1281	1471	1609	1695	1730	1712	1642	1520	1346	1120	842
7.0	433	810	1130	1395	1604	1756	1853	1893	1878	1806	1679	1495	1256	960
7.5	467	875	1222	1509	1736	1903	2010	2057	2044	1971	1838	1645	1392	1079
8.0	502	940	1313	1623	1868	2050	2167	2221	2210	2136	1997	1795	1528	1198
8.5	536	1004	1405	1737	2001	2197	2325	2385	2377	2301	2157	1944	1664	1316
9.0	571	1069	1496	1851	2133	2344	2482	2549	2543	2465	2316	2094	1800	1435
9.5	605	1134	1587	1965	2266	2491	2639	2712	2709	2630	2475	2244	1937	1553
10.0	640	1199	1679	2078	2398	2637	2797	2876	2876	2795	2634	2393	2073	1672
10.5	674	1264	1770	2192	2530	2784	2954	3040	3042	2960	2793	2543	2209	1790
11.0	709	1329	1862	2306	2663	2931	3111	3204	3208	3124	2953	2693	2345	1909
11.5	743	1394	1953	2420	2795	3078	3269	3368	3374	3289	3112	2842	2481	2028
12.0	778	1459	2045	2534	2927	3225	3426	3531	3541	3454	3271	2992	2617	2146

CP=0.33; FP=2; FC=20; EC=0.4

Net societal benefit USD/ha

Ymax/Nrate	25	50	75	100	125	150	175	200	225	250	275	300	325	350
2.0	81	146	195	228	244	244	228	195	146	80	-2	-101	-216	-348
2.5	116	213	288	344	380	395	389	363	317	250	162	54	-75	-226
3.0	152	279	382	461	515	545	551	531	488	419	326	208	65	-103
3.5	187	345	475	577	651	695	712	700	659	589	490	362	205	19
4.0	222	411	569	693	786	846	873	867	829	758	654	516	346	142
4.5	257	477	662	810	921	996	1034	1035	1000	927	817	670	486	264
5.0	292	544	755	926	1056	1146	1195	1203	1170	1096	981	824	626	386
5.5	327	610	848	1042	1191	1296	1356	1371	1340	1265	1144	978	766	508
6.0	362	676	941	1158	1326	1446	1516	1538	1510	1434	1307	1131	905	630
6.5	397	742	1034	1274	1461	1595	1677	1705	1680	1602	1470	1285	1045	752
7.0	432	808	1127	1390	1596	1745	1837	1872	1850	1771	1633	1438	1185	873
7.5	467	873	1220	1505	1730	1895	1998	2040	2020	1939	1796	1591	1324	995
8.0	502	939	1312	1621	1865	2044	2158	2207	2190	2107	1959	1744	1464	1117
8.5	536	1004	1404	1737	1999	2193	2318	2373	2359	2275	2121	1897	1603	1238
9.0	570	1068	1494	1849	2133	2342	2478	2540	2529	2443	2284	2050	1742	1360
9.5	604	1132	1585	1962	2263	2490	2638	2707	2698	2611	2446	2203	1881	1481
10.0	638	1197	1675	2074	2394	2634	2796	2873	2867	2779	2609	2356	2020	1602
10.5	673	1261	1766	2187	2525	2779	2951	3039	3036	2946	2771	2508	2159	1723
11.0	707	1325	1856	2299	2655	2924	3106	3202	3205	3114	2933	2661	2298	1844
11.5	741	1390	1947	2412	2786	3069	3262	3364	3373	3281	3095	2813	2437	1965
12.0	775	1454	2038	2525	2917	3215	3417	3525	3539	3448	3256	2965	2575	2086

-2500	-2000	-1500	-1000	-500	0	500	1000	1500	USD/ha

Ymax (t/ha); *Nrate* (kgN/ha)

Current ranges; **Optimum rate**

GDP=Gross Domestic Product; CP=Cereal price (USD/kg); FP=Fertilizer price (USD/kgN); FC=Fixed cultivation cost (USD/ha); EC=External N cost (USD/kgN Surplus)

Fig SI 28 Surface plots of economic benefits *as a function of N fertilizer rate and Ymax for farm (LHS) and benefits corrected for cost of N pollution as a proxy for benefits of society (RHS). China, India and Kenya as examples of Developing countries.*

We also did a sensitivity analysis (fixed relative variation of parameter) and uncertainty analysis (realistic nominal variation of parameters) to explore the effect of changes in supply and prices on *EONR* and *SONR*. For the low case of the sensitivity analysis the sign of the parameter variation is such that *EONR* of *SONR* is reduced (e.g. an increase of *SN* reduces *EONR*) and for the high case vice versa.

The reference case is Northwest European wheat (*GDP* 50 kUS$/capita and *Ymax* 8 t/ha), and a mid income case (*GDP* 10 kUS$/capita and *Ymax* 4 t/ha). Regarding the sensitivity to the shape of the LT N response curve, a +/-20% variation is not possible. Instead we selected a 20% lower, respectively, higher curve relative to the generic response curve (which is the median), from the ranked population of the used total set of 26 LT curves. The 20% lower, respectively, higher curves are Curve 6 and 21 – see Table SI 2. For the uncertainty analysis we used Curve 2 and 25 which are an approximation of the lower and upper bound of the 80% confidence interval of the ranked population of the used total set of 26 LT curves. Results are summarized below.

Summary of sensitivity and uncertainty analysis for EONR								
			EONR=198 kgN/ha			Ymax=4 t/ha, EONR=181 kgN/ha		
			Sensitivity EONR (% change of EONR for 1% of parameter)					
	Value	Sensitivity	-20%	+20%	Mean	-20%	+20%	Mean
SN (input other N sources kgN/ha)	20	20%	0.10	-0.10	0.10	0.11	-0.11	0.11
Ymax (max yield ton grain/ha)	8	20%	-0.11	0.07	0.09	-0.23	0.15	0.19
PN (price N fertilizer US$/kgN)	1	20%	0.08	-0.08	0.08	0.18	-0.18	0.18
PY (grain price US$/ton)	200	20%	-0.11	0.07	0.09	-0.23	0.15	0.19
Curve shape	-	Curve 6, 21	0.19	-0.68	0.43	0.67	-0.28	0.47
Total			0.17	-0.72	0.45	0.01	-0.63	0.32
			Uncertainty EONR (kgN/ha)					
	Value	Uncertainty	low	high	Mean	low	high	Mean
SN (input other N sources kgN/ha)	20	25%	-5.0	5.0	5.0	-5.0	5.0	5.0
Ymax (max yield ton grain/ha)	8	20%	-4.2	2.8	3.5	-8.4	5.6	7.0
PN (price N fertilizer US$/kgN)	1	10%	-1.7	1.7	1.7	-3.3	3.3	3.3
PY (grain price US$/ton)	200	20%	-4.2	2.8	3.5	-8.4	5.6	7.0
Curve shape	-	Curve 2, 25	-49.6	17.5	33.6	-32.9	34.2	33.6
Total			-61.6	30.2	45.9	-61.7	34.1	47.9

Summary of sensitivity and uncertainty analysis for SONR								
			SONR=154 kgN/ha			Ymax=4 t/ha, GDP 10 kUS$/cap: SONR=133 kgN/ha		
			Sensitivity SONR (% change of SONR for 1% of parameter)					
	Value	Sensitivity	-20%	+20%	Mean	-20%	+20%	Mean
SN (input other N sources kgN/ha)	20	20%	0.15	-0.15	0.15	0.18	-0.18	0.18
Ymax (max yield ton grain/ha)	8	20%	-1.22	0.74	0.98	-1.37	0.88	1.12
PN (price N fertilizer US$/kgN)	1	20%	0.04	-0.04	0.04	0.22	-0.23	0.23
PY (grain price US$/ton)	200	20%	-0.23	0.20	0.21	-0.82	0.59	0.70
GDP (1000 US$/cap)	50	20%	0.20	-0.16	0.18	0.49	2.41	1.45
Curve shape	-	Curve 6, 21	-0.05	-0.52	0.29	-0.13	1.62	0.87
Total			-1.29	-0.10	0.70	-1.47	0.11	0.79
			Uncertainty SONR (kgN/ha)					
	Value	Uncertainty	low	high	Mean	low	high	Mean
SN (input other N sources kgN/ha)	20	25%	-5	5	5.0	-5	5	5
Ymax (max yield ton grain/ha)	8	20%	-32.58	19.83	26.2	-31.0	19.9	25.4
PN (price N fertilizer US$/kgN)	1	10%	-0.59	0.59	0.6	-2.6	2.6	2.6
PY (grain price US$/ton)	200	20%	-6.16	5.24	5.7	-18.5	13.3	15.9
GDP (1000 US$/cap)	50	5%	-1.13	1.20	1.2	7.3	12.7	10.0
Curve shape	-	Curve 2, 25	-23.82	1.63	12.7	-4.6	7.6	6.1
Total			-52.97	38.07	45.5	-55.4	45.0	50.2

Table SI 6 Summary of results of sensitivity and uncertainty analysis on the economic (EONR) and societal (SONR) optimal N rate

EONR is most sensitivity to the shape of the LT N response curve. Sensitivity of *EONR* to the cereal price is two to five times lower and comparable to sensitivity of *EONR* to *SN* and *Ymax*, but sensitivity to price increases with decreasing *Ymax*. Also uncertainty in *EONR* is dominated by uncertainty in the N response function; here we assumed an uncertainty in the cereal price of 20%, which likely is an overestimate.

SONR is most sensitive to *Ymax* for a case with high *GDP and Ymax*, while for the case with lower *GDP and Ymax*, sensitivity to *GDP* and curve shape increases. Regarding uncertainty of *SONR*, *Ymax* is also the dominant source of uncertainty. Uncertainty in the cereal price is the 3rd most important factor for the case with *GDP* =50 and *Ymax* =8, and the 2nd factor for the case with *GDP* =10 and *Ymax* =4. This uncertainty analysis demonstrates that the contribution of uncertainty of the cereal price to uncertainty in *SONR* (respectively 13% and 32% for *Ymax* is 8 and 4 t/ha) is relevant but the contribution of uncertainty in *Ymax* is dominant.

The calculated maximum ranges of *EONR* and *SONR* are close to the +/- 50 kgN/ha and wide. Uncertainty ranges s in *EONR, SONR* and associated yields turn out lower if parameters are varied randomly in a Monte Carlo analysis, and assuming normal distributions.

	Mean	SDEV
SN (input other N sources kgN/ha)	20	2.5
Ymax (max yield ton grain/ha)	8	0.8
PN (price N fertilizer US$/kgN)	1	0.1
PY (grain price US$/ton)	200	20
GDP (1000 US$/cap)	50	1.25
a	-1.87E-05	4.90E-06
b	8.77E-03	*

Table SI 7 Statistics of uncertainties in parameters determining EONR and SONR

** The standard deviation (SDEV) of the coefficient [a] of the fitted LT N response was based on the variation of this coefficient for the 25 individual curves, and the parameter [b] was derived from the quadratic regression between [a] and [b] (R2 = 0.999).*

Ymax		EONR (added synt) kgN/ha)				Yield at EONR (t/ha)		
	mean	stdev	10th	90th	mean	stdev	10th	90th
8.0	198	16	180	218	8.1	0.1	8.0	8.2
4.0	180	13	166	199	4.0	0.1	3.9	4.1
		SONR (added synt) kgN/ha)				Yield at SONR (t/ha)		
8.0	128	17	131	173	7.1	0.4	6.6	7.7
4.0	104	16	110	151	3.2	0.3	2.9	3.5

Table SI 8 Mean, standard deviation and 10th and 90th percentiles of EONR, SONR and associated yields due to uncertainty in SN, Ymax, prices of cereal and fertilizer, GDP and shape of LT N response curve, using Monte Carlo analysis (N=100) and assuming normal distributions.

Standard deviations for *EONR* and *SONR*, 8% and 15% of the mean, respectively, are modest and calculated 80% confidence intervals of *EONR* and *SONR* (Table SI 8) indicate that our calculated safe operating ranges as in Figure 7 of main paper are quite robust. Standard deviations for yields at *EONR* and *SONR*, respectively 1-2% and 6-8% are much smaller than for *EONR* and *SONR*, in view of diminishing returns on *Nav*. This indicates that accounting for changes in cereal prices due to changes in cereal production in a certain region has no large effect on production of cereals under *SONR*, and therefore also not on total supply in that region. The effect would be even smaller when accounting for buffering effects of cereal supply-price effects in other regions and for trade.

Note 13 Farm gate and food plate prices of cereals

For economic analysis for the farming sector the farm gate price of cereals is used. Cereals as traded at farm gate represent unmilled (rough) grain where distinction can be made between different qualities, with higher prices for grains used for bread, pasta and beer. Here we use global import prices of wheat as a proxy for the farm gate price. For many countries with well-developed storage and transport infrastructure the cost increase between farm gate and harbor of import is small. The mean global price for imported unmilled wheat grain for all countries over 2014-2018, excluding durum and meslin, was 0.22 USD/kg and for durum 0.31 USD/kg (Resource Trade, http://resourcetrade.earth). For the majority of countries, the importer price lies between 0.18 and 0.24 USD/kg grain. Countries with much higher prices often are islands or small land locked countries with very low import quantities.

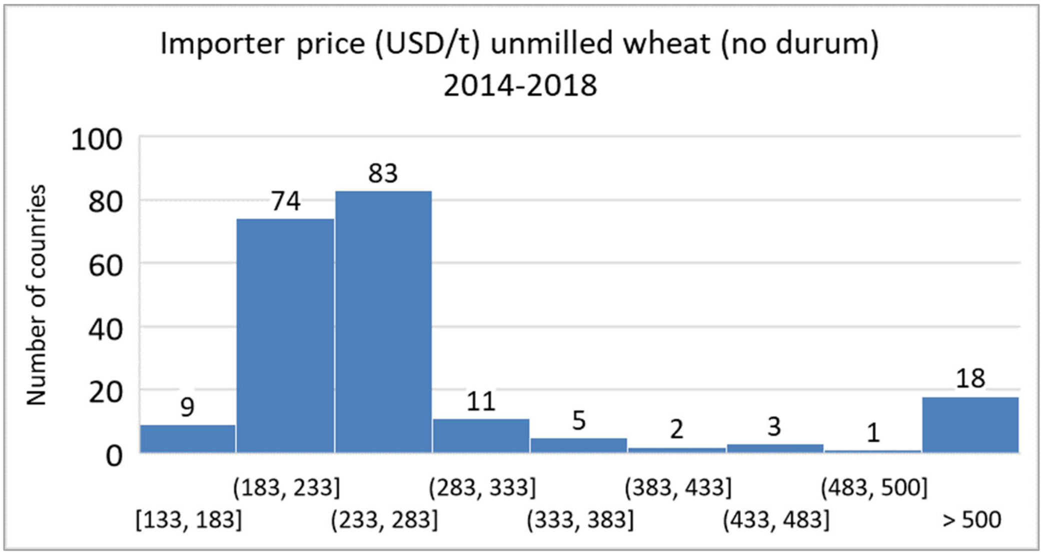

Fig SI 29 Distribution of national import prices of unmilled wheat *(http://resourcetrade.earth, visited on October 27, 2020).*

For economic analysis at society scale we included a scenario that considers that virtual prices for cereals paid by consumers, referred to as “food plate” prices, are higher than farm gate prices. Food plate prices of cereal are virtual as most consumers do not buy unmilled grain, but food products based on grain, where processing of grain, in the form of milling, drying, doughmaking, flavoring and baking add value. Derivation of virtual price of grain based on the consumer price of cereal product therefore involves many assumptions. A default value price ratio of one of food plate over farm gate would be justified if we assume that all added value is a compensation for the cost of processing. Price ratio exceeding one assume that after correcting for all fixed and variable costs of processing cereals, a net benefit remains that represents additional welfare in the form of profit for investors, budget for investment and innovation. Apart from this added value, the cost of labor for processing also represents benefit of employment.

We used two approaches for the ratio of food plate price over farm gate price:

[A] A lumped regional approach based on the relative increase of gross added value of agricultural production created by food processing in the EU and the USA.

EU case: In 2012 total added value in food supply was 831 billion euro, of which 206 in agriculture, 219 in processing and 406 billion euro in food retail and services. This would give a price ratio of 4.0 of food plate over farm gate, and of 2.1 when not including retail and food services.

Graph 2 Contribution of the different stages of the food chain to the European economy, EU-28, 2012

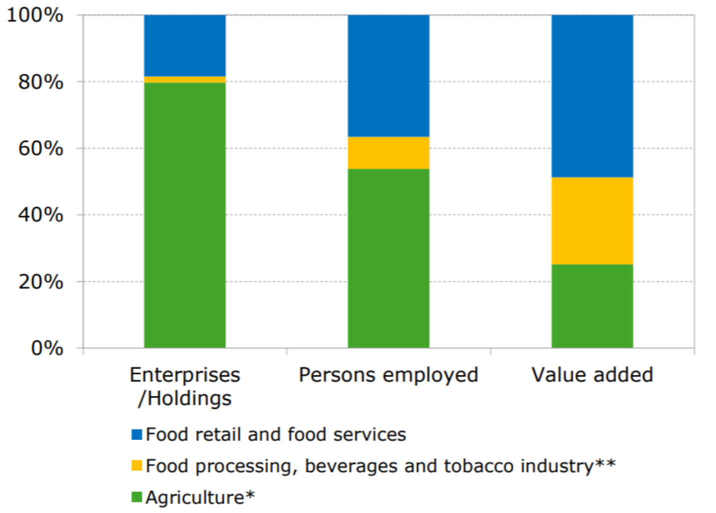

Sources: Eurostat, National Accounts (nama_nace64_c), Farm Structure Survey (ef_kvaareg and ef_olfftecs), Economic Accounts for Agriculture (aact_eaa01) and Structural Business Statistics (sbs_na_sca_r2, sbs_na_dt_r2 sbs_na_dt_r2 and sbs_na_1a_se_r2).
* 2010 data for holdings and persons employed in agriculture.
** Estimated EU-28 total for 2012.

Fig SI 30 Value added by agriculture and food industry in the EU

	Added value	Price ratio
	billion Euro	Food/farm
Food supply	831	4.0
Agriculture	206	
Processing	219	2.1
Retail & services	406	

Table SI 9 Derivation of food plate to farm gate price ratio for the EU
https://ec.europa.eu/info/sites/info/files/food-farming-fisheries/trade/documents/agri-market-brief-04_en.pdf

USA case: In 2012 total added in food supply was 825 billion USD (including beverages and tobacco products), of which 149 in agriculture, 227 in processing and 448 billion euro in food retail and services. This would give a price ratio of 5.5 of food plate over farm gate, and of 2.5 when not including retail and food services. Ratio's based on 2019 values are 8.0 and 3.1, respectively.

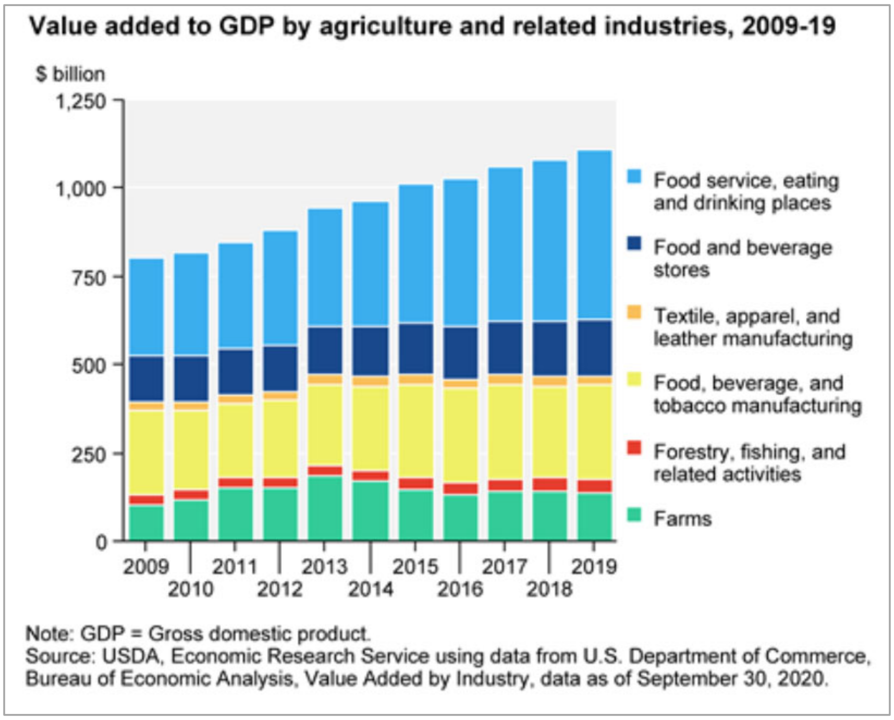

Fig SI 31 Value added by agriculture and food industry in the USA

	Added value		Price ratio food/farm	
	2012	2019	2012	2019
	billion USD			
Food supply	825	1032	5.5	8.0
Agriculture	149	130		
Processing	227	273	2.5	3.1
Retail	130	156		
Services	318	474		

Table SI 10 Derivation of food plate to farm gate price ratio for the USA

https://www.ers.usda.gov/data-products/ag-and-food-statistics-charting-the-essentials/ag-and-food-sectors-and-the-economy/

[B] A specific local approach the price share of wheat grain in the consumer price of Dutch bread

Ratio's between (virtual) food plate prices and farm gate prices will depend on the type of product. As a representative example for a cereal based food product we estimated the price ratio for bread in the Netherlands. Baltussen et al., (2014) calculated the relative contributions of primary agriculture, wholesale, processing and retail to consumer prices in the Netherlands, which ranged between 15% for bread to 35% for paprika. Bread prices in the Netherlands range between 1 euro/kg in supermarkets to 4.5 euro/kg in bakeries (25% of total volume). Based on the 15% contribution this would infer a "food plate" price of cereal for bread between 0.3 and 0.7 euro/kg/grain. Using a farm gate price of bread wheat of 0.26 euro/kg, this would imply a ratio of food plate of farm gate of 1.2-2.7; the weighted ratio is 1.6, taking into account that 75% of bread is bought in supermarkets. This weighted mean values is much lower than based on added value in macro economy.

We also made a very provisional calculation for the price ratio for use of feed cereals for pork production. This calculation involves more assumptions and estimates of the ratio are around five.

Despite these arguments for using prices ratios exceeding one and approximations of these ratios, using these price ratio's is not common in welfare economic analysis and no publications were found. Results for safe operating N fertilizer spaces using price ratios much higher than one are therefore not conclusive and given here to stimulate discussion.

We also looked into the effect of cereal supply on prices and demand for cereal food products. Andreyeva (2010) reports a price elasticity for cereal products of 0.6 in the USA. From this we infer that a change of cereal supply of 10% will increase the price of cereal by 17% (1/0.6). Given that the farm gate price of cereals constitutes only 8% of the prices of food cereals in the US (Schnepf, 2015), and 15% of the bread price in the Netherlands, a 10% decrease of cereal supply would increase the consumer price by about 3%, and then would have hardly an effect on consumption and demand. However, Schnepf (2015) also writes that the transmission of changes in farm gate prices to consumer prices is complex and can be very different between regions. We further quote Andreyeva (2010) to illustrate the effect of price on demand: "It is determined by a multitude of factors: availability of substitutes, household income, consumer preferences, expected duration of price change, and the product's share of a household's income." Another uncertainty and opportunity for the effect of a decreasing regional supply of cereals on prices and consumption is the current massive waste of cereals in the agro-food systems, and the potential to reduce this waste.

Additional reference:

Andreyeva, T., Long, M. W., & Brownell, K. D. (2010). The impact of food prices on consumption: a systematic review of research on the price elasticity of demand for food. American journal of public health, 100(2), 216-222.

Baltussen, W. H. M., Kornelis, M., van Galen, M. A., Logatcheva, K., van Horne, P. L. M., Smit, A. B., ... & Pham, T. M. L. (2014). Prijsvorming van voedsel; Ontwikkelingen van prijzen in acht Nederlandse ketens van versproducten (No. 14-112). LEI Wageningen UR.

Schnepf, R. D. (2015). Farm-to-food price dynamics. Washington, DC: Congressional Research Service.

Note 14 Implications of using LT N response for global maize cultivation

To investigate the implications of using long term N response curves for global cereal production we constructed global equivalent response curves for global absolute maize production as a function of global consumption of mineral fertilizer using the approach as used in the global yield gap analysis by Mueller et al. (2012)[52], replacing the statistical relation between yield and fertilizer consumption around the year 2000 as derived from national data by two alternatives, (1) our indexed generic N response curve for global cereals (Eq. 2 main article) with a small yield intercept and (2) the long term N response for US maize based on LTE of around 20 years, closer to but not with a steady state soil N pool causing a higher yield intercept (Figure SI 5B)). Using the near long-term response curve [1] still leads to an overestimation of long-term global maize yield at a given level of N consumption of around 120 Mton. Conversely, the medium LT curve [1] leads to an underestimation of N need by 10 Mton, for a long term given global maize yield target of 700 Mton.

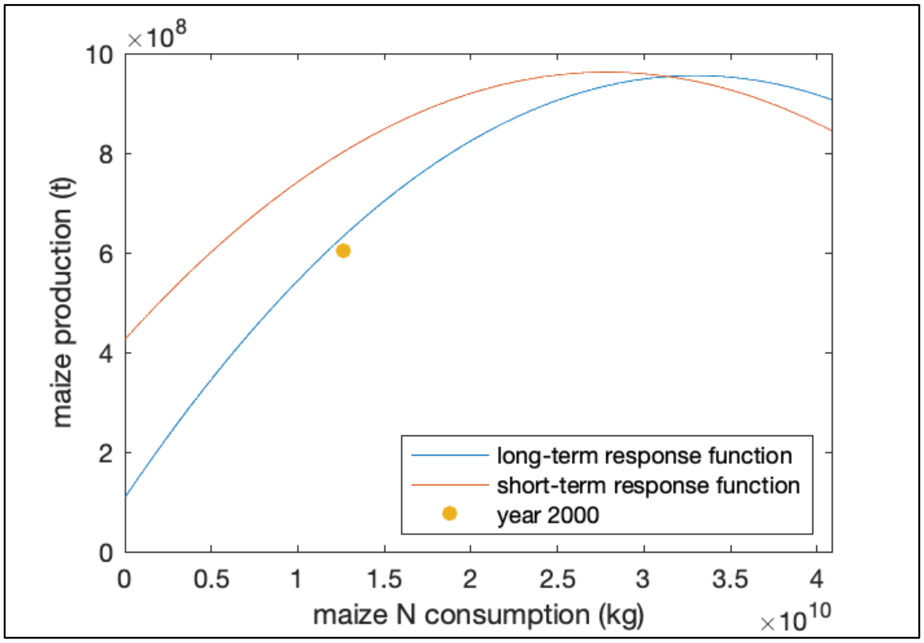

Fig. SI 32 Implications of using long term (steady state) and medium long term (20 yr) yield response functions to mineral N for global maize production *based on the grid methods as used in Mueller et al. (2012)[52].*
