# Supplementary material for: Establishing long-term nitrogen response of global cereals to assess sustainable fertilizer rates
Source: Nat Food. 2022 Jan 31;3(2):122–32. doi: 10.1038/s43016-021-00447-x (PMC10661743; doi:10.1038/s43016-021-00447-x)
Supplement: Supplementary file 2 — Reporting Summary. [file 43016_2021_447_MOESM2_ESM.pdf]

nature research

Corresponding author(s): HJM van Grinsven, NATFOOD-20071311

Last updated by author(s): Aug 14, 2020

Reporting Summary

Nature Research wishes to improve the reproducibility of the work that we publish. This form provides structure for consistency and transparency in reporting. For further information on Nature Research policies, see our Editorial Policies and the Editorial Policy Checklist.

Statistics

For all statistical analyses, confirm that the following items are present in the figure legend, table legend, main text, or Methods section.

n/a	Confirmed	
☐	☒	The exact sample size (*n*) for each experimental group/condition, given as a discrete number and unit of measurement
☐	☒	A statement on whether measurements were taken from distinct samples or whether the same sample was measured repeatedly
☒	☐	The statistical test(s) used AND whether they are one- or two-sided<br>*Only common tests should be described solely by name; describe more complex techniques in the Methods section.*
☒	☐	A description of all covariates tested
☒	☐	A description of any assumptions or corrections, such as tests of normality and adjustment for multiple comparisons
☐	☒	A full description of the statistical parameters including central tendency (e.g. means) or other basic estimates (e.g. regression coefficient) AND variation (e.g. standard deviation) or associated estimates of uncertainty (e.g. confidence intervals)
☒	☐	For null hypothesis testing, the test statistic (e.g. *F*, *t*, *r*) with confidence intervals, effect sizes, degrees of freedom and *P* value noted<br>*Give P values as exact values whenever suitable.*
☒	☐	For Bayesian analysis, information on the choice of priors and Markov chain Monte Carlo settings
☒	☐	For hierarchical and complex designs, identification of the appropriate level for tests and full reporting of outcomes
☒	☐	Estimates of effect sizes (e.g. Cohen's *d*, Pearson's *r*), indicating how they were calculated

Our web collection on statistics for biologists contains articles on many of the points above.

Software and code

Policy information about availability of computer code

Data collection	n/a
Data analysis	Excel was used for descriptive statistics, regression and derivation of optimum N rates

For manuscripts utilizing custom algorithms or software that are central to the research but not yet described in published literature, software must be made available to editors and reviewers. We strongly encourage code deposition in a community repository (e.g. GitHub). See the Nature Research guidelines for submitting code & software for further information.

Data

Policy information about availability of data

All manuscripts must include a data availability statement. This statement should provide the following information, where applicable:

- Accession codes, unique identifiers, or web links for publicly available datasets
- A list of figures that have associated raw data
- A description of any restrictions on data availability

Summaries of N response data for Broadbalk Winter wheat trials at Rothamsted Experimental Station and for global cereals as used for figures and regression analysis are available upon reasonable request. Selections of original observations for Broadbalk are available via the electronic Rothamsted Archive (http://www.era.rothamsted.ac.uk/).

Field-specific reporting

Please select the one below that is the best fit for your research. If you are not sure, read the appropriate sections before making your selection.

☐ Life sciences ☐ Behavioural & social sciences ☒ Ecological, evolutionary & environmental sciences

For a reference copy of the document with all sections, see nature.com/documents/nr-reporting-summary-flat.pdf

Ecological, evolutionary & environmental sciences study design

All studies must disclose on these points even when the disclosure is negative.

Study description	This study derives generic long-term response relationships between grain yield per hectare and input of nitrogen (N) per hectare. For this we use observed grain yield from published long-term field trials, with stepped N rates, for wheat, maize, barley and rice in contrasting world regions. Trials have a duration of at least 15 years, during which treatments (N rates) remain assigned to fixed plots. This minimum duration is to approach steady state of the soil N pool. N rates in these trials range between zero and values to obtain maximum yields. We did not use observation from the common type of field trials lasting 1-2 years, where soil N not in steady state which causes bias in the N response. A universal N response relationship for global cereals was derived by linear regression on scaled data pooled from individual trials into a single set. We first fitted 2nd order polynomials to observations of individual trials to obtain the scaling parameters. Observed grain yields for each trial were normalized by dividing by the maximum yields as obtained from the regression. The N fertilizer inputs for each trial were transformed to total net N input by adding supplementary N inputs (SN) from non-fertilizer sources, where SN is the (negative value of the) intercept of yield with the horizontal N fertilizer axis. The new set of scaled observations for wheat, maize, barley could be described by a new 2nd order polynomial with zero intercept, which represents our hypothesized generic and globally applicable response curves for cereals. This curve was tested and validated before application to derive agronomic efficiencies and economically optimal N fertilizer rates for contrasting world regions using local data on N fertilizer use and prices of crops and fertilizers.
Research sample	For the derivation of generic long-term response relationships we used published results for long term N response trials for wheat, maize, barley and rice from various sites in Europe, North America and Asia. Criteria for selection were (i) a trial duration of at least 15 years (including the initialization phase), (ii) adequate supply of other nutrients (a.o. phosphorus, potassium, magnesium) and (iii) supplementary N inputs (SN) from non-fertilizer sources not exceeding 100 kg N per hectare. An excess of 100 kg N per hectare from non-fertilizer sources indicates irregularities in trials not allowing derivation of yield response to N fertilizer input. We derived scaled generic response curves for three sets of trials. Firstly, observations for wheat in rotation at Rothamsted Research (UK) between 1986 and 2018, with seven N fertilizer steps. Secondly, observation from 25 trials for wheat, maize and barley in Europe, North America and Asia, with 3 to 7 N rate steps per trial. Thirdly, observations from four trials for lowland rice - wheat systems in India and Nepal, with 3-4 N rates per trial. Details per trial on soils, climate, time period and experimental setup are provided in Supplementary Table 1. For checking plausibility of our generic relationships for Sub-Saharan Africa we used both modelled results and observations from a few medium long-term field trials. For plausibility checking in Europe we used modelled results.
Sampling strategy	n/a, as we use published data from ongoing or completed field trials
Data collection	Data for the Broadbalk wheat experiment were provided by co-author Margaret Glendining but are available via the electronic Rothamsted Archive (http://www.era.rothamsted.ac.uk/). Data for other trials were obtained from literature, for Europe relying on an earlier publication in 2017 by co-author Renske Hijbeek.
Timing and spatial scale	Varies per trial. Most trials were carried out between 1980 and 2018. The spatial scale is a plot typically of 50-100 m2. We analyzed data from Europe, Asia, North America, and Sub-Saharan Africa.
Data exclusions	see selection criteria under Research sample
Reproducibility	n/a, as we use published data from ongoing or completed field trials
Randomization	n/a, we use published data from ongoing or completed long-term field trials. These generally run over many years or decades. Randomization of treatments occurs at the initial layout of the trial. The initial assignment of treatments to field plots then remains fixed for the duration of the trial. Treatments are generally replicated in a randomized block design.
Blinding	n/a, as we use published data from ongoing or completed field trials

Did the study involve field work? ☐ Yes ☒ No

Reporting for specific materials, systems and methods

We require information from authors about some types of materials, experimental systems and methods used in many studies. Here, indicate whether each material, system or method listed is relevant to your study. If you are not sure if a list item applies to your research, read the appropriate section before selecting a response.

Materials & experimental systems

n/a	Involved in the study
☒	☐ Antibodies
☒	☐ Eukaryotic cell lines
☒	☐ Palaeontology and archaeology
☒	☐ Animals and other organisms
☒	☐ Human research participants
☒	☐ Clinical data
☒	☐ Dual use research of concern

Methods

n/a	Involved in the study
☒	☐ ChIP-seq
☒	☐ Flow cytometry
☒	☐ MRI-based neuroimaging
